# The Virtual Tissues foundation model resolves spatial proteomics across scales

**DOI:** 10.1038/s41586-026-10884-y

**Published:** 2026-08-05

**Authors:** Johann Wenckstern, Eeshaan Jain, Benedikt von Querfurth, Yexiang Cheng, Kiril Vasilev, Matteo Pariset, Phil F. Cheng, Petros Liakopoulos, Olivier Michielin, Andreas Wicki, Gabriele Gut, Charlotte Bunne

**Affiliations:** 1 School of Computer and Communication Sciences, EPFL, Lausanne, Switzerland; 2 Department of Computer Science, ETH, Zurich, Switzerland; 3 Faculty of Medicine, University of Geneva, Geneva, Switzerland; 4 Department of Oncology, Geneva University Hospitals, Geneva, Switzerland; 5 Department of Medical Oncology and Hematology, University Hospital Zurich, Zurich, Switzerland; 6 University of Zurich, Faculty of Medicine, Zurich, Switzerland; 7 Swiss Institute for Experimental Cancer Research, School of Life Sciences, EPFL, Lausanne, Switzerland

**Keywords:** Machine learning, Cancer imaging, Predictive markers

## Abstract

Spatial proteomics technologies have transformed our understanding of complex tissue architecture in cancer but present unique challenges for computational analysis^1^. Each study uses a different marker panel and protocol, and most methods are tailored to single cohorts, which limits knowledge transfer and robust biomarker discovery. Here we present Virtual Tissues (VirTues), a general-purpose foundation model for spatial proteomics that learns marker-aware, multi-scale representations of proteins, cells, niches and tissues directly from multiplex imaging data. From a single pretrained backbone, VirTues supports marker reconstruction, cell segmentation and typing, niche annotation, spatial biomarker discovery and patient stratification, including zero-shot annotation across heterogeneous panels and datasets. In triple-negative breast cancer, VirTues-derived biomarkers predict anti-PD-L1 chemo-immunotherapy response^2^ and stratify disease-free survival in an independent cohort^3^, outperforming state-of-the-art biomarkers derived from the same datasets and current clinical stratification schemes.

## Abstract (web summary)

Virtual Tissues (VirTues), a foundation model for spatial proteomics that captures tissue organization across scales, supports marker reconstruction, cell segmentation and typing, niche annotation, spatial biomarker discovery and patient stratification across heterogeneous panels and datasets.

## Main

Tissues, particularly in cancer, display pronounced heterogeneity across patients, disease stages and within individual tumours, evident in diverse cell phenotypes, states and spatial organization^1^. Tumour development and therapy response depend not only on cancer cells but also on their interactions with the surrounding environment, making the spatial organization and function of the tumour microenvironment (TME) central to cancer treatment.

**Fig. 1:**
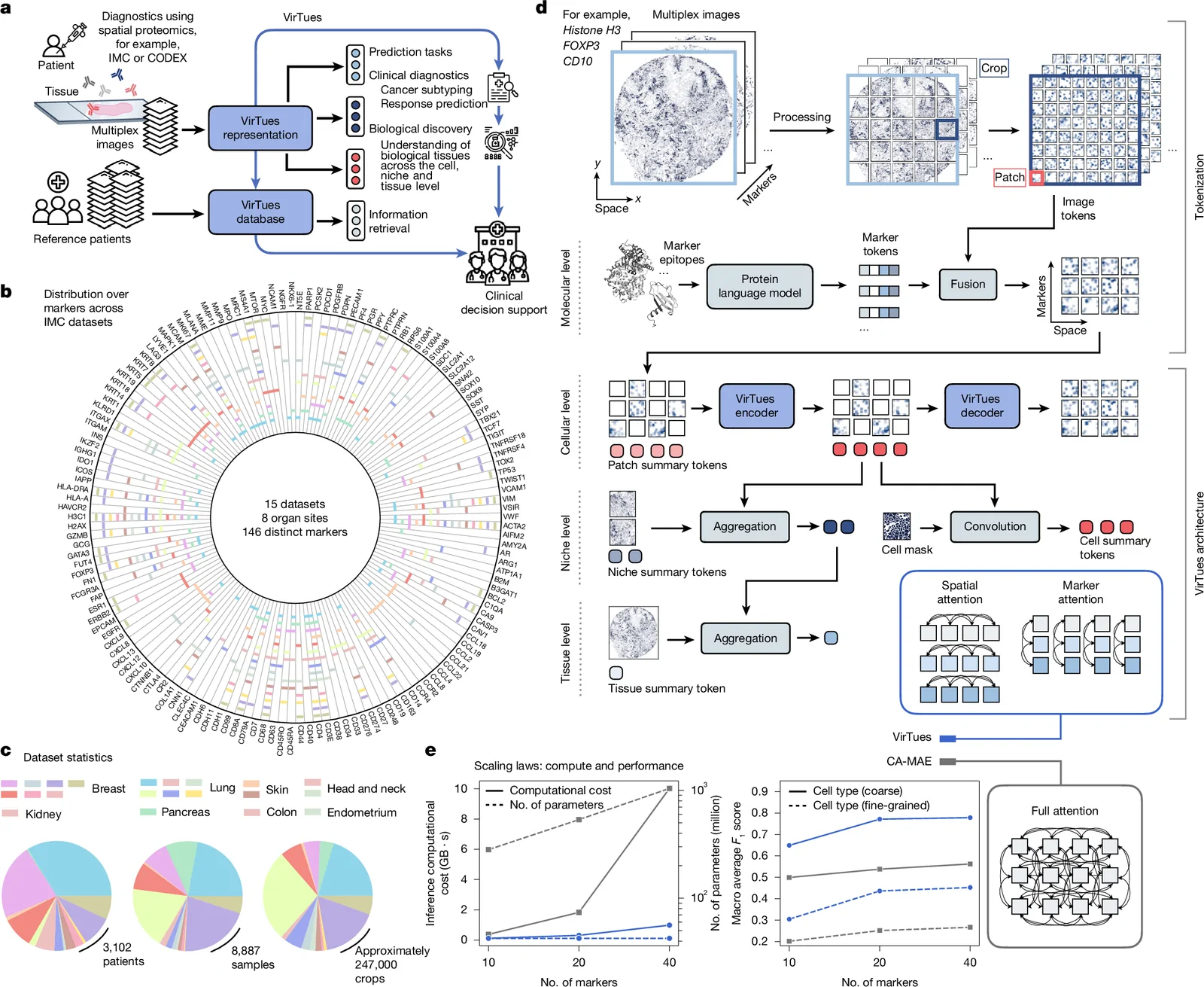
Overview of the VirTues platform. **a**, Flow chart depicting VirTues capabilities. VirTues converts highly multiplexed images of tissue to virtual tissue representations useful for clinical and biological investigations at cell, niche and sample level, including the retrieval of similar tissue samples for clinical decision support. **b**, VirTues is trained and evaluated on 15 IMC datasets with a focus on tumours and their TMEs originating from 8 different organ sites, measuring 146 distinct markers in total. The polar plot depicts used marker panels per dataset. A legend of the dataset colour codes is provided in Extended Data Fig. 1a. **c**, Origins and sizes of datasets in terms of patients, tissue samples and 256 × 256 image crops. **d**, Multiplexed images are processed crop-wise into three-dimensional grids of image tokens, representing patches of each marker at each position. Marker tokens, derived from a PLM, are fused with the respective image tokens using a linear projection and addition. VirTues is a new ViT architecture trained with a masked autoencoding objective. Input tokens are concatenated with patch summary tokens, which are initialized with learnable weights. During inference, VirTues’ encoder processes this set of tokens. The encoded patch summaries are subsequently convolved with the cell segmentation mask into cell summary tokens or aggregated to niche and tissue summary tokens. For training, a random subset of tokens is selected and masked independently for each channel. VirTues’ decoder predicts channel-wise reconstructions receiving as input the encoded, non-masked tokens from the target channel along with all patch summary tokens. VirTues encoder uses sparse attention mechanisms restricting direct token interactions to either positions (marker attention) or channels (spatial attention). **e**, Comparison of computational cost (left) and prediction performance (right) between CA-MAE^31^ and VirTues as a function of the number of markers used.

Capturing this organization requires molecular imaging beyond traditional methods^4^. Spatial proteomics technologies, including multiplexed immunofluorescence and imaging mass cytometry (IMC)^5^, now measure dozens to hundreds of proteins in intact tissue at subcellular resolution, revealing tumour-immune interactions, prognostic spatial niches and mechanisms of therapy resistance across cancer types^6^ (Fig. 1a).

Yet the flexibility that makes these assays powerful also fragments the field: each study uses a customized antibody panel, protocol and platform, so datasets differ in marker identity and number (Fig. 1b), dynamic range and noise. Workflows built for a single assay make it hard to transfer knowledge across cohorts or platforms, reuse annotated references or establish biomarkers that generalize beyond their study of origin^7,8^. Realizing the field’s promise calls for a unified atlas: a harmonized representation in which heterogeneous cohorts, panels and technologies can be analysed jointly. This has been out of reach because conventional encoders assume a fixed panel and cannot relate markers they were not trained on.

Foundation models are suited precisely to this setting: single models trained by self-supervision on large heterogeneous corpora and reused across tasks^9^. They have reshaped natural language processing^10–13^, computer vision^14–17^ and biology^18–20^, and now support oncology diagnosis from pathology and radiology^21–25^. Yet no general-purpose model operates directly on high-plex spatial proteomics across heterogeneous panels: existing encoders require fixed marker panels^26–30^, scale poorly to highly multiplexed data^31,32^ or are evaluated on narrow tasks rather than cross-cohort biomarker discovery^33–35^, and most ignore molecular priors about protein structure and function.

Here we introduce VirTues—a foundation model for spatial proteomics that captures tissue organization across scales. We use the term ‘virtual tissue’ for a computational, spatially resolved representation of measured tissue state that can be queried, compared, reconstructed and reused across tasks, extending the vision of the ‘AI virtual cell’^36^. VirTues combines protein language model (PLM) embeddings with a new factorized transformer whose attention scales to high-dimensional multiplex data while preserving interpretability. By embedding every marker through its protein sequence, it places heterogeneous panels in a shared space, turning fragmented cohorts into a queryable virtual tissue atlas. We assemble the largest open-source collection of spatial proteomics data to date: a core resource of 15 IMC cohorts (3,102 patients, 146 markers) underlying most of our analyses, extended to a multi-technology corpus of 32 cohorts spanning four imaging technologies, including CODEX^37^, Orion^4^ and MIBI^38^, more than 5,100 patients and 239 markers (Fig. 1c). Although developed and evaluated primarily on IMC, VirTues is technology-agnostic.

Across tasks, VirTues outperforms existing methods and shows the strongest cross-technology generalization among the baselines we evaluate. Trained on IMC alone, it remains competitive with multi-technology competitors, indicating robust zero-shot transfer under marker panel and technology shifts. Beyond these performance gains, VirTues spans the spatial proteomics workflow from end to end: it transforms the earliest steps of analysis workflows, including cell segmentation and cell typing, enables virtual experimentation by virtually staining markers that were never measured and pushes the frontier of discovery. In triple-negative breast cancer (TNBC)^2^, the spatial signatures it learns predict anti-programmed death-ligand 1 (PD-L1) chemo-immunotherapy response and stratify disease-free survival in an independent cohort^3^, outperforming previously reported spatial biomarkers^2^ and current clinical stratification schemes. These signatures mark a new generation of spatial biomarkers: rather than single markers or cell-type frequencies, foundation models capture complex multi-cellular signatures spanning the phenotypes and spatial arrangement of many cell populations. More broadly, VirTues offers a reusable computational layer for spatial proteomics and a blueprint for foundation models that couple tissue understanding with clinically actionable biomarker discovery.

## A foundation model for spatial proteomics

A pivotal characteristic of foundation models is their ability to leverage larger and more diverse training datasets to achieve superior performance across diverse downstream tasks. However, current vision models for biological imaging^27,29,31–33^, including Vision Transformers^14^ (ViTs), face several limitations when applied to multiplexed data (Fig. 1e) that constrain their scalability to large, heterogeneous collections. First, their computational complexity scales quadratically with spatial dimensions and channel number, making them impractical for high-dimensional spatial data. Second, their token-based representations treat all channels equally, failing to capture important marker-specific information and to discount redundant information from different markers. Third, they lack explicit mechanisms for integrating datasets with differing marker panels or for incorporating previous knowledge about the probed molecules (for example, protein sequence and structure).

We designed VirTues to overcome these challenges as a purpose-built ViT model for multiplexed imaging of proteins and mRNAs. By disentangling the transformer’s attention^39,40^ into marker and spatial components, VirTues can be trained on images with dozens of channels (Fig. 1e) and separately learns the spatial arrangement and cellular composition that shape a tissue’s molecular profile together with the interrelations between protein and RNA markers. Another critical innovation is a new tokenization scheme for spatial biology: by combining PLM embeddings^18^ with spatially patched channel information and learnable patch summary tokens, we enable flexible processing of variable marker combinations while incorporating the biological meaning and subcellular spatial distribution of markers (Fig. 1d; Methods). This tokenization and multi-scale design^36,41^ thus allows VirTues to incorporate markers not seen during training and to integrate heterogeneous datasets measured with different marker combinations (Fig. 1b). Such multiplex-aware tokenization is critical: modality-agnostic designs neither scale nor capitalize on additional markers, whereas VirTues improves consistently with marker depth, especially for the first 20 markers (Fig. 1e).

**Fig. 2:**
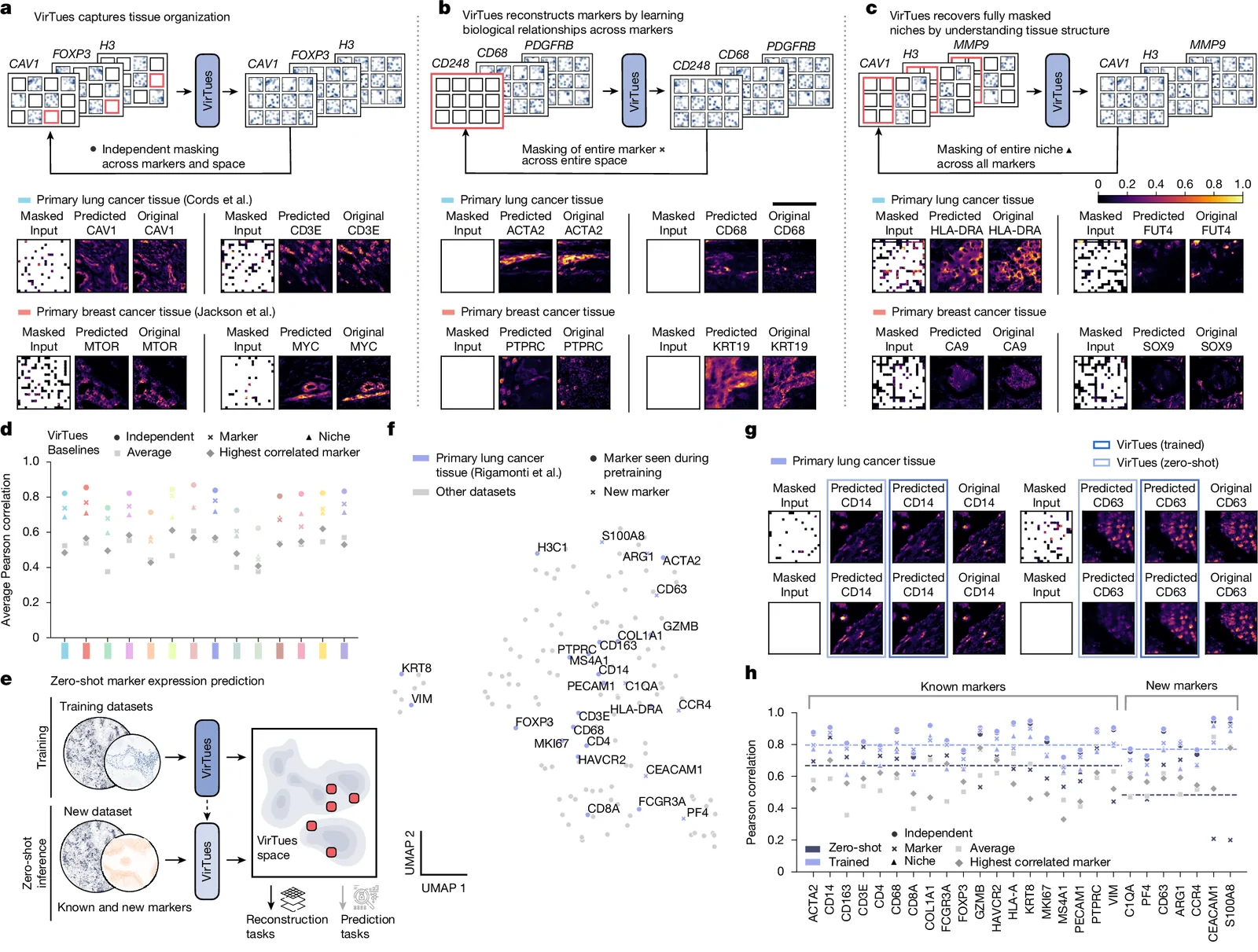
VirTues learns tissue architecture and marker relationships. **a**–**c**, Masking strategies and reconstruction examples in primary lung cancer^42^ and breast cancer^43^ tissue. Visualized images are rescaled to [0, 1] by inverting preprocessing and dividing by the 99th percentile. **a**, Independent masking. Tokens of each channel are masked independently with a channel-wise random masking ratio of 60–100%. **b**, Marker masking. All tokens of one randomly chosen marker are masked, other channels remain unmasked. Each row shows inpainting of different channels from the same tissue sample. Scale bar, 100 µm. **c**, Niche masking. A subset of spatial positions is chosen and all tokens across markers at these positions are masked. Each row shows niche reconstructions of different channels from the same sample. **d**, Per-dataset reconstruction performance (Pearson correlation, averaged across markers) for independent (circle), marker (cross) and niche (triangle) masking. Baselines predict the mean visible pixel intensity per channel (light grey squares) or infer each marker from its most correlated partner (dark grey diamonds). Performance is assessed on masked tokens only. Dataset colour codes follow Extended Data Fig. 1a. **e**, Overview of zero-shot marker expression prediction. During training (top), VirTues encodes multiplexed tissue images into a shared virtual tissues space. At inference (bottom), a new dataset with possibly previously unseen markers is encoded by the same model into this space without retraining. Resulting representations support both reconstruction and prediction tasks. **f**, UMAP of ESM-2-derived marker token embeddings. Markers in the panel of the dataset from Rigamonti et al. ^8^ are shown in blue, and others in light grey. Circles denote markers seen during pretraining (excluding ref. ^8^); crosses denote markers unique to that dataset. **g**, Zero-shot reconstructions of CD14 (seen) and CD63 (unseen) from ref. ^8^, compared with reconstructions from a model also pretrained on this dataset. **h**, Marker-wise reconstruction performance on primary lung cancer tissue^8^ in the zero-shot versus non-zero-shot setting, grouped by seen (left) and unseen (right) markers. Dashed lines indicate per-group averages under marker masking.

**Fig. 3:**
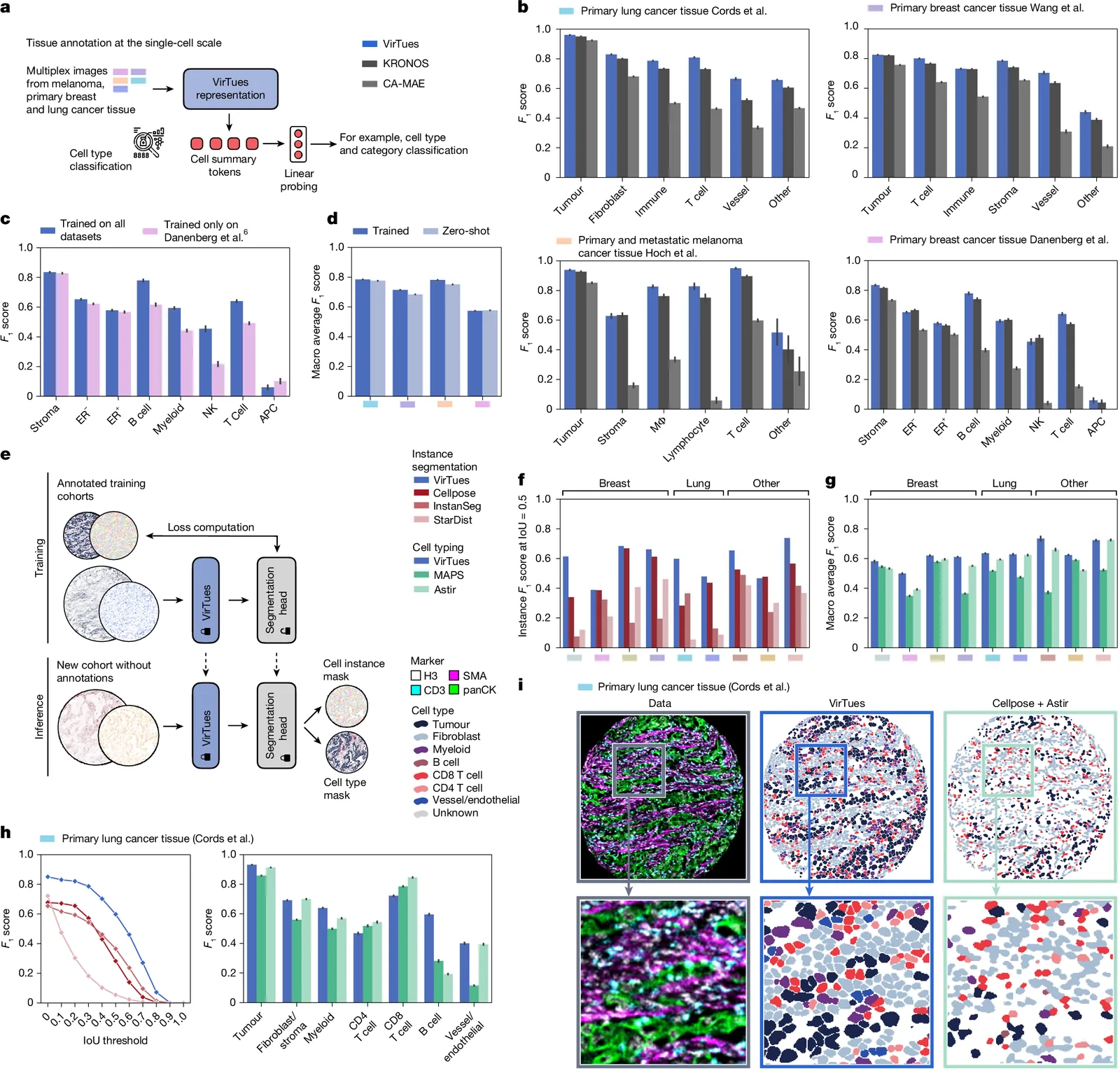
VirTues segments and annotates tissues at the single-cell scale. **a**, Cell types are classified with logistic regression using individual cell summary tokens. **b**, *F*_1_ scores for cell type classification for VirTues, KRONOS^35^ and CA-MAE^31^ on Cords et al.^42^, Wang et al.^2^, Hoch et al.^48^ and Danenberg et al.^6^. **c**, Comparison of *F*_1_ scores for cell type classifications between VirTues trained on all pretraining datasets and VirTues trained only on dataset from Danenberg et al.^6^. **d**, Comparison of cell type classification performance in the zero-shot and the non-zero-shot setting measured by macro-averaged *F*_1_ score. Results are shown for datasets from Cords et al.^42^, Wang et al.^2^, Hoch et al.^48^, Danenberg et al.^6^. **e**, VirTues enables cross-cohort cell segmentation and typing. Using harmonized cell type annotations from several datasets, we trained a segmentation head on VirTues’ patch-level representations to jointly predict cell instance masks and semantic cell type masks. The resulting model generalizes to previously unseen datasets, enabling simultaneous segmentation and annotation of tissue images without target-specific retraining or fine-tuning. **f**, Instance segmentation performance, reported as *F*_1_ score at IoU = 0.5, for VirTues, Cellpose^49^, InstanSeg^50^ and StarDist^51^ on datasets excluded from corresponding segmentation model training. **g**, Cell type classification performance, reported as macro-averaged *F*_1_ score, for VirTues, MAPS^54^ and Astir^55^ on datasets excluded from corresponding segmentation model training. A legend of the dataset colour codes used in **f** and **g** is provided in Extended Data Fig. 1a. **h**, Comparison of instance segmentation *F*_1_ scores across varying IoU thresholds against segmentation baselines (left), and comparison of per-cell type *F*_1_ scores with cell typing baselines (right). **i**, Representative cell type mask generated by VirTues. We compare against the underlying multiplexed imaging data (left) and against the prediction obtained by combining Cellpose^49^ instance segmentation with Astir^55^ cell type assignment (right). Error bars (**b**–**d**,**g**,**h**), 95% confidence intervals estimated using *n* = 1,000 bootstrap resamples and centred around the bootstrap mean.

**Fig. 4:**
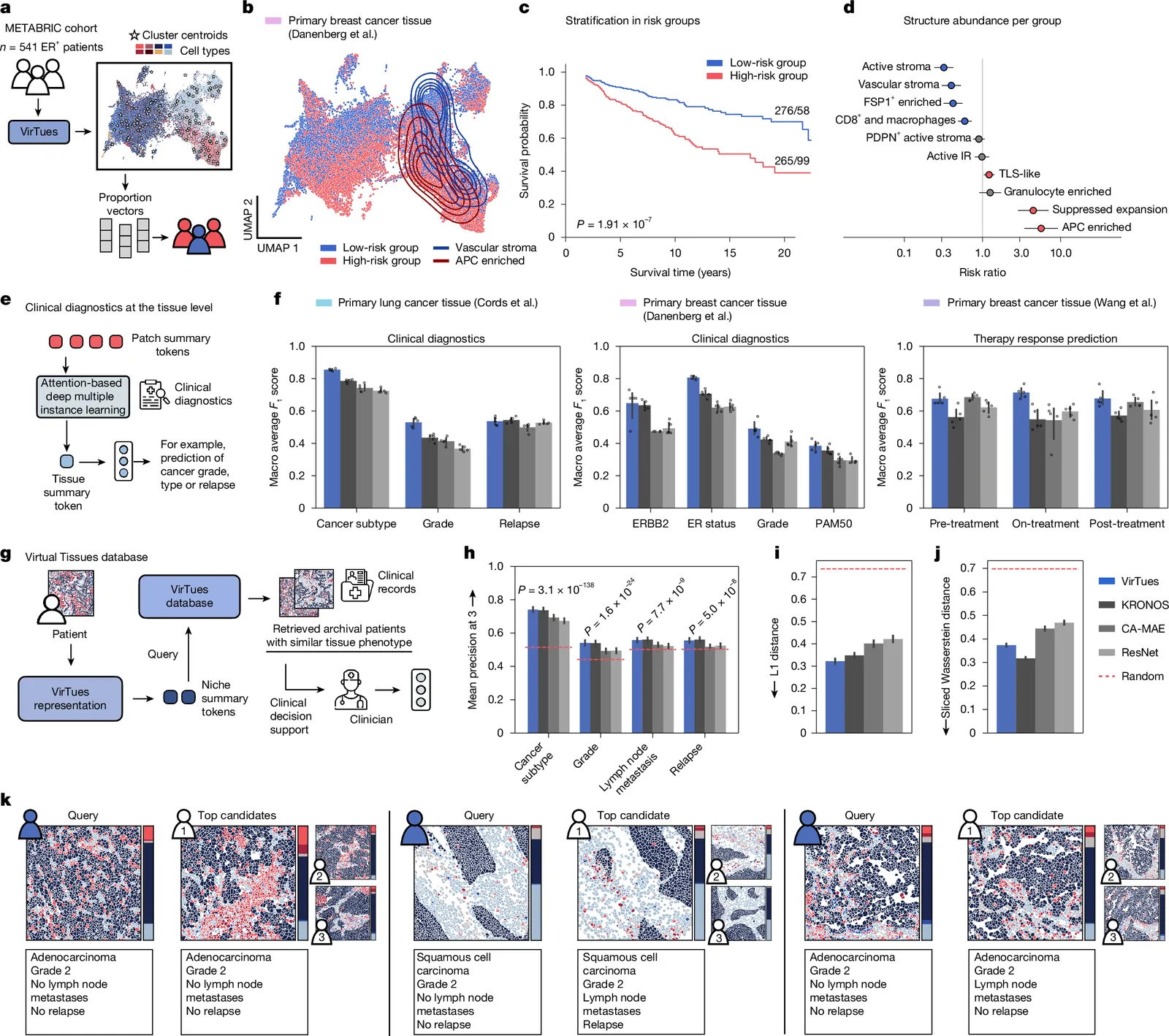
Clinical applications of VirTues: risk stratification, diagnostic predictions, and tissue retrieval by similarity. **a**–**c**, TME-based patient risk stratification. **a**, Cell-level VirTues representations of oestrogen receptor positive (ER^+^) METABRIC patients^6,57^ are clustered with *k*-means. Per-patient cluster proportion vectors are then re-clustered to define risk groups. **b**, UMAP of cell summary tokens coloured by risk level. KDE overlays show distributions of cells from vascular stroma and APC-enriched TME structures, previously linked to decreased and increased hazard ratios^6^. **c**, Kaplan–Meier survival curves for high- and low-risk groups with log-rank *P* value. **d**, Risk ratios of each TME structure in the high-risk group with 95% Wald confidence intervals (*n* = 541 patients). **e**, Clinical patient features predicted from patch summary tokens using ABMIL. **f**, Macro-averaged *F*_1_ scores for tissue-level prediction on datasets from refs. ^2,6,46^. Error bars, 95% confidence intervals (*n* = 5 training runs). For response prediction on data from ref. ^2^, performance is reported separately for biopsies collected before, during or after treatment. **g**, VirTues retrieves similar patient cases from a database of niche summary token representations using optimal transport-based similarity. **h**–**j**, Retrieval statistics on Cords et al.^42^ comparing VirTues, KRONOS^35^, CA-MAE^31^ and ResNet^26^. Red dashed lines, random retrieval; error bars, 95% confidence intervals (*n* = 1,000 bootstrap resamples). **h**, Top-three mean precision for cancer subtype, grade, lymph node metastasis and relapse retrieval. *P* values from two-sided McNemar tests compare VirTues against random retrieval. **i**, Cell type composition similarity between query and closest match (L1 distance between cell type proportion vectors). **j**, Molecular composition similarity between query and closest match (sliced Wasserstein distance between marker intensity vectors). **k**, Exemplary retrieval results on Cords et al.^42^: each column shows a query tissue and its three closest matches as colour-coded cell type masks with proportional composition colour bars.

**Fig. 5:**
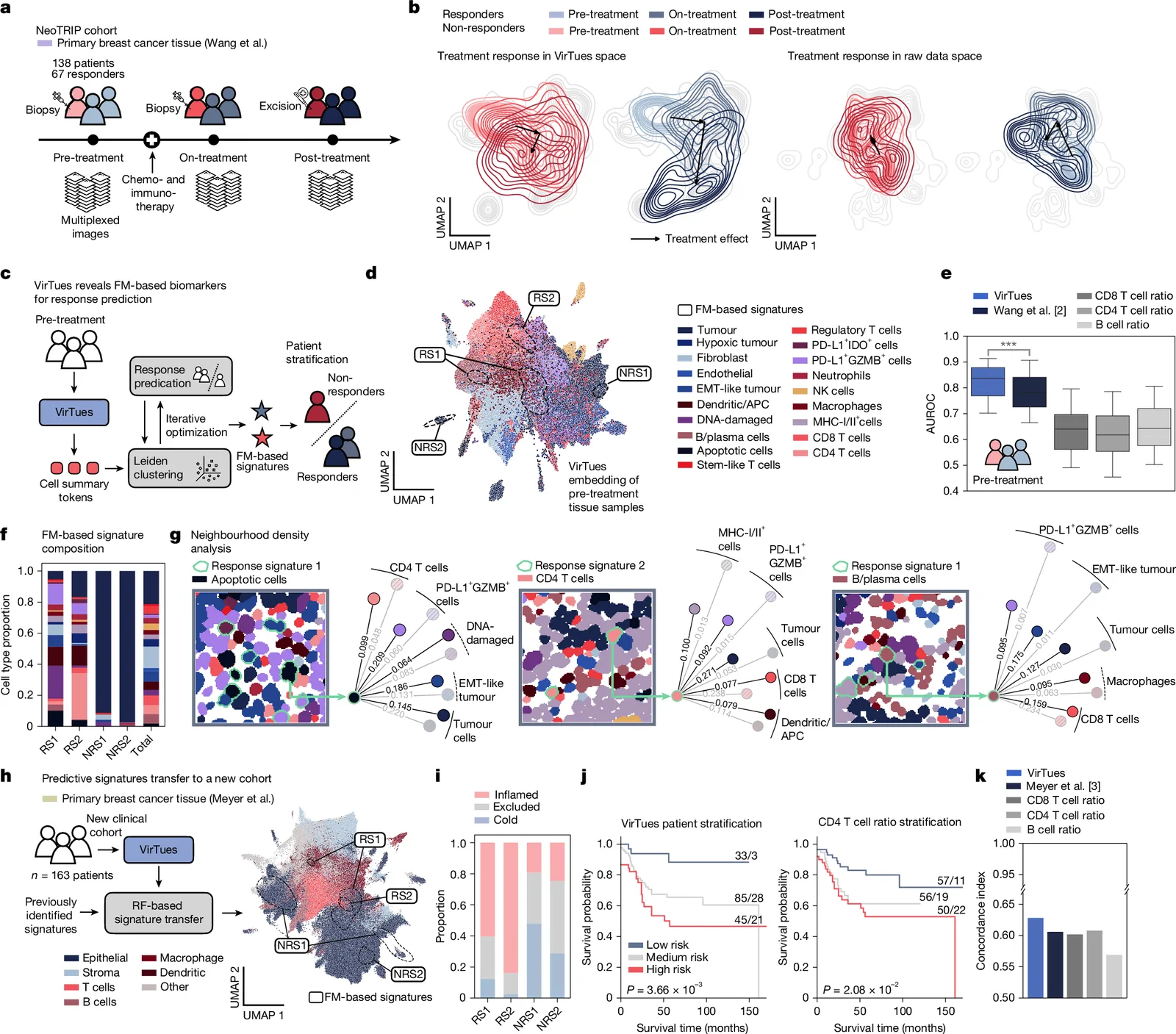
Discovery of VirTues biomarkers for treatment response and survival prediction. **a**, NeoTRIP cohort^2,59^ of 138 patients with breast cancer received chemo-immunotherapy (67 complete pathological responders), sampled before, during and after treatment. **b**, Virtual tissue (left) and raw mean marker intensity (right) distributions across treatment stages for non-responders and responders (KDE plots over UMAP). The responder signal is detectable only in foundation model-based representations. Black arrows connect the barycenters of consecutive biopsy phases in the UMAP embedding. **c**, Pre-treatment cell-level virtual tissue representations are iteratively clustered by Leiden at several resolutions. The most predictive clusters serve as biomarkers for joint response prediction. **d**, UMAP of pre-treatment cell-level representations coloured by cell type. Dashed contours indicate response signature (RS) and non-response signature (NRS) distributions. **e**, Cross-validated AUROC for response prediction on pre-treatment samples versus the spatial predictor of Wang et al.^2^ and immune-ratio baselines (tumour-to-CD4 T cell, CD8 T cell and B cell ratios)^45,46^. Boxes show quartiles; whiskers span 5th–95th percentiles; grey brackets significance versus second-best method (two-sided *t*-test, *P* = 2.43 × 10^−4^, *n* = 100 splits). **f**, Cell type composition of signatures and overall proportions. **g**, Neighbourhood density analysis for apoptotic cells (RS1), CD4 T cells (RS2), and B/plasma cells (RS1). Tree diagrams compare neighbour type frequencies inside (black arrows) versus outside (grey arrows) each signature. Solid arcs indicate significant differences (*P* < 0.05, two-sided proportions *z*-test). **h**, Signatures transferred to a new breast cancer cohort^3^ using random forest on virtual tissue representations. **i**, Distribution of immune-inflamed, excluded and cold cell labels within each transferred signature. **j**, Risk stratification based on presence of foundation model-based biomarkers (left) and tumour-to-CD4 T cell ratio (right) across patients with Kaplan–Meier curves and log-rank *P* values. **k**, Corresponding concordance index of stratification with VirTues’ biomarkers versus biomarkers from Meyer et al.^3^ and baselines. FM, foundation model.

**Extended Data Fig. 1:**
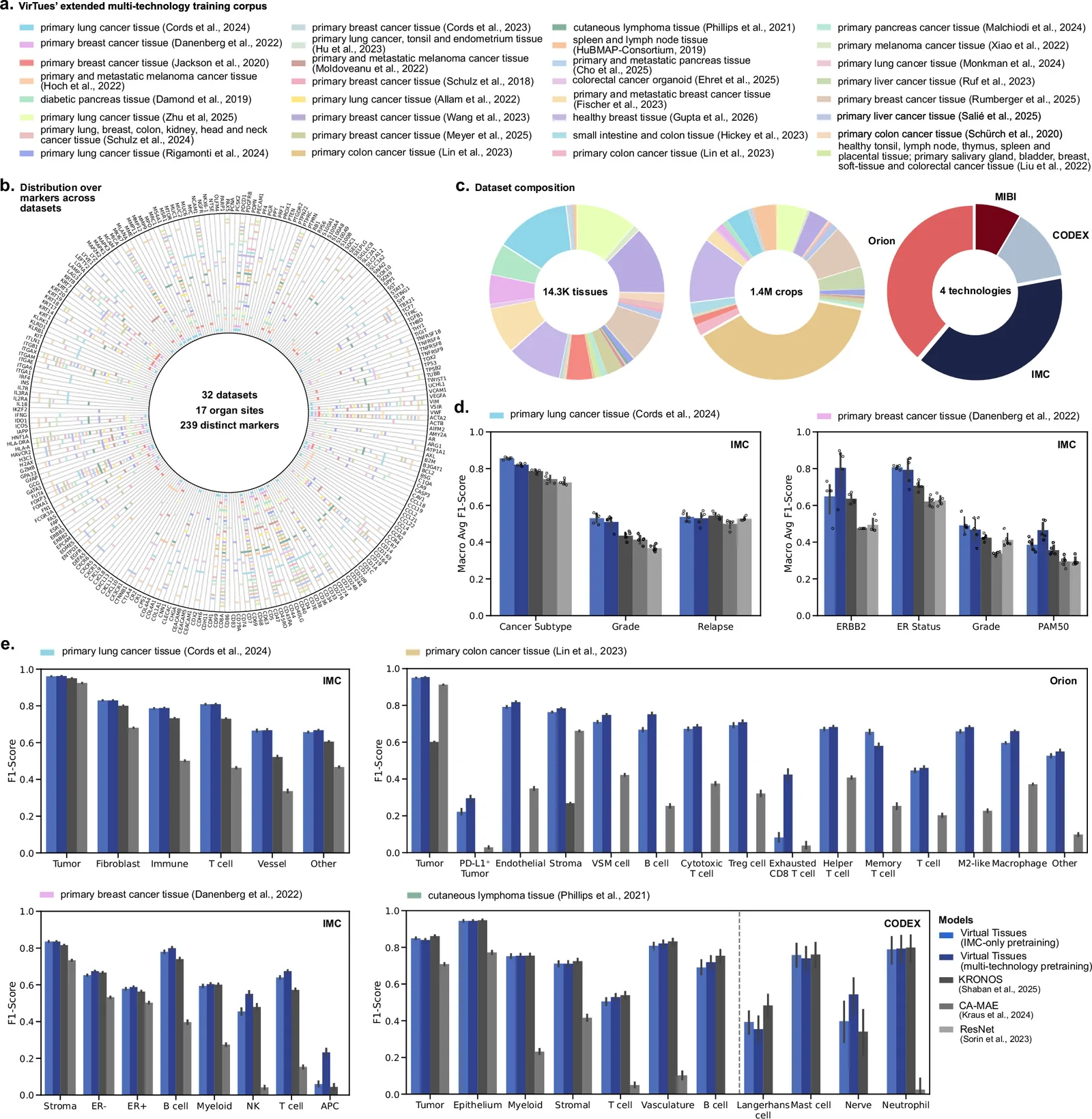
Overview and benchmark of VirTues on the extended multi-technology data corpus. **a**, List of the 32 datasets included in the corpus and their assigned colors, spanning multiple spatial proteomics technologies, including MIBI, Orion, CODEX, and IMC. **b**, Distribution of measured markers across datasets. **c**, Number of tissues per dataset (left), image crops per dataset (center), and image crops per imaging technology (right) within the extended data corpus. **d-e**, Comparison of downstream performance between VirTues (IMC-only), VirTues (multi-technology), and baselines. **d**, Macro-averaged *F*_1_ scores for tissue-level prediction on the IMC datasets from Cords et al.^42^ and Danenberg et al.^6^. Error bars indicate 95% confidence intervals estimated from n=5 training runs. **e**, *F*_1_ scores for cell type classification on IMC datasets from Danenberg et al.^6^ and Cords et al.^42^, the CODEX dataset from Phillips et al.^44^, and the Orion dataset from Lin et al.^4^. For Phillips et al.^44^, a dashed gray line separates rare cell types, defined as classes with fewer than 100 test cells (out of n=27739 total test cells). For the IMC datasets, we report results of a custom instance of KRONOS trained on our IMC data collection. For CODEX and Orion datasets, we utilize the model released by Shaban et al.^35^. Error bars indicate 95% confidence intervals estimated using n=1000 bootstrap resamples.

We train and evaluate VirTues on a collection of 15 IMC datasets spanning 8 organ sites (Fig. 1c and Supplementary Table 1) and, to assess cross-technology generalization, extend this corpus with public CODEX, Orion and MIBI studies, resulting in a dataset encompassing 32 clinical cohorts from 17 different tissue origins (Extended Data Fig. 1a–c and Supplementary Table 2). Built on the masked autoencoder (MAE) framework^15^, VirTues comprises an Encoder and Decoder trained jointly and unsupervised to reconstruct partially masked marker-space tensors (Fig. 2; Methods). By recovering missing information it captures patterns across subcellular, cellular and multi-cellular scales. From this representation, VirTues generates patch, cell, niche and tissue summary tokens (Fig. 1d)—a hierarchical readout that supports tasks from cell annotation and marker inpainting to clinical prediction and biological discovery (Figs. 3–5).

## Reconstructing masked tissue and markers

To probe VirTues’ understanding of tissue architecture, we tested its ability to reconstruct tissues under three masking strategies of increasing difficulty: ‘independent masking’, where patches are masked at random locations independently per marker (Fig. 2a); ‘marker masking’, where all patches of a single marker are masked (Fig. 2b) and ‘niche masking’, where patches across all markers are masked at random locations (Fig. 2c).

Under independent masking, VirTues reconstructs masked regions with high Pearson correlation (average *r*_independent_ = 0.800 ± 0.130) across markers and datasets (Fig. 2d; marker-wise performance in Supplementary Figs. 1 and 2), far exceeding a mean-intensity baseline (Δ*r*_independent_ = 0.295). Qualitative examples, such as CAV1 and CD3E in lung cancer tissue^42^ and MTOR and MYC in breast cancer samples^43^, show that the reconstructions preserve both spatial distribution and intensity patterns (further markers across all datasets in Supplementary Figs. 3–5). Under the harder marker masking, where an entire channel is hidden, VirTues accurately recovers the masked marker’s expression (average *r*_masking_ = 0.700 ± 0.173; Fig. 2d), for example, ACTA2 and CD68 in lung cancer^42^ and PTPRC and KRT19 in breast cancer tissue^43^ (further markers in Supplementary Figs. 6–8), surpassing a baseline that predicts the most correlated marker (Δ*r*_masking_ = 0.195) and demonstrating the model’s ability to learn and integrate complex marker interactions. Because, under marker masking, the VirTues Decoder receives only the encoded patch summary tokens as input, its ability to reconstruct the missing marker validates that these tokens capture a robust representation of the molecular tissue architecture. Under niche masking, where whole regions are occluded across all markers, VirTues reconstructs complex tissue architectures (average *r*_niche_ = 0.668 ± 0.132; Fig. 2d), recovering marker patterns such as HLA-DRA and FUT4 in lung cancer^42^ and CA9 and SOX9 in breast cancer tissue^43^ (further markers in Supplementary Figs. 9–11). The recovery of spatially coherent macro-structures shows that the model captures global tissue architecture rather than relying solely on local pixel information.

Finally, we assessed zero-shot reconstruction on an independent lung cancer dataset^8^ withheld entirely from a separate training run (Fig. 2e), which contained both markers present in training and new markers not seen during training (Fig. 2f). VirTues produced visually accurate reconstructions for both (for example, CD14 and CD63; Fig. 2g and Supplementary Figs. 12 and 13), with marker-wise performance varying by marker and strategy (Fig. 2h and Supplementary Fig. 14). For known markers (previously seen in training cohorts), zero-shot reconstructions achieved high correlations (average *r* = 0.667) despite a reduction relative to in-domain evaluation (average *r *= 0.797). Notably, this drop occurred only when entire channels were masked, not under independent or niche masking (Δ*r *= 0.016 and Δ*r *= −0.002, respectively). For new markers present only in the zero-shot dataset, the drop was more striking but again concentrated under marker masking. Moreover, zero-shot performance under independent masking exceeded that under niche masking, indicating that, even for new markers, VirTues leverages inter-marker relationships, not just local spatial patterns. We further analysed when zero-shot reconstruction is expected to succeed versus fail, and observed a clear trend: it performs best when the target marker has close analogues among proteins seen during pretraining, corresponding to dense local support in the pretrained protein representation space (Supplementary Fig. 15).

Virtual stains produced by marker masking and reconstruction across whole tissues further enabled downstream phenotyping: sequentially masking and imputing all B cell and then all T cell markers preserved cell type classification (Supplementary Fig. 16), confirming the biological validity of the reconstructed signals.

## VirTues phenotypes cells across cohorts

VirTues’ unified framework of patch, cell, niche and tissue summary tokens (Fig. 1d) supports analysis across biological scales, beginning with cell type prediction at single-cell resolution—a prerequisite for characterizing the TME and informing therapeutic decisions^2,44,45^.

We first evaluated within-cohort cell typing by linear probing (logistic regression) on VirTues’ cell summary tokens (Fig. 3a)—a standard readout of encoder representation quality^46^, comparing against two baselines, KRONOS^35^ and channel-agnostic MAE (CA-MAE)^31^. Across lung, breast and melanoma datasets (Fig. 3b), VirTues consistently led in macro-averaged *F*_1_, with average relative improvements of +5.81% over KRONOS^35^ and +65.79% over CA-MAE^31^, and maintained strong performance on fine-grained, highly imbalanced phenotypes in Cords et al.^42^ and Wang et al.^2,42^ (Supplementary Fig. 17). Gains held even for exquisitely rare populations such as vessel (<1.6%) and T cells (<6.3%) in Cords et al.^42^ and natural killer (<1.8%) and B cells (<4.9%) in Danenberg et al.^6^ (support distributions in Supplementary Figs. 18 and 19).

Trained without any architectural change on several technologies, VirTues (multi-technology) reached state-of-the-art cell typing on both the cutaneous lymphoma CODEX^47^ and colorectal cancer Orion^4^ datasets (macro-*F*_1_ 0.705 and 0.652), outperforming KRONOS^35^ and CA-MAE^31^ on Orion by a large margin (Extended Data Fig. 1; error analysis in Supplementary Fig. 20). A critical property of foundation models is that they improve as the size and diversity of their training data increase. VirTues showed this behaviour: on the dataset of Danenberg et al.^6^, a model trained on all available datasets surpassed one trained on data from Danenberg et al.^6^ alone across nearly all classes (Fig. 3c), with the largest gains for rare immune populations (B cells +26.4%, myeloids +34.3%, natural killer cells +109.7%, T cells +30.2%). These results demonstrate effective cross-dataset synergy: VirTues leverages representations learned from diverse, independent cohorts to surpass a model trained on the data from Danenberg et al.^6^ alone.

Finally, VirTues generalized in a strict zero-shot setting: across the four benchmarking cohorts^2,6,42,48^, withholding each evaluation cohort entirely during training left macro-*F*_1_ nearly unchanged relative to in-domain training (differences ≤0.03; Fig. 3d). It also transferred to spatial proteomics technologies not encountered during training, achieving zero-shot macro-*F*_1_ of 0.691 on CODEX^47^ and 0.607 on Orion^4^, closely approaching the multi-technology instance despite never encountering either technology during training, and exceeding KRONOS^35^, which performed substantially worse even though it was trained on proprietary Orion data (Extended Data Fig. 1e).

## Zero-shot segmentation across cohorts

The preceding experiments relied on provided segmentation masks and within-dataset labels. Because VirTues maps diverse datasets into a shared space of spatially resolved patch representations, it also enables cross-cohort segmentation and cell typing, which is especially valuable for annotating new, unlabelled datasets. We harmonized cell type annotations across datasets and, with the provided masks, trained segmentation modules on top of VirTues that jointly predict instance masks and semantic cell type masks (Fig. 3e; Methods). To assess cross-cohort generalization, each module was trained on all but the target dataset, which was withheld entirely, making evaluation zero-shot with no target-specific fine-tuning.

**Extended Data Fig. 2:**
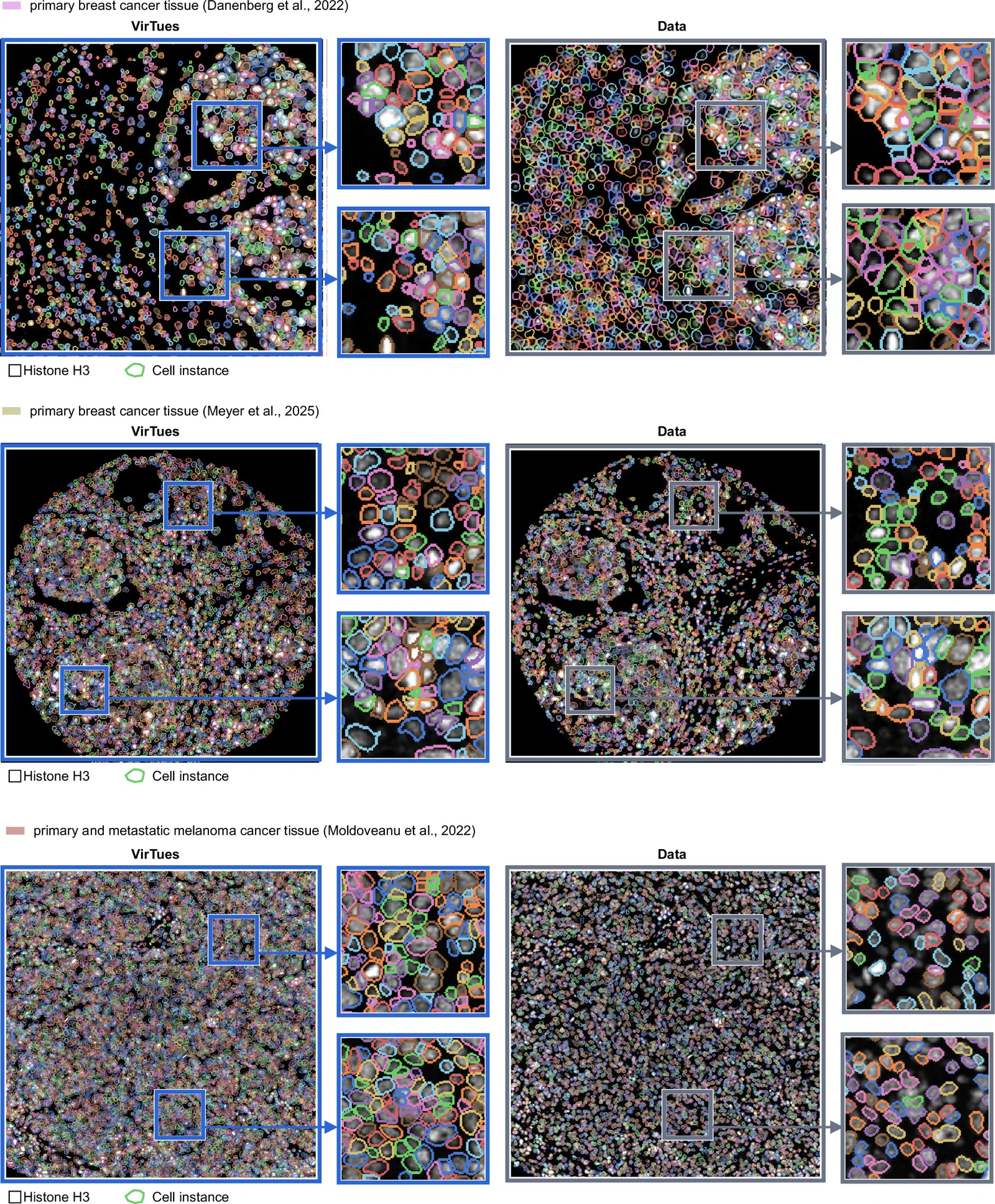
Examples of cell instance segmentation masks for tissues from the datasets of Danenberg et al.^6^, Meyer et al.^3^, and Moldoveanu et al.^56^. The left column shows instance segmentation masks predicted by segmentation heads trained on top of VirTues’ representations. These predictions are generated out of distribution, i.e., each segmentation head was evaluated on a dataset excluded from its training. The right column shows the corresponding reference segmentation masks provided by the original studies, which serve as ground truth for evaluation.

**Extended Data Fig. 3:**
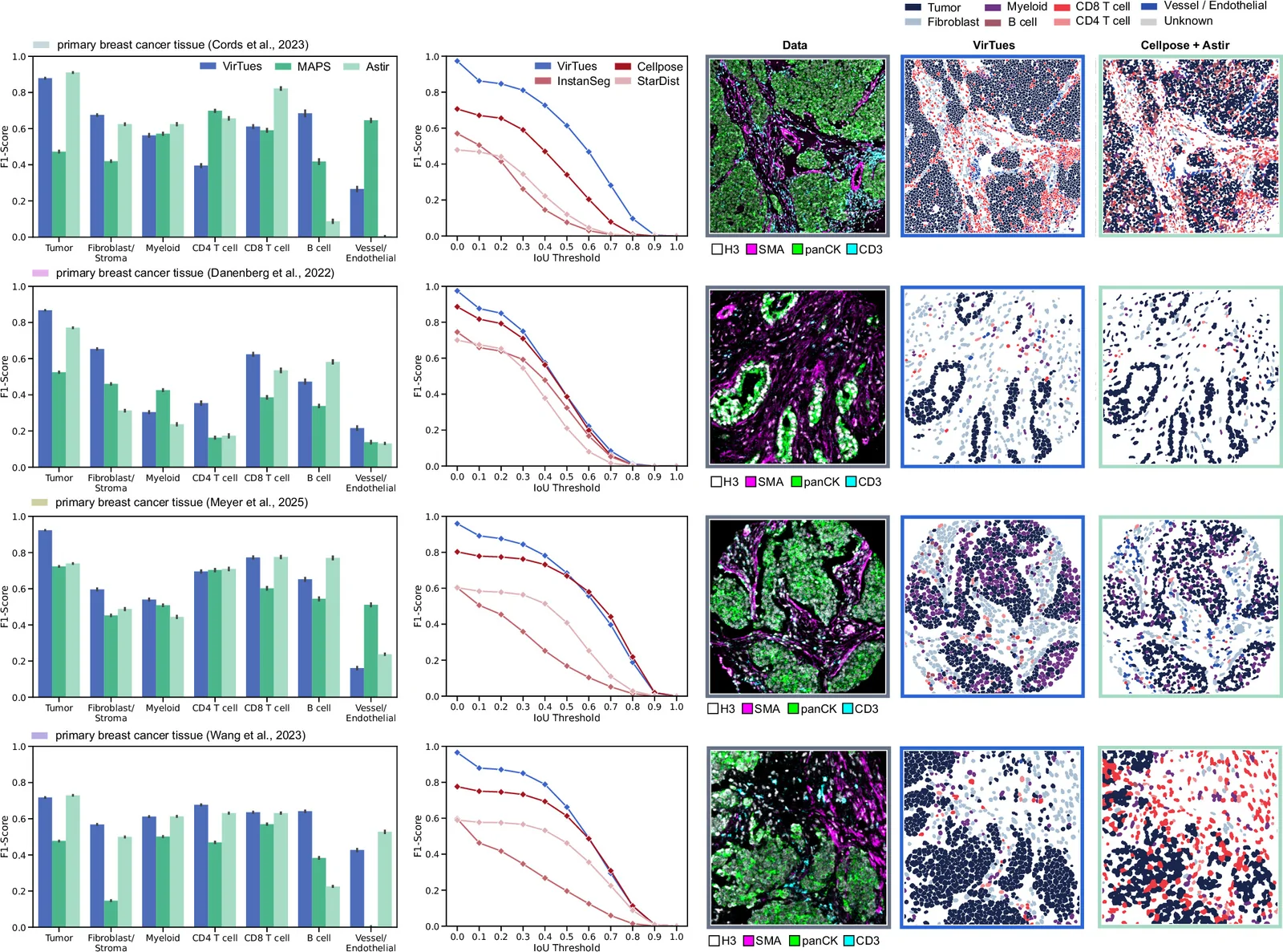
Detailed cross-cohort cell type and cell instance segmentation results for the breast cancer datasets of Cords et al.^52^, Danenberg et al.^6^, Meyer et al.^3^, and Wang et al.^2^. Each row corresponds to one dataset and shows, from left to right: (i) a comparison of cell type prediction *F*_1_ scores between VirTues, MAPS^54^, and Astir^55^; (ii) a comparison of cell instance prediction *F*_1_ scores across different intersection-over-union thresholds between VirTues, Cellpose^49^, InstanSeg^50^, and StarDist^51^; and (iii) a qualitative example consisting of the underlying multiplexed data, the cell type segmentation masks predicted by VirTues, and the cell type segmentation masks obtained by combining Cellpose’s^49^ instance segmentation with Astir’s^55^ cell type predictions. Error bars indicate 95% confidence intervals estimated using n=1000 bootstrap resamples.

**Extended Data Fig. 4:**
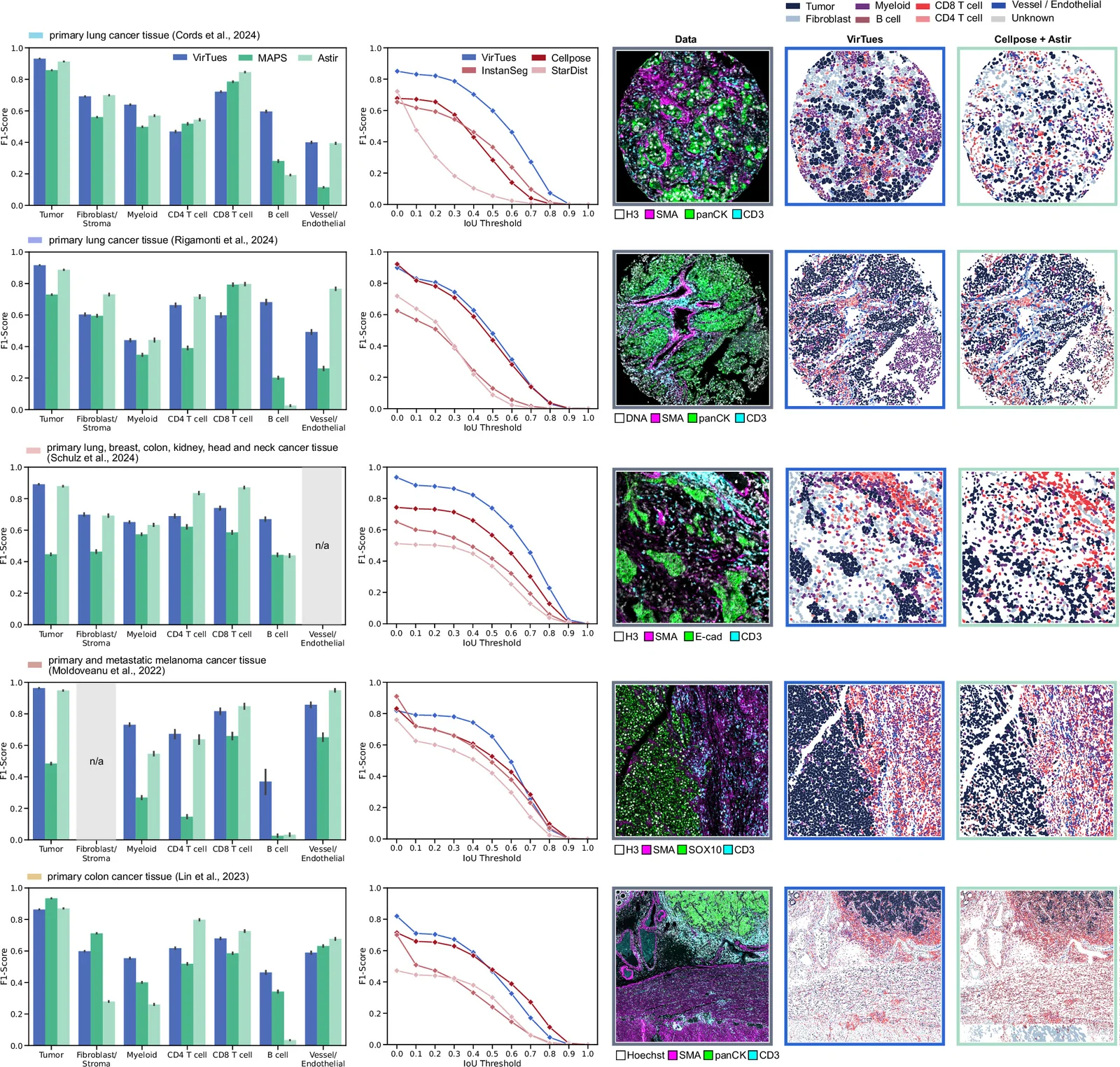
Detailed cross-cohort cell type and cell instance segmentation results for the datasets of Cords et al.^42^, Rigamonti et al.^8^, Schulz et al.^53^, Moldoveanu et al.^56^ and Lin et al.^4^. Each row corresponds to one dataset and shows, from left to right: (i) a comparison of cell type prediction *F*_1_ scores between VirTues, MAPS^54^, and Astir^55^; (ii) a comparison of cell instance prediction *F*_1_ scores across different intersection-over-union thresholds between VirTues, Cellpose^49^, InstanSeg^50^, and StarDist^51^; and (iii) a qualitative example consisting of the underlying multiplexed data, the cell type segmentation masks predicted by VirTues, and the cell type segmentation masks obtained by combining Cellpose’s^49^ instance segmentation with Astir’s^55^ cell type predictions. Error bars indicate 95% confidence intervals estimated using n=1000 bootstrap resamples. Grey panels indicate harmonized cell types absent from the corresponding dataset.

**Extended Data Fig. 5:**
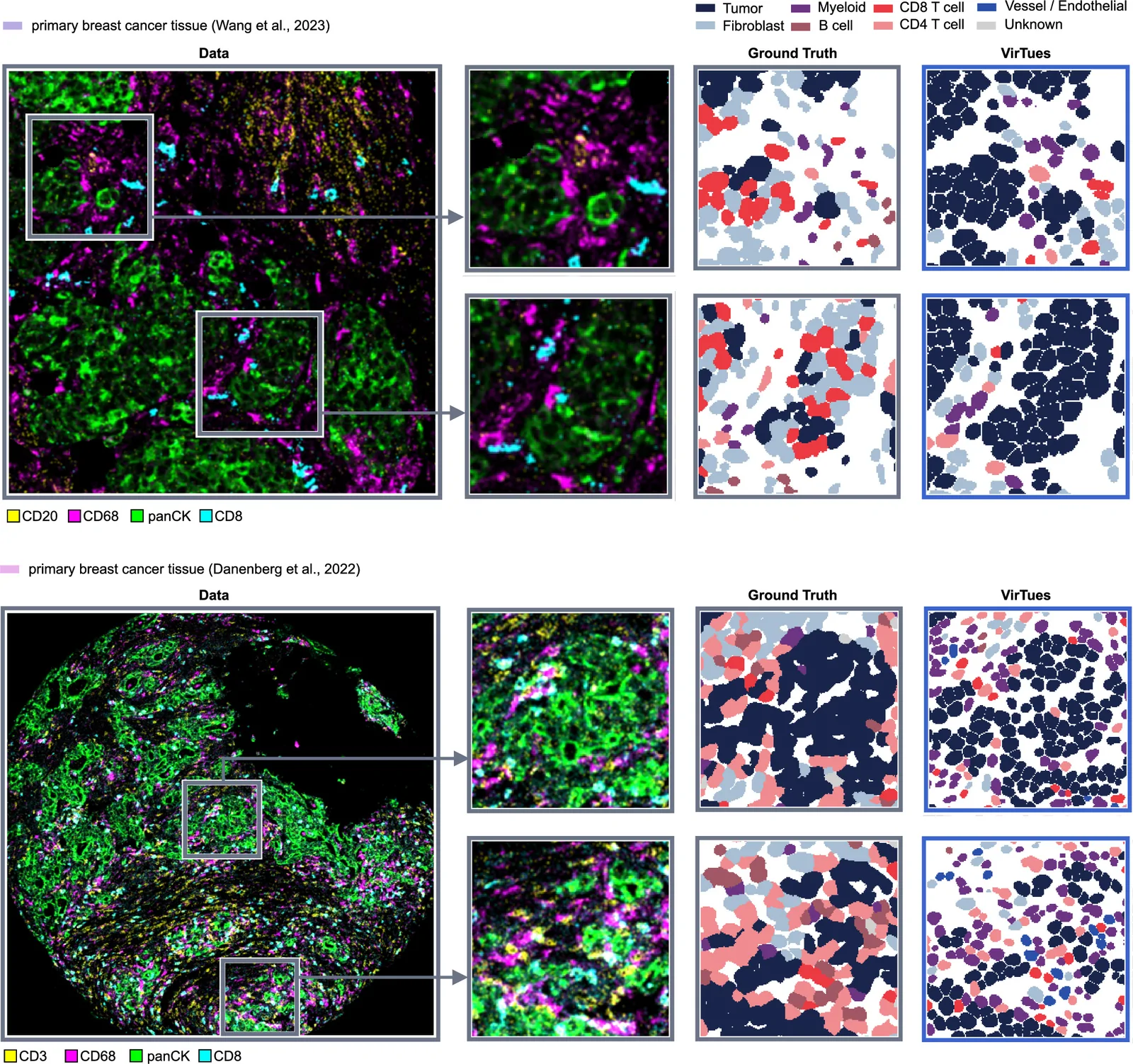
Examples of semantic cell type masks for tissues from the datasets of Wang et al.^2^ and Danenberg et al.^6^. For each tissue sample, panels show from left to right: (i) the underlying imaging data for the full tissue section, projected to RGB using four selected channels; (ii) zoomed-in views of two representative regions; (iii) ground-truth cell type annotations provided by the original studies; and (iv) zero-shot cell type predictions generated by VirTues.

Across datasets, VirTues performed strongly on both instance segmentation (Fig. 3f) and cell-type prediction (Fig. 3g). At an intersection-over-union (IoU) threshold of 0.5, it outperformed the established models Cellpose^49^, InstanSeg^50^ and StarDist^51^ on 8 of 9 datasets, often by a wide margin over the second-best baseline Cellpose^49^ (for example, +80.30% on Cords et al.^52^, +30.45% on Schulz et al.^53^). Qualitatively, its predicted masks were in some cases more accurate than the provided ground truth, suggesting annotation variability contributes to the residual gap (Extended Data Fig. 2). Similar improvements held across IoU thresholds (Fig. 3h, and Extended Data Figs. 3 and 4). For cell typing, VirTues’ macro-*F*_1_ exceeded the specialized tools MAPS^54^ and Astir^55^ on all but one dataset (gains up to +27.31% on data from Danenberg et al.^6^ and +11.31% on Moldoveanu et al.^56^) and in several examples its predicted cell types matched the expression of the markers that are used commonly to identify those cell types (for example, panCK for epithelial tumour cells) more closely than the reference annotations (Extended Data Fig. 5). Comparing class-wise *F*_1_ scores, VirTues showed the most consistent performance across cell types, whereas MAPS^54^ and Astir^55^ exhibited substantially higher variance (Fig. 3h and Extended Data Figs. 3 and 4). Together, these results suggest that VirTues learns robust representations of cell morphology and phenotype across diverse datasets and marker panels, enabling strong generalization to new cohorts.

## TME risk groups stratify breast cancer

A central goal of spatial biology is to find cellular and multi-cellular features that stratify patients by clinical risk^1^. We applied the fully trained VirTues to the METABRIC breast cancer cohort^57^ analysed in Danenberg et al.^6^, restricted as there to oestrogen receptor-positive patients (*n* = 541). For each patient we embedded tissue biopsies with VirTues, tiled the resulting cell tokens into 120 phenotypic states by *k*-means and distilled each tumour into a phenotype-composition fingerprint (Fig. 4a). Clustering these fingerprints stratified patients into two risk groups. In uniform manifold approximation and projection (UMAP) visualizations, these groups showed distinct distributions of cell-level representations (Fig. 4b). When comparing the two risk groups using Kaplan–Meier survival curves, the groups showed significantly different survival (*P* < 0.001, log-rank; Fig. 4c): the high-risk group (*n* = 265) had 99 deaths over a 21-year horizon versus 58 in the low-risk group (*n* = 276).

**Extended Data Fig. 6:**
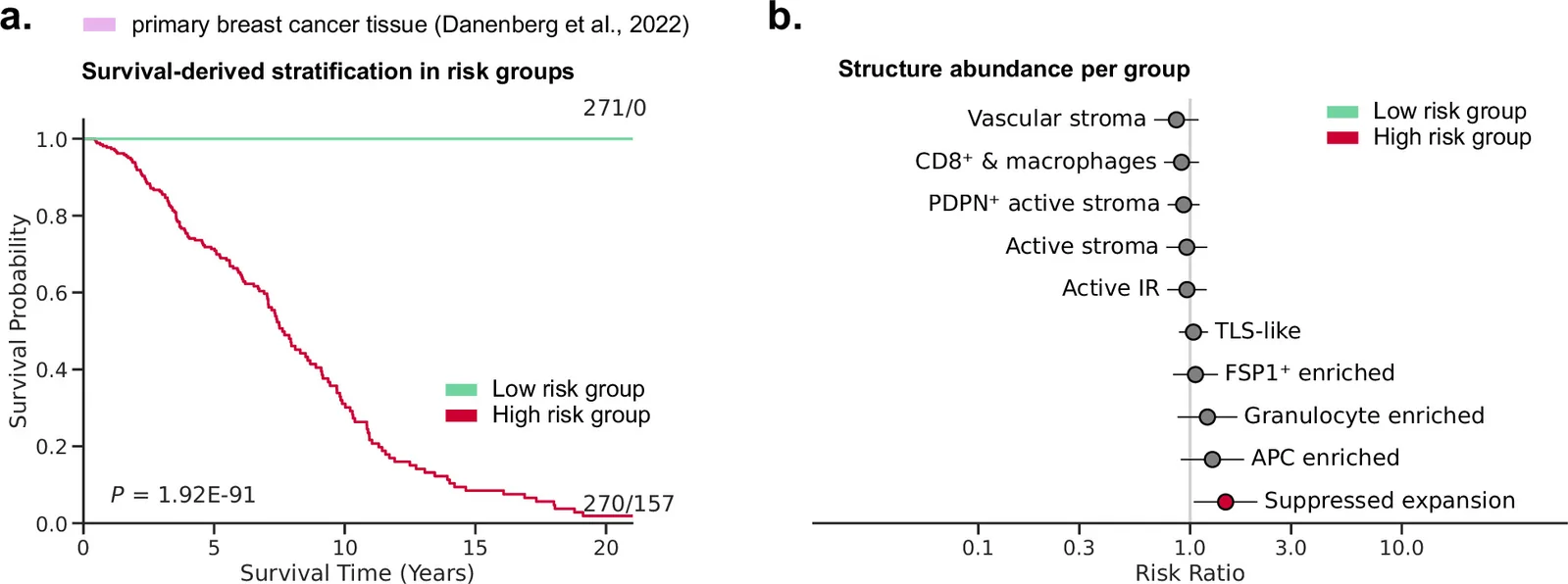
Risk stratification derived from survival data alone, without VirTues, on Danenberg et al.^6^. **a**, Kaplan-Meier survival curves for survival-derived control risk groups on Danenberg et al.^6^. Risk groups are formed by balanced grouping of (i) survivors and late-censored patients, and (ii) deceased and early-censored patients. The *P* value is computed via a log-rank test. **b**, Risk ratio of each TME structure’s occurrence in the high risk control group. Error bars indicate 95% Wald confidence intervals estimated from n=541 patients.

To test whether this VirTues-based stratification reflected known multi-cellular structures of the TME, we analysed it against the ten structures identified by Danenberg et al.^6^, four of which (vascular stroma, granulocyte-enriched, suppressed expansion, antigen-presenting cell (APC)-enriched) were identified previously as relevant to clinical outcome based on their hazard ratios. The risk ratios for each structure’s occurrence in the high-risk group (Fig. 4d) showed strong concordance between our stratification and these known prognostic structures: vascular stroma, linked to reduced hazard, was underrepresented (risk ratio 0.399), whereas the adverse suppressed expansion and APC-enriched structures were enriched markedly (4.464 and 5.636). By contrast, a stratification derived from survival data alone (without VirTues), separating deceased and early-censored from surviving and late-censored patients, showed no such tendency and only a mildly elevated risk ratio (1.472) for suppressed expansion (Extended Data Fig. 6).

## Predicting diagnostic and prognostic properties

Many clinical decisions are made at the scale of a patient’s tissue biopsy, where signals from many cells and regions must be combined into a single prediction. We evaluated VirTues’ tissue-level representation with attention-based multiple instance learning (ABMIL)^58^ across diagnostic and prognostic tasks (Fig. 4e). VirTues’ tissue-level predictions (Fig. 4f) were particularly strong on key clinical diagnostic tasks, including cancer subtyping and tumour grading on lung cancer and oestrogen receptor status determination and grading on breast cancer. For lung cancer in the datasest of Cords et al.^42^, it reached macro-*F*_1_ scores of 0.856 for subtyping and 0.530 for grading, improvements of +8.9% and +21.8% (both *P* = 0.0079) over the best baseline (KRONOS^35^). For breast cancer tissue from Danenberg et al. ^6^, it consistently surpassed all baselines: oestrogen receptor status (0.806, +14.2% over KRONOS^35^, *P* = 0.0119), ERBB2 (0.648, +2.0%, *P* = 0.1387), grading (0.490, +15.9%, *P* = 0.0158) and PAM50 subtyping (0.385, +8.2%, *P* = 0.1507), despite class imbalance affecting several of these tasks (Supplementary Fig. 21).

On the NeoTRIP TNBC cohort^2,59^, VirTues showed strong performance in predicting pathological complete response after 24 weeks of treatment. The detailed annotations allowed distinguishing patients who received both chemotherapy (carboplatin, nab-paclitaxel) and anti-PD-L1 immunotherapy (atezolizumab), as well as comparing performance across samples collected before, during and after treatment (Supplementary Fig. 21). On pre-treatment samples, VirTues reached a macro-averaged *F*_1_ of 0.676, outperforming KRONOS^35^ (+20.2%, *P* = 0.0157) and ResNet^26^ (+8.6%) and matching CA-MAE^31^ (0.685). On on-treatment samples it reached 0.714, improving on KRONOS^35^, CA-MAE^31^ and ResNet^26^ by +30.2%, +31.4% and +19.5% (all *P* = 0.0079). On post-treatment samples it reached 0.678, again exceeding KRONOS^35^ (+18.63%, *P* = 0.0079) and ResNet^26^, with a marginal gain over CA-MAE^31^. These findings show VirTues captures discriminatory tissue features predictive of treatment outcome and underscore its potential utility in clinical practice.

## Attention pinpoints markers and regions

**Extended Data Fig. 7:**
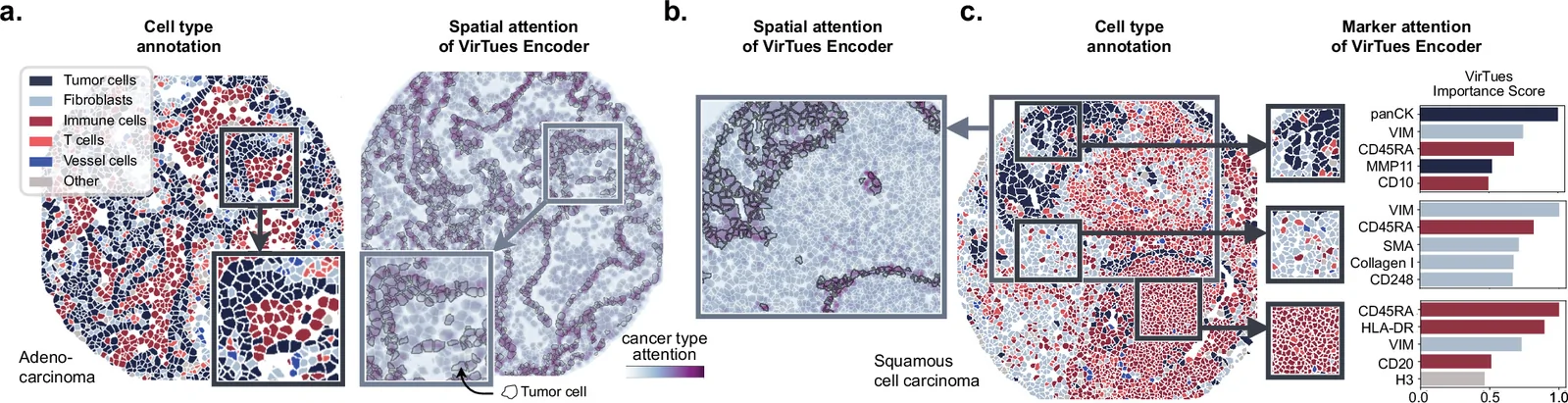
Visualization of spatial and marker attention in Cords et al.^42^. **a**, **b**, Spatial attention maps derived from an extended version of VirTues incorporating an additional class token that attends to all patch summary tokens. The model was fine-tuned for cancer subtype prediction and the shown maps represent the class token’s attention scores. **c**, Marker attention scores computed across three tissue niches with distinct cell type compositions. The five markers most attended to by other markers (after averaging attention scores across these regions) are shown. Bars are color-coded according to the canonical cell type associated with each marker.

Beyond predictive accuracy, both clinical trust and biological discovery require interpretable predictions, that is, knowing which markers and which regions drive each output. VirTues exposes two complementary attention readouts at inference and thus allows for such an analysis: spatial maps over the tissue and per-marker attention vectors (Extended Data Fig. 7). Fine-tuned for non-small cell lung cancer subtyping, its spatial maps formed coherent, anatomically plausible foci in adenocarcinoma and squamous cell carcinoma (Extended Data Fig. 7a,b), concentrating, as pathologists do, on tumour-rich epithelial compartments and tumour-immune interfaces while down-weighting stroma. Summarizing the marker attention vectors into ‘importance scores’ (Methods) recapitulated the dominant biology of each niche (Extended Data Fig. 7c). In immune-infiltrated squamous-carcinoma regions, the model weighted panCK and MMP11, consistent with epithelial carcinoma identity and aggressiveness^60,61^, and CD45RA and CD10, which mark T cell and B cell subsets^62,63^. In fibroblast-enriched stroma, vimentin, smooth muscle actin, collagen I and CD248 received the highest weights, aligning with fibroblast biology and extracellular-matrix remodelling^64,65^. In immune-dense regions, attention concentrated on CD45RA, HLA-DR and CD20—markers of immune identity and B cell compartments. Thus, spatial and marker attention give mutually reinforcing, region-specific explanations that align with the compartments and molecular readouts human experts rely on.

## Retrieval of clinically similar patients

To probe clinical utility, we used VirTues to retrieve clinically similar patients from a reference cohort for case-based decision support (Fig. 4g). VirTues’ niche summary tokens, trained to capture tissue composition, architecture and neighbourhood interactions, serve as a compact fingerprint of each tissue. Encoding a reference cohort of archival cases into these tokens forms a searchable ‘Virtual Tissues Database’, which we query by computing pairwise Wasserstein distances^66,67^ between a new patient’s tokens and the stored tokens, returning the closest matches together with their clinical records.

**Extended Data Fig. 8:**
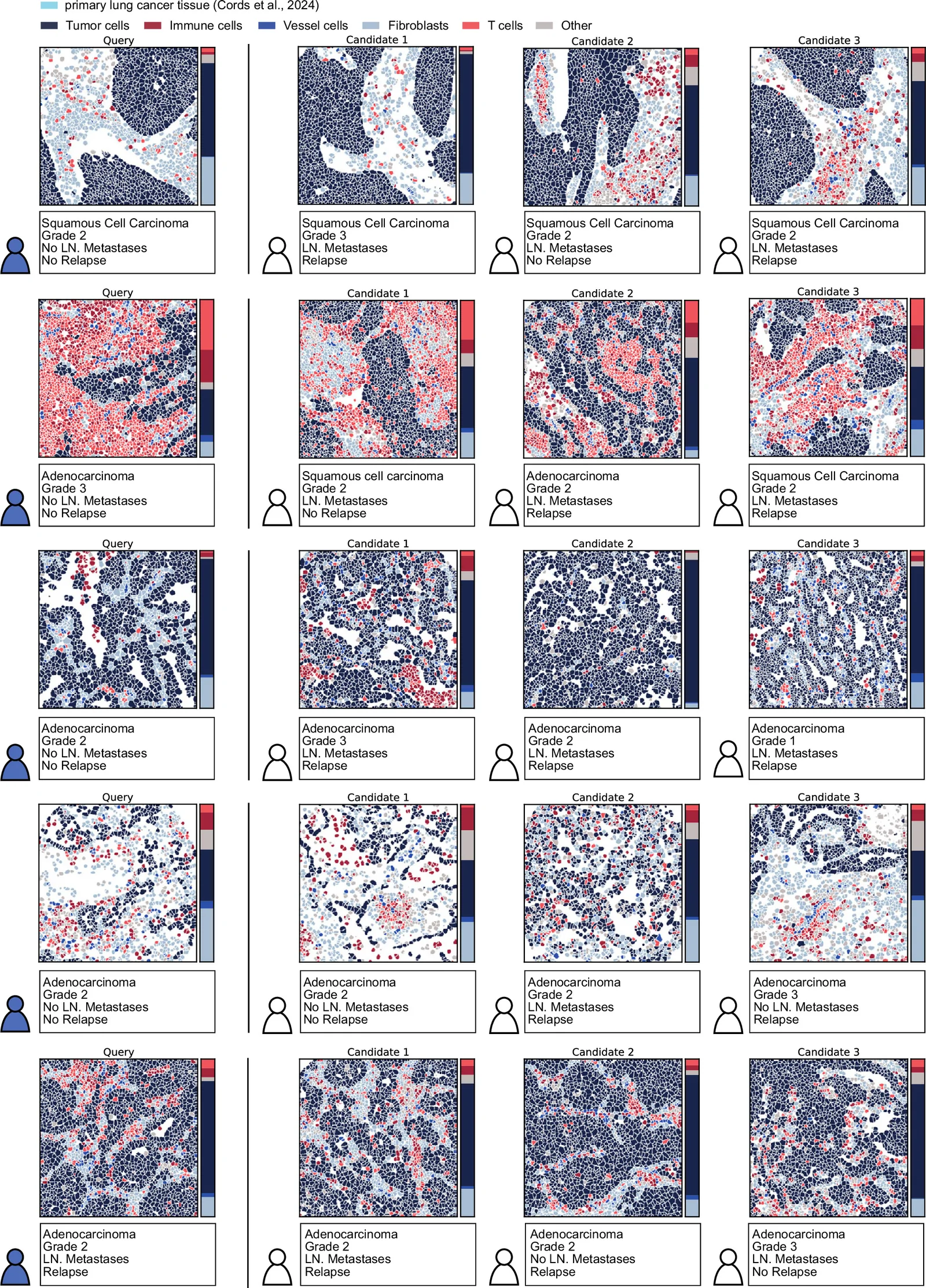
Examples of retrieval results for tissues in Cords et al.^42^ using the Wasserstein distance of VirTues’ niche representation tokens as the similarity measure. Tissues are visualized using their color-coded cell type masks. Colorbars show the proportional cell type compositions.

To quantify VirTues’ ability to retrieve appropriate samples, we designed three quantitative tasks and benchmarked VirTues against baselines (ResNet^26^, KRONOS^35^, CA-MAE^31^) and random retrieval on the lung cancer dataset of Cords et al.^42^. First, we compared whether retrieved samples matched the reference samples in their clinical and diagnostic variables (Fig. 4h): across all variables, VirTues achieved significantly higher matching rates than random retrieval (McNemar’s test, all *P* < 0.001), with particularly strong performance in matching cancer subtype, where it exceeded all baselines in mean precision. Second, comparing cell type distributions between retrieved and reference tissues by the L1 distance between normalized histograms, VirTues achieved the lowest distributional distance (Fig. 4i). Third, using an optimal transport metric that computes the sliced Wasserstein distance between raw pixel values of multiplex images^68^, VirTues retrieved archival samples of highly similar molecular composition, ranking second overall behind KRONOS^35^ (Fig. 4j). Three case studies (Fig. 4k; further examples in Extended Data Fig. 8) illustrate retrieval across cancer presentations:

1. A grade 2 adenocarcinoma matched to a case with similar grade, lymph node metastasis and relapse outcome (Fig. 4k, left);
2. A grade 2 squamous cell carcinoma matched on tissue architecture but differing in lymph node metastasis status and clinical trajectory (Fig. 4k, middle) and
3. A grade 2 adenocarcinoma matched on grade and trajectory but with distinct lymph node metastasis status (Fig. 4k, right).

Overall, retrievals from VirTues’ niche tokens capture tissue similarity at several scales, from molecular and cell type composition to clinical variables. VirTues retrieval prioritized cases that were similar both molecularly and architecturally to the query and enriched for the same cancer subtype, providing evidence that the learned similarity is clinically meaningful and establishing a quantitative, case-based framework for comparing tissue phenotypes across patients.

## Foundation model-derived biomarkers

To be predictive of therapy success, biomarkers should capture the complexity of the TME that reflects a patient’s response. Although classical biomarkers usually rely on single markers or cell type frequencies, virtual tissue representations encode intrinsically the microenvironment’s spatial and multi-cellular organization and can be mined for response biomarkers (Fig. 5a).

**Extended Data Fig. 9:**
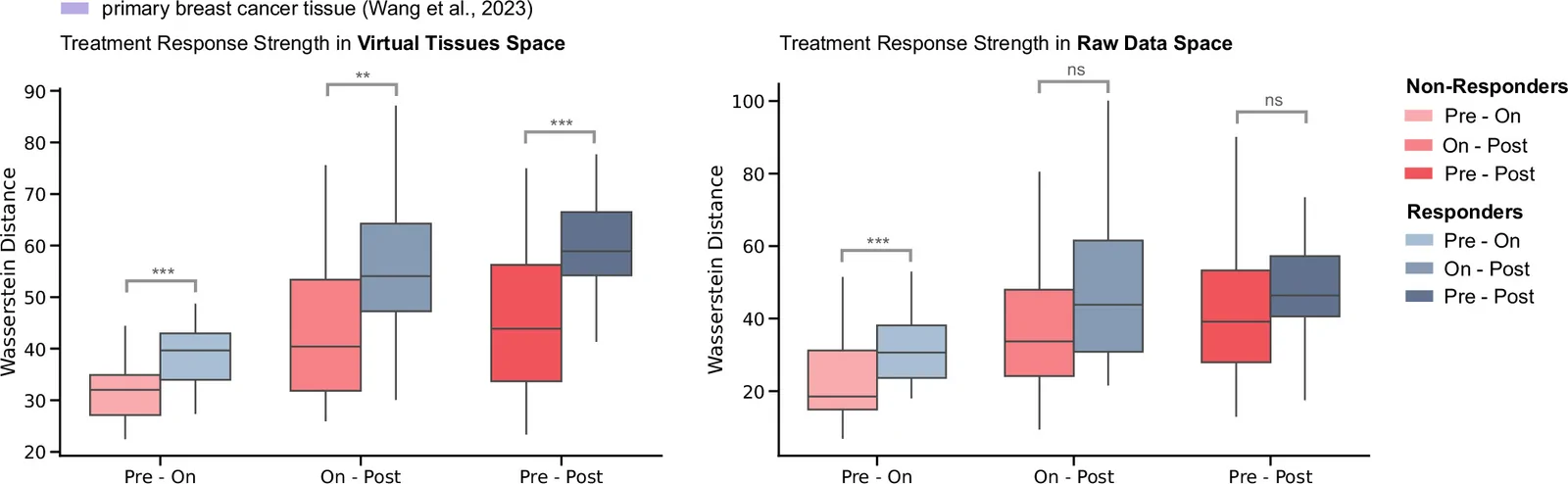
Comparison of response strengths of patients (responders and non-responders) to immunotherapy on Wang et al.^2^. Patient-level treatment response strength is quantified via the Wasserstein distance between cell embeddings, stratified by compared treatment stages, pathological complete response status, and embedding space. Boxes indicate quartiles; whiskers extend to 1.5 times the inter-quartile range beyond the box edges. Statistical significance (two-sided Mann-Whitney U test; n=68 patients for Pre-On, n=56 for On-Post and Pre-Post) is indicated above brackets: P<0.05 (*), P<0.01 (**), P<0.001 (***); ns indicates P≥0.05.

As a first sanity check, we tested whether VirTues captures changes that distinguish responders. Across pre-, on- and post-treatment samples in the NeoTRIP TNBC cohort^2,59^, VirTues’ embedding captured pronounced shifts that separated outcomes: responders remodelled far more than non-responders (UMAP with kernel-density overlays, Fig. 5b, left; further analysed through a two-sided Mann–Whitney *U* test across Wasserstein distances between consecutive biopsies comparing responders and non-responders in Extended Data Fig. 9). Raw mean marker intensities did not resolve this difference (Fig. 5b, right), thus these tissue dynamics are captured only by the foundation model representation.

We then derived VirTues-based biomarkers from pre-treatment biopsies through an unsupervised-then-supervised pipeline (Fig. 5c): we (1) embedded all pre-treatment cells with VirTues; (2) performed multi-resolution Leiden clustering to obtain phenotypic partitions; (3) aggregated cells per patient to compute cluster frequencies; and (4) scored each cluster’s predictive value for response using cross-validated, patient-level models, ranking by out-of-fold AUROC and response risk (Methods). From the top hits, we retained two response signatures (RS1, RS2) and two non-response signatures (NRS1, NRS2) that occupied distinct UMAP regions and comprised mixtures of cell types rather than single canonical labels (Fig. 5d). Individually they reached cross-validated AUROCs of 0.783, 0.707, 0.599 and 0.577; a multivariate model combining all four, the resulting VirTues-derived biomarker, reached an AUROC of 0.823, surpassing the spatial predictor of Wang et al.^2^ by +5.14% (*P* = 2.43 × 10^–4^) and classical clinical immune-ratio baselines^44,45^ by 26–32% (all *P* < 0.001; Fig. 5e).

Cell type composition distinguished the signatures (Fig. 5f and Supplementary Fig. 22): RS1 was enriched strongly in apoptotic cells (+415%), DNA-damaged cells (+497%) and PD-L1^+^GZMB^+^ cells (+607%), indicating a stressed, immune-targeted state, whereas RS2 was enriched for CD4^+^ T cells (+410%) and checkpoint-positive cells (PD-L1^+^GZMB^+^, +275%; PD-L1^+^ indoleamine 2,3-dioxygenase (IDO)^+^, +218%). Both non-response signatures were tumour-dominated (+328% and +358%) and depleted of immune and stromal cells. Their neighbourhoods reinforced this (Fig. 5g and Supplementary Fig. 23): in RS1, apoptotic cells were surrounded by more CD4^+^ T cells (+108%) and PD-L1^+^GZMB^+^ cells (+248%) and B/plasma-cell neighbourhoods were enriched strongly for PD-L1^+^GZMB^+^ (+1,226%) and epithelial–mesenchymal transition (EMT)-like tumour cells (+1,523%), consistent with active immune pressure, whereas RS2 CD4^+^ T cells sat among more tumour (+414%), major histocompatibility complex-I/II^+^ (+665%) and PD-L1^+^GZMB^+^ cells but fewer CD8^+^ T cells (−67%), consistent with a checkpoint-regulated T helper response. Thus, the two response signatures capture complementary anti-tumour mechanisms, a cytotoxic, stress-driven program (RS1) and a checkpoint-regulated T helper program (RS2), both indicating productive immune activation amplifiable by checkpoint blockade.

**Extended Data Fig. 10:**
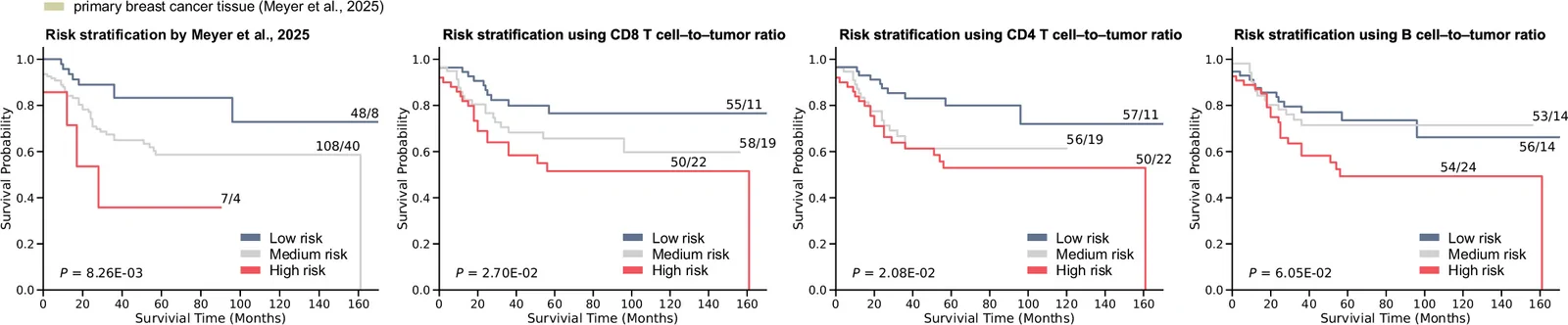
Kaplan–Meier curves of disease-free survival on Meyer et al.^3^ using different baseline risk stratification systems. From left to right: (1) original risk groups from Meyer et al.^3^, (2) risk scores based on tertile-transformed CD8 T cell–to–tumor ratios, (3) CD4 T cell–to–tumor ratios, and (4) B cell–to–tumor ratios. Each plot reports the *P* value from a log-rank test comparing the low- and high-risk groups.

To test generalization, we transferred the signatures to an independent TNBC cohort^3^ not used in pretraining or discovery, computing per-patient VirTues embeddings and applying random forest classifiers trained on the discovery cohort (Fig. 5h). As expected, response signatures localized to immune-inflamed regions and non-response signatures to immune-excluded areas (Fig. 5i). Lacking response labels, we evaluated disease-free survival: patient-level signature frequencies were combined into a single risk score, which stratified patients into three clearly separated groups (Kaplan–Meier *P* = 0.0037, log-rank; 3 events in low risk, *n* = 33, versus 21 in high risk, *n* = 45; Fig. 5j), reaching a concordance index of 0.628, exceeding the system of Meyer et al.^3^ (0.606) and ratio-based baselines, namely tumour-to-CD8 T cell (0.602), tumour-to-CD4 T cell (0.608) and tumour-to-B cell ratios (0.569) (Fig. 5k and Extended Data Fig. 10). The fact that signatures discovered de novo for treatment response also stratified disease-free survival in an independent cohort indicates that VirTues’ representation captures generalizable, prognostically meaningful disease biology rather than a cohort-specific artefact.

## Discussion

Multiplexed tissue imaging resolves the molecular state and spatial organization of individual tumour and immune cells, bringing the mechanisms of disease and new biomarkers that guide therapy within reach. Realizing this demands learning across the marker panels, platforms and cohorts fragmenting the field while serving diverse prediction and discovery tasks from a single representation. VirTues—a marker-aware foundation model trained on diverse IMC data (3,102 patients, 146 markers) and extended across four imaging platforms (more than 5,100 patients, 239 markers)—unifies these heterogeneous measurements into a single atlas of ‘virtual tissues’. From this atlas, it serves diverse prediction and discovery tasks through one representation, without task-specific fine-tuning. Three choices underpin this generality: PLM embeddings that encode marker identity and let the model relate panels it was never trained on, factorized spatial and marker attention that scales to highly multiplexed data, and hierarchical summary tokens that expose structure from cells to whole tissues.

The central advance of VirTues is ‘end-to-end’ coverage of the spatial proteomics workflow from a single backbone. On the earliest steps it matches or surpasses specialized tools: linear probes on its cell tokens outperform recent encoders for cell typing, including rare types and zero-shot settings, and segmentation heads transfer across cohorts with only partially overlapping panels, beating dedicated segmentation and intensity-based methods. The same representation then supports steps that classical pipelines handle separately or cannot perform at all: marker reconstruction and virtual panel augmentation^69^, niche annotation, tissue-level diagnosis and prognosis, retrieval of clinically similar cases for tumour boards^70^ and, most distinctively, automated biomarker discovery. Crucially, because the model encodes every marker through its protein sequence, the labels it produces, including segmentation masks, cell types and niche annotations, stay consistent across heterogeneous cohorts, panels and acquisition platforms, providing the substrate for building spatial proteomics atlases. Performance improved with corpus diversity—a hallmark of foundation models that argues for continued multi-dataset pretraining.

These capabilities culminate in the discovery of new clinical biomarkers. In TNBC, VirTues derived spatial signatures de novo from pre-treatment biopsies: a hypothesis-free pipeline identified response and non-response programs that predicted anti-PD-L1 immunotherapy response with an AUROC of 0.823, surpassing published spatial predictors and immune-ratio baselines. The same signatures transferred to an independent cohort to stratify disease-free survival and outperformed established clinical schemes. This represent a new generation of spatial biomarkers: rather than single markers or cell-type frequencies engineered from previous hypotheses^1,2,6^, they are discovered automatically yet remain retrospectively interpretable, capturing multi-cellular programs defined jointly by cell phenotype and spatial arrangement. Across cross-technology benchmarks spanning IMC, CODEX and Orion, VirTues alone generalized robustly and IMC-only training remained competitive with multi-technology baselines under large panel and technology shifts.

Several limitations remain. Zero-shot reconstruction degrades for markers with weak sequence relatedness to the training set, thus virtual augmentation should be calibrated and anchored to measured channels. Rare cell states and unusual tissue architectures also remain hard to capture, probably reflecting their scarcity in current corpora rather than a limit of the architecture. We expect targeted curation and continued data growth to close this gap. Our survival analyses, although supportive, are early proofs of concept, and covariate-adjusted modelling across independent cohorts together with prospective validation are needed before clinical use. For further discussion on limitations, see Supplementary Discussion.

Several directions follow naturally from this foundation: a first extension couples the deep molecular readout of spatial proteomics with scalable, routinely acquired H&E morphology^21^, bringing virtual tissues into routine pathology and uniting molecular depth with morphological breadth in one model. Further, combining virtual tissue representations with generative modelling and temporally resolved data extends spatial proteomic foundation models beyond static snapshots toward modelling the dynamics of disease progression and therapy^36^. More broadly, VirTues reframes multiplexed tissue imaging as a shared representation of tissue state across cohorts, helping to move spatial proteomics from isolated studies toward systematic, atlas-scale diagnostic and prognostic analysis and biomarker discovery.

## Methods

### VirTues architecture

Multiplexed imaging data pose modality-specific challenges to the development of scalable machine learning algorithms. The images represent high-dimensional samples, characterized by a large number of measured channels and high spatial resolutions. Further, the total number as well as the combination of channels varies between datasets as studies use different marker panels. These characteristics of the data modality hinder the simple off-the-shelf application of established vision architectures. Both convolutional neural networks and standard ViTs require a constant number of input channels, typically red, green and blue (RGB), with a fixed semantic meaning. Moreover, in contrast to RGB channels, which combine to produce colours, multiplex channels convey distinct biological meanings and exhibit complex inter-relationships. To address the unique challenges posed by multiplex imaging data, we propose VirTues—an encoder–decoder model based on the ViT architecture. VirTues is designed for the efficient processing of highly multiplexed image data accommodating varying numbers and combinations of measured markers. Furthermore, VirTues incorporates the attribution of distinct biological meaning to each measured marker. VirTues operates on tokenized image crops of size *d*_c_ × *d*_c_ = 128 × 128. Restricting VirTues’ input to such crops increases the number and diversity of pretraining samples while decreasing the dimensionality per sample.

#### Tokenization

To preserve the biologically distinct meaning of each channel and allow for a flexible number of channels per image, we used a multi-channel tokenization procedure^31,33^. Each channel is divided spatially into patches of size ${d}_{{\rm{p}}}\times {d}_{{\rm{p}}}=8\times 8$, as this captures approximately one cell per patch. Flattening each patch results in a three-dimensional grid of image tokens ${\bf{x}}\in {{\mathbb{R}}}^{M\times H\times W\times {d}_{{\rm{p}}}^{2}}$, where M is the number of measured channels and $H=W={d}_{{\rm{c}}}/{d}_{{\rm{p}}}$ the grid height and width. For all M markers measured by the channels of ${\bf{x}}$, we retrieve from a pre-computed lookup table the corresponding protein embeddings $\pi \in {{\mathbb{R}}}^{M\times {d}_{{\rm{PLM}}}}$ given by the PLM (ESM-2 (ref. ^18^) with ${d}_{{\rm{PLM}}}=640$). We refer to these embeddings as marker tokens. For each channel m and each grid position (i,j), we project the image token ${{\bf{x}}}_{{mij}}$ and the corresponding marker token ${\pi }_{m}$ to the same dimension ${d}_{{\rm{model}}}$ using learnable linear projections, to get ${{\bf{x}}}_{{mij}}^{{\prime} }\in {{\mathbb{R}}}^{{d}_{{\rm{model}}}}$ and ${\pi }_{m}^{{\prime} }\in {{\mathbb{R}}}^{{d}_{{\rm{model}}}}$, respectively. The image and marker tokens are fused through summation, resulting in the image tokens $\widetilde{{\bf{x}}}\in {{\mathbb{R}}}^{M\times H\times W\times {d}_{{\rm{model}}}}$, where ${\widetilde{{\bf{x}}}}_{{mij}}={{\bf{x}}}_{{mij}}^{{\prime} }+{\pi }_{m}^{{\prime} }$. The fusion of the marker token with the image tokens serves two main purposes: (1) enabling VirTues to differentiate the channel origins of input tokens, and (2) introducing a biologically informed prior, reflecting sequence-level protein relationships, which cannot be added through other marker tokenization schemes (such as one-hot or learnable marker embeddings). We note that this is the first of many building blocks enabling VirTues to generalize across unseen markers. Further, to allow VirTues to capture an aggregated representation for each patch, we introduce an additional layer of learnable patch summary tokens ${\bf{c}}\in {{\mathbb{R}}}^{H\times W\times {d}_{{\rm{model}}}}$, one for each spatial position. Each patch summary token ${{\bf{c}}}_{{ij}}\in {{\mathbb{R}}}^{{d}_{{\rm{model}}}}$ is initialized using the same weights.

#### Masking

During training, a portion of the image tokens $\{{\widetilde{{\bf{x}}}}_{{mij}}\}$ is masked by replacing them with a special masking token ${\rm{\square }}\in {{\mathbb{R}}}^{{d}_{{\rm{model}}}}$ initialized with learnable weights. Masking is applied channel-wise by sampling a masking ratio ${r}_{{\rm{masking}}}$ between 60% and 100% and uniformly selecting the corresponding $\lceil {r}_{\mathrm{masking}}HW\rceil$ tokens to mask within the channel. We denote the resulting three-dimensional binary mask by ${\bf{M}}\in \{0,1{\}}^{M\times H\times W}$, where the value 1 marks masking. Masked tokens remain linked to their specific markers, which is indicated by adding the marker tokens to the masked tokens.

#### VirTues encoder

The set of all non-masked image tokens $\{{\widetilde{{\bf{x}}}}_{{mij}}| {{\bf{M}}}_{{mij}}=0\}$ and the set of patch summary tokens $\{{{\bf{c}}}_{{ij}}\}$ is passed as an input to the VirTues encoder. This encoder is constructed by modifying the vision transformer’s architecture^14^, to adapt it to work with varying input markers efficiently, and capture marker correlations and spatial patterns separately. In contrast to standard ViTs, which use full multi-head self-attention where all tokens attend pairwise to each other, we use two specialized sparse multi-head self-attention mechanisms—marker attention and spatial attention, akin to space and time attention used in video transformers^40^. In marker attention, only tokens that are placed at the same spatial grid position attend to each other, thereby capturing inter-marker dependencies and correlations. We denote the set of input tokens to the ${\ell }$ th transformer block as $\{{t}_{mij}^{{\ell }}\}$, where the token ${t}_{mij}^{{\ell }}$ is associated to the m-th channel and position (i,j). In this notation, we treat the layer of patch summary tokens simply as a further channel. Then, a marker attention transformer block computes

$${\rm{\forall }}{i}^{\ast },{j}^{\ast }:\{{t}_{mij}^{{\ell }+1}\,|i={i}^{\ast },j={j}^{\ast }\}={\rm{M}}{\rm{H}}{\rm{S}}{\rm{A}}(\{{t}_{mij}^{{\ell }}\,|i={i}^{\ast },j={j}^{\ast }\}).$$

where MHSA denotes a transformer block with standard multi-head self-attention. By contrast, in spatial attention, only tokens belonging to the same channel attend to each other hence capturing spatial patterns across tissue. Following the notation for marker attention, a spatial attention transformer block computes

$${\rm{\forall }}{m}^{\ast }:\{{t}_{mij}^{{\ell }+1}\,|m={m}^{\ast }\}={\rm{M}}{\rm{H}}{\rm{S}}{\rm{A}}(\{{t}_{mij}^{{\ell }}\,|m={m}^{\ast }\}).$$

The VirTues Encoder architecture consists of a sequence of 16 transformer blocks, which alternate between blocks that use marker and spatial attention. Each of the transformer blocks uses eight attention heads. Spatial positions are encoded using two-dimensional rotatory position embeddings^71^. Further, we use pre-layer normalization^72^.

The VirTues Encoder outputs a set of encoded image tokens $\{{\mathop{{\bf{x}}}\limits^{ \sim }}_{mij}^{{\rm{e}}{\rm{n}}{\rm{c}}}\,|\,{{\bf{M}}}_{mij}=0\}$ and a set of encoded patch summary tokens $\{{{\bf{c}}}_{{ij}}^{{\rm{enc}}}\}$.

#### VirTues decoder

The VirTues decoder is used during training and inference to reconstruct the original image or generate new channels. It comprises a ViT^14^ followed by a single linear projection. To reconstruct the original image, the encoded tokens $\{{\widetilde{{\bf{x}}}}_{{mij}}^{{\rm{enc}}}| {{\bf{M}}}_{{mij}}=0\}$, the encoded patch summary tokens $\{{{\bf{c}}}_{{ij}}^{{\rm{enc}}}\}$ and the masked tokens $\{{\widetilde{{\bf{x}}}}_{{mij}}| {{\bf{M}}}_{{mij}}=1\}$ (after replacement with the masking token) are regrouped as follows: for each channel ${m}^{* }$, we group the encoded and the masked tokens of that channel with a copy of the encoded patch summary tokens

$$\begin{array}{c}\{{\mathop{{\bf{x}}}\limits^{ \sim }}_{{m}^{\ast }ij}^{{\rm{e}}{\rm{n}}{\rm{c}}}\,|{{\bf{M}}}_{{m}^{\ast }ij}=0\}\cup \{{\mathop{{\bf{x}}}\limits^{ \sim }}_{{m}^{\ast }ij}\,|{{\bf{M}}}_{{m}^{\ast }ij}=1\}\cup \{{{\bf{c}}}_{ij}^{{\rm{e}}{\rm{n}}{\rm{c}}}\}.\end{array}$$

These groups of tokens are passed individually to the decoder one by one. Hence, in the decoder, tokens of different channels do not interact with each other. This design forces the decoder to reconstruct each channel primarily from the patch summary tokens rather than from other channels, incentivizing the encoder to store a meaningful representation in these tokens. This regrouping further limits the individual token set sizes to $2{HW}$, thus allowing us to use full MHSA instead of marker and spatial attention. Processing all tokens by the decoder’s transformer followed by the linear projection yields the final grid of reconstructed image tokens ${{\bf{x}}}^{{\rm{rec}}}\in {{\mathbb{R}}}^{M\times H\times W\times {d}_{{\rm{p}}}^{2}}$.

During inference, VirTues can generate new channels by appending randomly initialized channels to the input $\mathrm{x}$, masking all tokens in these channels, indicating their targets using marker tokens, and reconstructing them using the VirTues Encoder and Decoder.

Following He et al.^15^, we setup the encoder–decoder framework in an asymmetric fashion, where the size of the encoder is deeper than the decoder, allowing the main workload of the model to rely on the encoder rather than the decoder. We construct a shallow decoder consisting of 4 transformer blocks with full attention (in contrast to 16 transformer blocks in the encoder with alternating marker and spatial attention). Similar to the encoder, we use two-dimensional rotatory position embeddings^71^ to encode spatial positions and pre-layer normalization^72^ in the decoder.

#### Aggregation into cell-, niche- and tissue-level representations

During inference, VirTues Encoder represents each image crop as a grid of patch summary tokens ${{\bf{c}}}^{{\rm{enc}}}\in {{\mathbb{R}}}^{H\times W\times {d}_{{\rm{model}}}}$. These are aggregated into cell-, niche- and tissue-level representations as follows: for cell-level representations, the full multiplexed image is divided into a grid of overlapping crops of size 128 × 128 using a stride of *s* = 42. Next, each crop is embedded independently. Finally, for each cell, the cell summary token is computed as a weighted average of all patch summary tokens, where the weight assigned to each patch equals the number of pixels intersecting with the cell in that patch. For niche- and tissue-level representations, the image is divided into a grid of non-overlapping crops of size 128 × 128, crops covered by less than 30% of tissue are excluded, and the remaining crops are embedded individually. The niche or tissue-level representations are then obtained by aggregating all encoded patch summary tokens from the crop or the full image, respectively. For unsupervised tasks, such as the retrieval experiments (Fig. 4g–k), a simple average $z=\frac{1}{{HW}}\sum _{i,j}{{\bf{c}}}_{i,j}^{{\rm{enc}}}$ is used for aggregation, generating task-agnostic embeddings. In supervised settings, a dynamically weighted average is used, achieved by training an ABMIL classifier^58^ on the given task (while the parameters of VirTues’ are kept frozen), generating task-specific embeddings. This attention weighted average computes as

$$\begin{array}{c}{\rm{z}}=\sum _{i,j}{a}_{ij}{{\bf{c}}}_{ij}^{{\rm{e}}{\rm{n}}{\rm{c}}}\\ {a}_{ij}=\frac{\exp \,{w}^{T}(\tanh (V{{\bf{c}}}_{ij}^{{\rm{e}}{\rm{n}}{\rm{c}}})\odot \sigma (U{{\bf{c}}}_{ij}^{{\rm{e}}{\rm{n}}{\rm{c}}}))}{\sum _{i{\prime} j{\prime} }\exp \,{w}^{T}(\tanh (V{{\bf{c}}}_{{i}^{{\prime} }{j}^{{\prime} }}^{{\rm{e}}{\rm{n}}{\rm{c}}})\odot \sigma (U{{\bf{c}}}_{{i}^{{\prime} }{j}^{{\prime} }}^{{\rm{e}}{\rm{n}}{\rm{c}}}))}\end{array}$$

where $U,V\in {{\mathbb{R}}}^{{d}_{{\rm{hidden}}}\times {d}_{{\rm{model}}}}$ and $w\in {{\mathbb{R}}}^{{d}_{{\rm{hidden}}}}$ are learnable weights, σ is the sigmoid activation function and ⊙ indicates element-wise multiplication. In a multi-head setting, this computation is repeated for each head with a different weight vector ${w}^{h}\in {{\mathbb{R}}}^{{d}_{{\rm{hidden}}}}$ and the resulting representations are concatenated. Per default, we use eight heads and ${d}_{{\rm{hidden}}}=256$.

#### Segmentation module

VirTues panoptic segmentation module consists of two parallel branches for cell instance segmentation and cell type prediction. Both branches build upon the U-Net-style decoder introduced in CellViT^73^ and successively process the internal patch summary tokens of VirTues, ${{\bf{c}}}^{{\ell }}\in {{\mathbb{R}}}^{H\times W\times {d}_{{\rm{model}}}}$, extracted after transformer blocks ${\ell }\in \{16,12,8,4,1\}$. Given (K−1) cell type classes, the cell typing branch predicts a dense mask ${{\bf{s}}}_{{\rm{celltype}}}\in {{\mathbb{R}}}^{{d}_{{\rm{c}}}\times {d}_{{\rm{c}}}\times K}$ containing pixel-wise class logits, including an additional background class. Pixel-wise class probabilities and label assignments are obtained through softmax and argmax operations, respectively. The background class is used only during training; at inference time, its logits are set to −∞, as background handling is delegated to the instance segmentation branch. The instance segmentation branch predicts a five-channel feature map ${{\bf{s}}}_{{\rm{instance}}}\in {{\mathbb{R}}}^{{d}_{{\rm{c}}}\times {d}_{{\rm{c}}}\times 5}$, which is converted subsequently into cell instance masks following the post-processing procedure of InstanSeg^50^.

#### Implementation details

To implement marker and spatial attention efficiently, we reduce these mechanisms to full attention by merging either the spatial or channel axis of the batched token tensor with the batch axis, allowing subsets of tokens that attend to each other to be treated as independent sequences. During inference without masking, this reduction leverages built-in, hardware-optimized implementations of standard self-attention. However, during training, channel-wise independent masking with varying ratios and channel dropout lead to token sequences in the marker and spatial attention blocks having variable lengths. This variability poses a technical challenge because efficient built-in PyTorch attention mechanisms require uniform sequence lengths within a batch. To avoid the computational overhead of adding padding tokens, we used a dynamic re-packaging strategy in conjunction with the support of Flash Attention-2 (ref. ^74^) support for block-diagonal masked self-attention. Non-masked tokens within a batch are repacked into a single sequence, preserving coherent subsequences of tokens that belong to the same sample and channel or spatial position. A block-diagonal mask is generated dynamically to indicate the subsequences, specifying which tokens can attend to each other. The repacked sequence and associated mask are processed using Flash Attention-2’s masked self-attention implementation.

### VirTues pretraining

#### Loss function

VirTues is trained end-to-end to reconstruct image crops in a masked autoencoding framework^15,75^. Our reconstruction loss is the mean squared error between the reconstructed pixels’ intensity values and the original pixels’ intensity values, that is,

$$\begin{array}{r}{{\mathcal{L}}}_{{\rm{MAE}}}=||{{\bf{x}}}^{{\rm{rec}}}-{\bf{x}}|{|}_{2}^{2}.\end{array}$$

Note that this loss is computed over all pixels of both masked and non-masked tokens.

#### Data augmentation

Before training, we first sample randomly from each tissue image four *N* sub-images of dimension 256 × 256, where *N* is the number of such sub-images fitting within the tissue image. Sub-images that are covered by less than 30% tissue according to the tissue segmentation mask are filtered out. During training, we sub-sample uniformly at random crops of size 128 × 128 from the sub-images. This hierarchical two-step sub-sampling method approximates sampling crops uniformly at random from the whole image, while avoiding an input/output-bottleneck while training. We further apply random rotations and flips to each selected crop. Moreover, to ensure VirTues learns representations robust to varying combinations of markers and enhance its ability to generalize to unseen datasets and markers, we sample uniformly a marker dropout ratio ${r}_{{\rm{dropout}}}$ between 0 and 25% and exclude a corresponding number of random channels from the training sample.

#### Optimization

We trained VirTues for 150 epochs using AdamW^76^ with an effective batch size of 512. Each epoch involved iterating once over all pre-computed sub-images. Weight decay is applied to all weights except biases and layer normalization terms, following a cosine schedule starting at 0.04. The learning rate follows a cosine decay starting at 0.0002. Training uses automatic mixed precision with 16-bit floating point precision. Gradients are clipped to a maximum norm of 1.0.

### Segmentation training

#### Loss function

Using a pretrained and frozen instance of VirTues, the instance segmentation and cell typing branches of the segmentation module are trained jointly, each with its own objective function. For the instance segmentation branch, we directly adopt the InstanSeg^50^ loss.

For the cell typing branch, let $\hat{p}$ denote the predicted pixel-wise class probabilities and p the corresponding ground-truth probabilities. To optimize cell type prediction, we use a combination of the focal Tversky loss^73^ and cross-entropy loss. The focal Tversky loss is defined as

$$\begin{array}{r}{{\mathcal{L}}}_{{\rm{FT}}}=\mathop{\sum }\limits_{k=1}^{K}{w}_{k}{\left(1-\frac{{{\rm{TP}}}_{k}+\varepsilon }{{{\rm{TP}}}_{k}+\alpha {{\rm{FN}}}_{k}+\beta {{\rm{FP}}}_{k}+\varepsilon }\right)}^{\gamma },\end{array}$$

where ${{\rm{TP}}}_{k}=\mathop{\sum }\limits_{i=1}^{N}{p}_{{ik}}{\hat{p}}_{{ik}}$, ${{\rm{FN}}}_{k}=\mathop{\sum }\limits_{i=1}^{N}{p}_{{ik}}(1-{\hat{p}}_{{ik}})$, and ${{\rm{FP}}}_{k}=\mathop{\sum }\limits_{i=1}^{N}(1-{p}_{{ik}}){\hat{p}}_{{ik}}$. We set the hyperparameters to α=0.7, β=0.3, and $\gamma =\frac{4}{3}$. Class weights w are set to 1 except for special classes corresponding to background or ‘Unknown’ where we use 0.05. The cross-entropy loss is given by

$$\begin{array}{r}{{\mathcal{L}}}_{{\rm{CE}}}=-\frac{1}{N}\mathop{\sum }\limits_{i=1}^{N}\mathop{\sum }\limits_{k=1}^{K}{p}_{ik}\,\log ({\hat{p}}_{ik}).\end{array}$$

The final cell typing objective is computed as an equally weighted combination of the two losses

$$\begin{array}{r}{{\mathcal{L}}}_{{\rm{cell}}-{\rm{typing}}}=\frac{1}{2}{{\mathcal{L}}}_{{\rm{FT}}}+\frac{1}{2}{{\mathcal{L}}}_{{\rm{CE}}}.\end{array}$$

#### Optimization

We train the segmentation module for 100 epochs, on random crops similar to the pretraining, using AdamW, with an effective batch size of 64 and a learning rate of 0.001.

### Datasets for VirTues development

#### Dataset curation

We release two instances of VirTues, each pretrained on one of two data collections: an IMC-only corpus, which underlies most of the analyses in this work, and an extended multi-technology corpus that additionally spans CODEX, Orion and MIBI. Both models and both data collections are publicly available.

For the IMC-only corpus, we curated 14 publicly available datasets including datasets from lung cancer^8,42,53,77–79^, breast cancer^2,6,52,53,80^, colon^53^, kidney^53^, head^53^, neck^53^ and primary and metastatic melanoma^48,56^, as well as non-cancerous tissues such as tonsil and endometrium^78^, and both healthy and diabetic pancreas^81^. A 15th IMC dataset, consisting of primary breast cancer tissue samples^3^, was collected after pretraining and the main analysis to assess whether the identified predictive spatial biomarkers generalize to an independent cohort. Images smaller than 256 × 256 pixels were excluded. Furthermore, we filtered out images with insufficient tissue coverage, for example those resulting from tearing or damage to the tissue micro-array cores. After this processing step, the IMC data corpus encompasses a total of 8,887 distinct images, comprising 3,102 patients and 146 distinct markers.

The extended multi-technology corpus extends this collection to evaluate VirTues’ cross-technology generalization and assess its robustness to multi-technology pretraining (Extended Data Fig. 1). To the IMC-only corpus, it adds seven CODEX^37^ datasets^44,82–87^, one Orion dataset^4^ (Supplementary Table 2) and two MIBI datasets^88,89^ as well as seven additional IMC datasets^90–96^. The expanded harmonized spatial proteomics corpus across four imaging technologies spans 32 cohorts, more than 5,100 patients and 239 protein markers.

An overview of all datasets used, their tissue origin as well as their sample sizes in terms of patients, images, sampled crops and annotated cells, can be found in Supplementary Tables 1 and 2.

For downstream tasks, we used the segmentation masks, cell labels and clinical annotations provided by the original studies. During quality control of the dataset from Cords et al.^42^, we identified a substantial proportion of inaccurately labelled cells. To address this issue, we re-annotated the dataset using a random forest classifier trained on a subset of 96 images that had been verified manually to contain accurate labels. In the dataset from Danenberg et al.^6^, we corrected misalignment issues by re-aligning the segmentation masks and cell annotations using the spatial coordinates provided.

Furthermore, for each tissue sample, we generated an approximate binary tissue segmentation mask using Otsu thresholding of the pixel-wise max-projection followed by binary opening for noise removal and binary closing for automatic hole filling^97^.

For each dataset, we compiled a list of markers corresponding to the image channels; when several markers corresponded to the same protein (for example, H3 and pH3), we retained a single marker, selecting the one we considered most informative. For each marker, we identified the canonical amino acid sequence from UniProt and computed its ESM-2 (ref. ^18^) embedding. For mRNA markers, we used the sequences of the encoded proteins. To facilitate cell segmentation, when histone H3 was not measured in a dataset, we instead included a non-protein nuclear marker (for example, Ir191, Ir193 or Hoechst), mapping it to the amino acid sequence of histone H3.

Each pretraining dataset was divided randomly into an 80/20 train/test split, ensuring that all samples from a given patient were contained entirely within either the training or the testing set. This split was used for the pretraining of VirTues and the baselines as well as for all evaluations and downstream experiments involving trainable parametric models.

#### Dataset preprocessing

For each image, intensity values are clipped channel-wise at the 99th percentile, followed by a shifted logarithm transformation with a size factor of 1, as commonly applied to single-cell RNA sequencing count data^98^. Next, each image is standardized channel-wise^31,99^ using means and s.d. values computed over each dataset. The image-wise channel percentiles as well as the dataset-wise means and s.d. values used in this preprocessing were computed only over the tissue area as defined by the tissue segmentation masks. Finally, a Gaussian blur filter with a kernel size of 3 and unit variance is used to smooth each image.

### Models, baselines and scaling

#### VirTues model instances

We have released two versions of VirTues, pretrained on an IMC-only and a multi-technology dataset corpus. The IMC-only model, used in most experiments unless stated otherwise, was trained on all 14 IMC datasets. To support specific analyses, we also trained several variants of it: for zero-shot evaluation (applying the model to a held-out dataset), instances each excluding one of refs. ^2,6,8,42,48^ in turn; for the single- versus multi-dataset comparison, an instance trained on data from Danenberg et al.^6^ alone (Fig. 3c); and for the channel-count study (Fig. 1e), instances trained on data from Cords et al.^42^ with marker panels restricted to 10, 20 or 40 markers (Supplementary Table 3). The multi-technology model was instead trained on the extended corpus spanning all 32 cohorts across the four imaging technologies, and we used it to evaluate the robustness of multi-technology training and cross-technology generalization (Extended Data Fig. 1 and Supplementary Tables 1 and 2).

#### Comparisons and baselines

We compare VirTues primarily with three deep learning baselines: (1) ResNet^26^, (2) CA-MAE^31^ and (3) KRONOS^35^. These baselines were selected as they are, similar to VirTues, self-supervised representation learning models recently developed for, or applied to, microscopy images. We note, however, that CA-MAE^31^ was developed originally with the intended application of learning representations from Cell Painting and brightfield microscopy data, not highly multiplexed images.

First, we used a pretrained ResNet^26^ based on the approach described in ref. ^26^. Specifically, each channel is embedded individually using the ResNet50 (ref. ^100^) architecture, pretrained on ImageNet-1K^101^, where each channel is duplicated three times to match the input dimension. The resulting ResNet50 embeddings were then concatenated and projected to their nine principal components using sparse mini-batch PCA to generate the crop representation. We remark that ResNet is a convolutional neural network that generates spatially aggregated niche-level representations directly, and is thus unable to embed patches at the cell-level. Hence, we compare against ResNet^26^ only for niche-level and tissue-level tasks. Furthermore, as it uses a pretrained network and is channel-agnostic, we also compare against ResNet for zero-shot experiments.

Second, we used CA-MAE proposed by Kraus et al.^31^. This model adopts a multi-channel tokenization strategy and an encoder–decoder framework similar to that of VirTues, but with key differences: each channel is assigned a separate decoder, marker identities are not encoded in the tokenization and full attention is used. These design choices restrict the model’s capability to scale to a large number of channels, imposes efficiency issues and hinders the model’s ability to zero-shot to unseen markers or datasets. We pretrain CA-MAE^31^ with the same reconstruction objective and procedure described in Kraus et al.^31^, setting the patch size to eight to capture information at the cellular scale. We note that we train CA-MAE^31^ for each dataset separately to address scaling issues and mitigate the computational bottlenecks caused by the unequal representation of channels in datasets, which would otherwise lead to a disproportionate increase in model parameters without a corresponding increase in data. From Fig. 1e, we notice that the number of parameters in VirTues is already 25 times less than CA-MAE^31^ for 40 markers. CA-MAE^31^ can be used to generate both patch-level and niche-level representations. For patch-level representations, we average the embedded tokens along the channel dimension. For self-supervised niche-level representations, we take the average of all embedded tokens of the crop.

Third, we compare against KRONOS^35^—a foundation model for spatial proteomics. As the published model instance of KRONOS^35^ was not trained on IMC data, we re-train KRONOS^35^ on our collection of pretraining IMC datasets. We use the publicly released model of Shaban et al.^35^ for the multi-technology benchmark. Pretraining of KRONOS^35^ requires each training sample to contain a nucleus marker. As such we use histone H3 and exclude three pretraining datasets not measuring consistently this marker, namely, datasets in refs. ^8,77,78^. We follow the original training protocol of KRONOS^35^ with one modification: KRONOS^35^ is applied to fluorescence-based imaging with resolutions between 0.37 and 0.5 µm per pixel, using a crop size of 256 × 256 pixels. By contrast, IMC data have a resolution of 1 µm per pixel. To ensure equivalent physical field of views, we configure KRONOS^35^ with a crop size of 128 × 128, a local view size of 48 × 48 and a patch size of 8 × 8 pixels. These parameters further match those of VirTues, thereby enabling a direct comparison between representations obtained for the same visual input. For patch-level representations, we average the token-specific features of KRONOS^35^ per spatial position, akin to CA-MAE^31^. For the niche-level representation, we concatenate the marker-specific features and project the resulting embeddings to their leading 256 principal components, following the same procedure as used by Shaban et al.^35^ for patient stratification. In summary, for all IMC-based evaluation tasks, we use and report results from the retrained KRONOS^35^ instance; for non-IMC-based evaluation tasks, we use the model released by Shaban et al.^35^.

#### Scaling analysis

To analyse the impact of the number of measured markers on both computational costs and prediction performance, we select nested subsets of 10, 20 and 40 markers from the original full panel used by Cords et al.^42^, based on their presumed informativeness regarding general tissue morphology and cell type differentiation. This selection is guided by previous knowledge and domain expertise. However, we acknowledge that this process is inherently subjective, as a quantitative framework to rank markers objectively by ‘informativeness’ does not exist. For a full list of markers per experiment, see Supplementary Table 3. We train instances of VirTues and CA-MAE^31^ on these chosen subsets on the dataset of Cords et al.^42^, and report the inference computational cost, number of parameters and downstream performance upon scaling the number of channels (Fig. 1e). We measure the computational cost c=m×t, where m is the memory used during forward pass of a batch of 16 images, and t is the inference time for the batch. We allow ten warm-up runs to remove graphics processing unit (GPU) startup effects, and report the average of 100 iterations. We further report the downstream macro-averaged *F*_1_ scores achieved by linear probes for cell-type classification using both coarse and fine-grained classes. We further evaluated the effects of single-dataset versus multi-dataset pretraining by training a model exclusively on primary breast cancer tissues^6^, and assessing its cell type classification performance using class-specific *F*_1_ scores (Fig. 3c).

### Evaluation

#### Masked reconstructions

We evaluate VirTues’ understanding of molecular tissue structure and biological relationships between markers by assessing the reconstruction ability of VirTues for three different masking strategies: independent masking, marker masking and niche masking. For independent masking (Fig. 2a and Supplementary Figs. 3–5), we sample a masking ratio independently for each channel in the input image, uniformly between 60% and 100% and mask the corresponding number of patches. This strategy allows the model to leverage both spatial patterns and marker relationships to reconstruct the masked regions, aligning with the masking used during training. For marker masking (Fig. 2b and Supplementary Figs. 6–8), we select a single marker from the input image and mask all corresponding patches. This approach enables us to evaluate VirTues’ understanding of marker correlations in isolation of its spatial understanding. In reverse, niche masking (Fig. 2c and Supplementary Figs. 9–11) is designed to analyse VirTues’ understanding of spatial structures. For each image, we sample a single masking ratio uniformly between 60% and 100% and use this ratio to select the corresponding number of grid positions, where we mask all patches across all channels. We emphasize that, in all three masking strategies, channel dropout is not applied during the evaluation phase. To quantify the reconstruction ability, we compute Pearson correlation and mean square error between reconstruction and ground truth images on the test split, per dataset, marker and masking strategy (Fig. 2d and Supplementary Figs. 1 and 2). In contrast to the training loss, we compute these metrics only for the masked tokens’ pixels to ensure comparability across masking strategies, despite varying relative masking ratios. As a baseline for independent and niche masking, we calculate the performance obtained when masked pixels in each channel are inpainted using the average intensity of the visible pixels in that channel. Further, we report for each dataset and marker, the performance reached by predicting under marker masking for each channel the most highly correlated alternative marker.

To characterize when zero-shot reconstruction of a new marker is expected to succeed, we assess how reconstruction quality varies with the marker’s proximity to the markers observed during pretraining in ESM-2 embedding space. For each such new marker, present only in the held-out dataset and never observed during pretraining, we compute the average L2 distance between its ESM-2 embedding and those of its k=3 nearest training markers, where a small distance reflects close analogues among the pretrained markers (dense local support) and a large distance an isolated marker with few close neighbours (sparse support). We then relate this distance to the achieved zero-shot Pearson correlation, repeating the analysis on the held-out IMC dataset of Rigamonti et al.^8^ and the CODEX dataset of Phillips et al.^44^ (Supplementary Fig. 15).

#### Cell-level benchmarks

Previous work on IMC image-based learning^26^ fails to decode cell types at a cellular scale. By contrast, VirTues’ cell summary tokens capture the tissue at the scale of individual cells. We test VirTues’ ability to capture biologically meaningful signals at this cell scale through cell phenotype classification experiments. This evaluation covers six datasets^2,4,6,42,44,48^, with two levels of class granularity considered for refs. ^2,42^. Further, to assess the robustness of the representations, we probed VirTues’ ability to transfer cell phenotypes learned from one labelled dataset to another dataset with only partially overlapping marker panels. For this, we used as source and target datasets the data from Cords et al.^42^ and Rigamonti et al.^8^, respectively, both measuring non-small cell lung cancer (Supplementary Fig. 16).

For the cell phenotype classification task, we performed linear probing using a logistic regression model with an L-BGFS solver and L2-regularization with coefficient λ=1.0. This ensures the evaluation focuses on the quality of the learned representations rather than the complexity or configuration of the classifier. The linear probe is applied to all cell summary tokens of the respective dataset, using the same patient-level train–test split as used during pretraining. We avoid re-sampling strategies as applying them solely to the training set did not yield noticeable performance improvements. The cell labels are taken from the originally published datasets, where they were typically assigned through an expert-guided combination of gating, clustering and machine learning methods applied to the tabular single-cell data. We note that each of these steps can introduce errors or uncertainties and we treat these annotations therefore as imperfect ground truth, which necessarily caps achievable performance (Extended Data Fig. 5). All compared methods are trained and evaluated on the same labels, so this limitation does not bias the comparative methodological conclusions. For the data from Danenberg et al.^6^, we consolidate the highly nuanced phenotypes provided by the authors into eight broad categories. Similarly, we group the phenotypes from Hoch et al.^48^ into 6 and from Phillips et al.^44^ into 11 classes. For the data from Wang et al.^2^, we define 6 high-level and 19 corresponding low-level categories. The mappings to regroup the phenotypes can be found in Supplementary Tables 4–7. For data from Cords et al.^42^, we used the provided groupings of 6 high-level and 22 low-level categories. For the dataset of Lin et al.^4^, we reconstructed phenotype annotations from the study’s binary marker positivity data, following the rule-based classification procedure described by Lin et al.^4^ (in extended data figure 1d of ref. ^4^). In summary, the tasks and respective labels are as follows:

- Course cell typing of Cords et al.^42^: six classes, namely, tumour, fibroblast, immune, T cells, vessel and other.
- Fine-grained cell typing of Cords et al.^42^: 22 classes, namely, B cells, blood, CD4, CD8, collagen cancer-associated fibroblast (CAF), high endothelial venules, hypoxic tumour, IDO CAF, lymphatic, myeloid, neutrophil, normal tumour, podoplanin CAF, smooth muscle actin CAF, dividing CAF, hypoxic CAF, hypoxic tumour-like CAF, inflammatory CAF, matrix CAF, tumour-like CAF, vascular CAF and other.
- Cell typing of Danenberg et al.^6^: eight classes, namely, natural killer cells, B cells, T cells, myeloid, oestrogen receptor^+^, oestrogen receptor^−^, stromal and APC.
- Cell typing of Hoch et al.^48^: six classes, namely, tumour, lymphocytes, macrophages, stroma, T cells and other.
- Course cell typing of Wang et al.^2^: six classes, namely, stroma, immune, tumour, T cells, vessel and other.
- Fine-grained cell typing of Wang et al.^2^: 19 classes, namely, fibroblasts, dendritic/APC, macrophage, B/plasma cells, natural killer cells, neutrophils, tumour, hypoxic tumour, EMT-like tumour, DNA-damaged cells, apoptotic cells, CD4 T cells, CD8 T cells, regulatory T cells, stem-like T cells, endothelial, PD-L1^+^GZMB^+^ cells, PD-L1^+^IDO^+^ cells and major histocompatibility complex-I and II^+^ cells.
- Cell typing of Phillips et al.^44^: 11 classes, namely, tumour, epithelium, myeloid cells, stromal cells, T cells, vasculature, B cells, Langerhans cells, mast cells, nerves, neutrophils.
- Cell typing of Lin et al.^4^: 15 classes, namely, tumour, PD-L1^+^ tumour, endothelial, stroma, vascular smooth muscle cells, B cells, cytotoxic T cells, regulatory T cells, exhausted CD8 T cells, helper T cells, memory T cells, T cells, M2-like, macrophage and other.

We report the *F*_1_ score per class as well as the macro-averaged *F*_1_ score and benchmark against the two baselines, CA-MAE^31^ and KRONOS^35^. We estimate 95% confidence intervals by bootstrapping.

Concerning the analysis of whether cell type annotations remain stable when measured markers are replaced by reconstructed (virtual) markers (Supplementary Fig. 16), we proceeded as follows. For data from Danenberg et al.^6^, we sequentially removed canonical B cell markers (first CD20, then CD20 and CD38), reconstructed the missing channels with VirTues, and re-ran cell phenotyping on the resulting partially virtual image. For the dataset of Cords et al.^42^, we performed an analogous analysis for T cell identification by progressively excluding key T cell markers of increasing difficulty (CD3E only; CD3E^+^CD4; CD3E^+^CD4^+^CD45RA; CD3E^+^CD4^+^CD45RA^+^CD8), reconstructing the excluded channels using VirTues, and re-running phenotyping.

#### Cross-cohort cell segmentation and cell typing

VirTues’ patch representations map tissues, independent of their measured marker panels, into a shared representation space. This shared space enables the training and evaluation of cell instance and cell type segmentation modules on and across several cohorts. To support this, we harmonized cell label annotations across ten datasets by mapping the annotations provided by the original studies into seven shared classes: tumour, fibroblast/stroma, myeloid, CD8 T cell, CD4 T cell, B cell and vessel/endothelial. Ambiguous labels and study-specific rare cell types were assigned to an additional eighth class, ‘unknown’, which is excluded from inference and evaluation. The full mapping schema used for this harmonization is provided in Supplementary Table 8.

We evaluate cell instance segmentation and cell typing performance independently of each other under an out-of-cohort setting. For each evaluated dataset, we train a separate segmentation module while explicitly excluding that dataset from the training data.

For cell instance segmentation, we report *F*_1_ scores across several IoU thresholds. The IoU between a predicted and a ground-truth cell instance is defined as the ratio of the number of intersecting pixels to the number of pixels in their union. For a given IoU threshold τ, predicted and ground-truth instances are matched greedily in a one-to-one manner: a predicted instance is counted as a true positive if it achieves ${\rm{IoU}}\ge \tau$ with a unique ground-truth instance. Predictions that cannot be matched are counted as false positives, while unmatched ground-truth instances are counted as false negatives. We compare performance with three specialized cell instance segmentation models: Cellpose^49^, InstanSeg^50^ and StarDist^51^.

For the evaluation of the semantic cell type masks, the predicted masks are combined with the ground-truth cell instance masks, and per-cell type predictions are obtained by majority voting. We then report class-wise and macro-averaged *F*_1_ scores including 95% confidence intervals estimated by bootstrapping. For datasets containing only a subset of the seven cell type classes, the macro average *F*_1_ score is computed only over the classes present in the dataset. As baselines, we compare against two specialized cell annotation methods: MAPS^54^ and Astir^55^. Both methods operate on tabular mean marker abundances extracted from multiplexed images using the ground-truth cell instance masks. MAPS^54^ requires a fixed and shared marker panel across training and evaluation datasets. To satisfy this requirement while maintaining the out-of-cohort evaluation setting, each evaluated dataset is paired with a training dataset selected according to (1) matching organ of origin, where possible and (2) maximizing the number of shared markers. The resulting dataset pairings are listed in Supplementary Table 9. In addition, features are *z*-score standardized to mitigate batch effects. By contrast, Astir^55^ is a completely label-free annotation tool requiring only lists of canonical markers per target cell type and dataset. These marker lists are provided in Supplementary Table 10. To ensure a fair comparison, we disabled the prediction of Astir-specific classes ‘Unknown’ and ‘Other’. We then apply Astir^55^ independently to each dataset using arcsinh-transformed mean marker intensities.

#### Tissue structure-based risk stratification

The dataset of Danenberg et al.^6^, derived from the METABRIC study^57^, provides detailed survival data that enables us to assess the ability of VirTues to capture multi-cellular structural differences in tissues relevant to patient outcomes. Following Danenberg et al.^6^, we restrict the dataset to oestrogen receptor-positive cases, resulting in 541 tissue images. For each image, we compute cell summary tokens, which are partitioned into 120 clusters using *k*-means. Each tissue is then represented by a 120-dimensional cluster proportion vector. Based on these proportion vectors, we group tissues into four groups, again using *k*-means. Survival analysis reveals that two groups correspond to higher-risk patients and two to lower-risk patients; we merge them accordingly into high-risk and low-risk groups. More generally, the number of groups in this procedure can be tuned to balance the relative influence of structural features and survival data in the stratification process. We compute Kaplan–Meier survival curves for the resulting groups and assess their separation using a log-rank test, reporting the corresponding *P* value. To connect our stratification with known biology, we leverage the ten multi-cellular TME structures described by Danenberg et al.^6^, four of which are associated with a significantly increased or decreased hazard ratio. Specifically, we calculate the risk ratio and its 95% log-transformed Wald confidence interval for the occurrence of each structure in the high-risk group. For comparison, we construct an alternative stratification based solely on survival outcomes, a priori independent of tissue structure. In this baseline, the high-risk group consists of patients who died or were censored early, whereas the low-risk group includes those who survived or were censored late (Extended Data Fig. 6a). Under this survival-based stratification, we compute also the risk ratio for the occurrence of each TME structure (Extended Data Fig. 6b).

#### Tissue-level classification

To evaluate the ability of VirTues’ representations to capture clinically meaningful information about the tissue and the patient, we define and benchmark various tissue-level classification tasks. We derive these tasks from the clinical patient-level annotations available in the metadata of all the published datasets, where each image is treated as an independent sample. For each task, we exclude images without labels and omit classes with ambiguous or missing descriptions. We describe the task, their sample sizes and their labels below:

- Primary lung cancer tissue of Cords et al.^42^ (*n* = 1,969 images):

- Cancer subtype: adenocarcinoma, squamous cell carcinoma (restricted to these as they account for 95% of samples);
- Relapse: relapse, no relapse;
- Grade: Grade 1, Grade 2, Grade 3.

- Primary breast cancer tissue of Danenberg et al.^6^ (*n* = 688 images):

- ERBB2 status: ERBB2^+^, ERBB2^−^;
- Oestrogen receptor status: oestrogen receptor^+^, oestrogen receptor^−^;
- PAM50 subtype: normal-like, Basal, HER2, Luminal A, Luminal B;
- Grade: Grade 1, Grade 2, Grade 3.

- Primary breast cancer tissue of Wang et al.^2^ (*n* = 817 images):

- Response: pathological complete response, residual disease. Note: we restrict the analysis to the same subset of per-protocol patients as used by Wang et al.^2^, who received both chemotherapy and immunotherapy, and conditioned on samples collected either before (*n* = 356 images), during (*n* = 228 images) or after (*n* = 233 images) treatment.

For all tasks, we use gated ABMIL^58^, where the input is the set of patch-level representations belonging to the tissue. The ABMIL classifiers aggregate these representations using gated attention computed over eight heads, each with a hidden dimension of 256. The aggregated tissue representation is then projected to class logits, followed by a softmax to obtain class probabilities. We train the models using the Adam optimizer^76^ with a learning rate of 10^−4^, a batch size of 16 images and a maximum of 100 epochs. Early stopping with a patience of ten epochs is applied based on a validation loss computed over a 20% hold-out validation subset of the train split. Performance is reported as the macro-averaged *F*_1_ score for each task averaged over five runs with different random seeds, initializations and validation sets. We estimate 95% confidence intervals with respect to these five runs. As baselines, we compare against KRONOS^35^, CA-MAE^31^ and ResNet^26^. For ResNet^26^, we use niche-level rather than patch-level representations. For comparisons, *P* values are indicated using two-sided Mann–Whitney *U* tests performed over the five runs.

#### Zero-shot inference

To evaluate VirTues’ ability to generalize to new datasets unseen during pretraining, that is, to perform zero-shot inference, we adopt a leave-one-dataset-out approach. Specifically, we train separate instances of VirTues on all pretraining datasets except one, which is withheld. Each resulting model is then evaluated on the excluded dataset in downstream experiments. We consider two types of downstream task: (1) masked image reconstruction on the dataset from Rigamonti et al.^8^ using independent, marker and niche masking and (2) cell type classification on the datasets from Cords et al.^42^ (coarse labels), Hoch et al.^48^, Danenberg et al.^6^ and Wang et al.^2^ (coarse labels). For the reconstruction task, we further distinguish between markers already encountered during pretraining in other datasets and entirely new markers. For both task types, we adopt the same setup and evaluation protocol as in the preceding non-zero-shot experiments. Finally, we compare the performance of the zero-shot model instances against that of the model trained on all datasets.

#### Information retrieval

VirTues allows to retrieve patients with similar molecular tissue phenotypes. As tissues are heterogeneous mixtures of distinct microenvironmental states, we represent each patient by a small set of niche summary tokens rather than collapsing everything into a single pooled vector that can dilute clinically relevant states. Specifically, we construct the Virtual Tissues database from the data of Cords et al.^42^ by extracting the central 4 × 4 grid of crops with each crop sized 128 × 128, for each image, excluding those smaller than this grid. This choice of grid size maximizes the tissue area captured per image while minimizing the number of excluded images. Furthermore, it often eliminates empty or irrelevant corners as a side effect. Similar to tissue-level tasks, we keep those images associated with adenocarcinoma or squamous cell carcinoma. The remaining crops are embedded using VirTues or one of the baselines and the resulting self-supervised niche-level representations are stored in the database.

The database is used to retrieve tissues similar to a given reference image, measured using the 2-Wasserstein distance between sets of niche-level representations. Here we used the 2-Wasserstein distance as it provides a principled metric between empirical distributions with potentially distinct supports and remains meaningful in the small-sample regime. Given the niche-level representations ${\bf{a}},{\bf{b}}\in {{\mathbb{R}}}^{N\times {d}_{{\rm{model}}}}$ for two tissues, the 2-Wasserstein distance computes as

$${W}_{2}({\bf{a}},{\bf{b}})\,=\,{\left(\mathop{\min }\limits_{\pi \in \varGamma }\mathop{\sum }\limits_{i,j=1}^{N}{\pi }_{{ij}}{\parallel {{\bf{a}}}_{i}-{{\bf{b}}}_{j}\parallel }_{2}^{2}\right)}^{\frac{1}{2}},$$

where $\varGamma =\{\pi \in {{\mathbb{R}}}_{\ge 0}^{N\times N}| \pi \vec{1}=\vec{1}/N\,{\rm{and}}\,{\pi }^{T}\vec{1}=\vec{1}/N\}$ is the set of admissible transport plans (couplings) between the two tissues, that is, the joint distributions whose marginals are both uniform over the N niche tokens. Each entry ${\pi }_{{ij}}$ specifies the mass transported from niche token i of ${\bf{a}}$ to niche token j of ${\bf{b}}$. For a given reference image, we identify the closest matches based on this distance metric.

To evaluate the efficacy of the retrieval mechanism, we perform a quantitative comparison across different embedding methods. Specifically, we compute two complementary distance metrics between each reference image and its closest retrieved match, and compare their average values against those from randomized retrieval. The first metric captures cell type composition by calculating the proportions of coarse cell types within each image (using published annotations) and measuring the L1 distance between the resulting proportion vectors. The second metric reflects molecular tissue composition by representing each image as a set of pixel vectors and computing the sliced Wasserstein distance^68^ between these sets. Formally, for two tissue images with pixels $x,y\in {{\mathbb{R}}}^{P\times C}$, where P denotes the number of pixels per image, the sliced Wasserstein distance is defined as

$$\begin{array}{r}S{W}_{2}(x,y)={({\int }_{{{\mathbb{S}}}^{c-1}}{W}_{2}{({{\rm{proj}}}_{\theta }(x),{{\rm{proj}}}_{\theta }(y))}^{2}d\theta )}^{1/2},\end{array}$$

where ${{\rm{proj}}}_{\theta }$ denotes the projection of each row onto the unit vector θ. We used the sliced Wasserstein distance as a computationally efficient approximation of the Wasserstein distance, given the very large number of pixels per image.

We further evaluate clinical feature matches using a two-sided McNemar test. For each clinical feature, we compare the number of correct matches among the top three results retrieved by our Wasserstein-based method against those from three randomized retrievals, and report the corresponding *P* values. Let ${n}_{1}$ denote the number of cases matched correctly by the Wasserstein-based retrieval but not by the random retrievals, and ${n}_{2}$ the number of cases matched correctly by the random retrievals but not by the Wasserstein-based retrieval. The McNemar test statistic is then defined as ${\chi }_{0}^{2}={({n}_{1}-{n}_{2})}^{2}/({n}_{1}+{n}_{2})$, which follows a ${\chi }^{2}$ distribution with 1 d.f. The *P* value is given by $\Pr ({\chi }^{2}\ge {\chi }_{0}^{2})$, with P<0.05 indicating a statistically significant difference in clinical label matching between the two retrieval methods. For all reported retrieval metrics, we estimate 95% confidence intervals using bootstrap re-sampling over the query set.

#### Quantifying treatment responses

We use the dataset of Wang et al.^2^, which contains IMC data from a cohort of breast cancer patients sampled before, during and after treatment. For many patients, samples are available at all three time points. This dataset enables us to evaluate VirTues’ capability to quantify treatment responses and to demonstrate the discovery of virtual spatial biomarkers predictive of response.

For our analysis, we restrict the cohort to patients who receive both chemotherapy and immunotherapy and had pre- and on-treatment biopsies taken, yielding a total of 68 patients. For each patient, we compute cell summary tokens from all available samples. To quantify patient-wise the strength of treatment response between two time points ${t}_{1},{t}_{2}$, we compute the entropy-regularized 2-Wasserstein distance between cell-level representations ${\bf{a}}\in {{\mathbb{R}}}^{{N}_{1}\times {d}_{{\rm{model}}}},{\bf{b}}\in {{\mathbb{R}}}^{{N}_{2}\times {d}_{{\rm{model}}}}$ for those time points, that is,

$${W}_{2}^{\varepsilon }({\bf{a}},{\bf{b}})\,=\,{\left(\mathop{\min }\limits_{\pi \in \varGamma }\mathop{\sum }\limits_{i=1}^{{N}_{1}}\mathop{\sum }\limits_{j=1}^{{N}_{2}}{\pi }_{{ij}}({\parallel {{\bf{a}}}_{i}-{{\bf{b}}}_{j}\parallel }_{2}^{2}-\varepsilon \log {\pi }_{{ij}})\right)}^{\frac{1}{2}}.$$

As regularization strength, we use $\varepsilon ={10}^{-3}$. For patients with several tissue samples at the same time point, we use the union of all cell representations. For each treatment interval, we compare treatment response strengths between responders and non-responders using two-sided Mann–Whitney *U* tests. To qualitatively compare the average response strength between responders and non-responders, we first sub-sample the set of all cell summary tokens by a factor of ten. We then compute two-dimensional UMAP embeddings of the sub-sampled tokens. For each patient and time point, we interpret the embedded tokens as a discrete empirical distribution. Within each cohort $C\in \{\mathrm{responders},\mathrm{non-responders}\}$ and for each time point t, we compute the Wasserstein barycentre of the patient-level distributions $\{{{\bf{a}}}_{i,t}|\,i\in C\}$ with a fixed support size of m=500 points. Formally, the barycentre ${{\bf{b}}}_{C,t}\in {{\mathbb{R}}}^{m\times 2}$ is obtained as

$${{\bf{b}}}_{C,t}={\rm{\arg }}\mathop{\min }\limits_{{\bf{b}}\in {{\mathbb{R}}}^{m\times 2}}\sum _{i\in C}{W}_{2}^{0}({\bf{b}},{{\bf{a}}}_{i,t})$$

To visualize temporal changes between time points ${t}_{1},{t}_{2}$ in treatment response, we represent the trajectory of each group C in the UMAP embedding space as the sequence of displacement vectors between the medians

$$\begin{array}{c}{{\boldsymbol{\Delta }}}_{C,t}={\rm{med}}({{\bf{b}}}_{C,{t}_{2}})-{\rm{med}}({{\bf{b}}}_{C,{t}_{1}}).\end{array}$$

These vectors reflect the average shift in cell state distribution between consecutive time points.

#### Identification of foundation model-based biomarkers predictive of therapy response

For the discovery of a new generation of foundation model-derived biomarkers predictive of immunotherapy response in the dataset from Wang et al.^2^ dataset, we consider only patient samples collected before treatment. To ensure comparability with the prediction performance reported by Wang et al.^2^, we restrict the analysis further to the same subset of per-protocol patients as used by Wang et al.^2^, resulting in total of 111 distinct individuals. For each patient, we compute the set of all cell summary tokens merged across their available samples. To identify predictive foundation model-based biomarkers, we apply Leiden clustering iteratively, varying the resolution parameter r over the interval [4, 5] in steps of Δr=0.05. For each clustering, we retain only clusters containing fewer than 2,000 cells. For every patient–cluster pair, we compute the proportion of cells belonging to that cluster. These proportions are then discretized into four ranks: rank 0 for absence (0% occurrence) and ranks 1–3 corresponding to the tertiles of positive proportions. To evaluate the potential of each cluster to predict response, we use the cluster rank as the predictor in a univariate logistic regression. The performance is quantified as the mean AUROC obtained from stratified fourfold cross-validation repeated ten times. For each cluster, we also calculate the risk ratio of treatment response conditional on high cluster presence (rank >1). Clusters with a risk ratio greater than 1 are classified as response clusters, whereas those with a ratio less than 1 are classified as non-response clusters. From all clusters identified across resolutions, we select the two response clusters and the two non-response clusters with the highest individual AUROC scores as the predictive virtual spatial biomarkers. We evaluate their joint predictive performance using a multivariate logistic regression model with the concatenated cluster ranks as predictors. Performance is measured as the mean AUROC from cross-validation using 100 random 75–25% stratified train–test splits. We compare these results with the similarly cross-validated AUROC scores reported for the spatial predictor system developed by Wang et al.^2^, as well as with three baselines that use, as a univariate predictor, the ratio of tumour cells to CD4^+^ T cells, CD8^+^ T cells or B cells. For the comparisons, we compute *P* values using a two-sided independent *t*-test.

For further interpretation of the identified predictive clusters, we calculate their cell-type composition (Fig. 5f) as well as relative changes in cell-type proportions (Supplementary Fig. 22). In addition, for each cell type and cluster, we compute the proportion of neighbouring cell types, separately for cells that belong to the cluster and for those that do not (Fig. 5g).

#### Cross-cohort generalization of VirTues-derived spatial biomarkers

We evaluate the robustness and translational potential of the foundation model-derived spatial biomarkers by transferring them to the independent TNBC cohort imaged by Meyer et al.^3^. Notably, the images in this cohort were not used for pretraining VirTues or for the initial identification of clusters and the analysis is fully zero-shot.

For the transfer, we train a random forest classifier for responder and non-responder signatures using cell-level representations computed from the data of Wang et al.^2^. Each classifier consists of 200 trees, uses the Gini splitting criterion, bootstrapping and is limited to a maximum depth of 16. The task for each classifier is to predict whether a given cell belongs to the respective cluster. To train each classifier, we balance the dataset by down-sampling the majority class to ensure adequate recall for the target cluster, and we hold-out 20% of the cells as a validation set to evaluate classifier performance. Using these trained classifiers, we predict cluster memberships for all cells in the dataset of Meyer et al.^3^ based on their cell-level representations. For interpretation of these transferred clusters, we compute, for each cluster, the proportions of cells belonging to immune-inflamed, excluded or cold tumours as labelled by Meyer et al.^3^.

In contrast to Wang et al.^2^, the data of Meyer et al.^3^ lack immunotherapy response annotations but include survival data. Consequently, we substitute response analysis with an evaluation of the predictive value of our cluster ranks on disease-free survival. Specifically, mirroring the original discovery procedure, we calculate, for each cluster-patient pair, the proportion of cells assigned to that cluster and discretize these proportions into four ordinal ranks. Subsequently, we compute for each patient an overall risk score R defined as the sum of response cluster ranks minus the sum of non-response cluster ranks. Based on their risk score, patients are stratified into high-risk (R>2), medium-risk ($-1\le R\le 2$) and low-risk (R<−1) groups. We calculate Kaplan–Meier survival curves for these risk groups. To validate the statistical significance of the difference between high-risk and low-risk survival curves, we compute the *P* value of a log-rank test. Further, we report the concordance index of these risk groups and compare our results against four baselines, namely, risk groups identified by Meyer et al.^3^ and risk scores derived from tertile-transformed ratios of tumour cells to CD4^+^ T cells, CD8^+^ T cells and B cells.

### Model inspection and visualization

We investigate VirTues’ interpretability in learning meaningful signals by evaluating the attention scores from the marker and spatial attention layers in the encoder. We begin by examining the attention weights learned by the first marker attention layer for an input image. We consider the post-softmax attention weights for all channels except the patch summary token, say ${\alpha }_{h,m{\prime} ,m,i,j}$ when marker $m{\prime}$ attends to marker m for head h and spatial position (i,j), and aggregate them across all heads and spatial positions. The final scores, termed as importance scores ${{\mathcal{I}}}_{m}$, can be written as

$$\begin{array}{c}{\rho }_{m,{m}^{{\prime} },h}=\sum _{i,j}{\alpha }_{h,{m}^{{\prime} },m,i,j}\\ {{\mathcal{I}}}_{m}=\sum _{{m}^{{\prime} },h}\frac{{\rho }_{m,{m}^{{\prime} },h}-\mathop{min}\limits_{{m}^{{\prime} }}{\rho }_{m,{m}^{{\prime} },h}}{\mathop{max}\limits_{{m}^{{\prime} }}{\rho }_{m,{m}^{{\prime} },h}-\mathop{min}\limits_{{m}^{{\prime} }}{\rho }_{m,{m}^{{\prime} },h}}\end{array}$$

Visualization of spatial attention typically relies on a class token. As the pretrained VirTues inherently does not have a class token, we augment VirTues with learnable channel summary tokens (one for each channel, including the patch summary token channel), which are placed spatially at the centre of the input image. We finetune the augmented model on the cancer subtype prediction task on the data from Cords et al.^42^, using the encoded channel summary token of the cell summary layer to predict the cancer subtype. To visualize attention maps, we compute the attention scores directed from the channel summary token to the patch summary tokens in the penultimate spatial attention layer and display these as a heatmap over the spatial positions.

### Computing hardware and software

We used Python (v.3.12.9) together with PyTorch (v.2.5.1, CUDA 12.1) and Flash Attention-2 (refs. ^74,102^) (v.2.7.4) as deep learning frameworks. For data collection, downstream experiments, statistical analysis and visualizations, we further used NumPy (v.2.2.4), zarr (v.3.1.5), pandas (v.2.2.3), matplotlib (v.3.10.3), seaborn (v.0.13.2), fair-esm (v.2.0.0)^18^, scikit-image^97^ (v.0.25.2), scikit-learn^103^ (v.1.5.2), scikit-survival^104^ (v.0.24.1), lifelines^105^ (v.0.30.0) and cuML^106^ (v.25.8.0), instanseg-torch^50^ (v.0.1.1), napari (v.0.5.5), wsireg (v.0.3.10) and QuPath (v.0.5.1). Model pretraining was performed on a HPC system with NVIDIA GH200 GPUs. All downstream experiments were executed on NVIDIA A100 80 GB GPUs.

### Reporting summary

Further information on research design is available in the Nature Portfolio Reporting Summary linked to this article.

## Online content

Any methods, additional references, Nature Portfolio reporting summaries, source data, extended data, supplementary information, acknowledgements, peer review information; details of author contributions and competing interests; and statements of data and code availability are available at https://doi.org/10.1038/s41586-026-10884-y.

## Supplementary information

Supplementary Information This file contains Supplementary Figs. 1–23, Tables 1–11 and Discussion.

Reporting Summary

Peer Review File

## Data Availability

All datasets used in this study were collected from public sources, except those from Rigamonti et al.^8^ and Wang et al.^2^, which were obtained from the respective authors through data access requests. The complete harmonized data corpus, including derived annotations across multiple spatial proteomics technologies, is available at GitHub (https://github.com/bunnelab/virtues#datasets) with the exception of the dataset from Wang et al.^2^ this dataset cannot be redistributed due to licensing restrictions). We list all publicly available datasets including URLs to their source repositories in Supplementary Table 11. Protein marker sequences were obtained from https://www.uniprot.org/ and the specific UniProt IDs used can be found in the harmonized data corpus. A tool to automatically download the corresponding amino acid sequences is provided with the code. Trained model weights are available at https://github.com/bunnelab/virtues#models.

## Code availability

All code was implemented in Python. Source code is available at https://github.com/bunnelab/virtues.
