# Supplementary material for: The Virtual Tissues foundation model resolves spatial proteomics across scales
Source: Nature. 2026 Aug 5;657(8132):744–54. doi: 10.1038/s41586-026-10884-y (PMC13581600; doi:10.1038/s41586-026-10884-y)
Supplement: Supplementary file 1 [file 41586_2026_10884_MOESM1_ESM.pdf]

nature portfolio

https://doi.org/10.1038/s41586-026-10884-y

Supplementary information

The Virtual Tissues foundation model resolves spatial proteomics across scales

In the format provided by the authors and unedited

SUPPLEMENTARY INFORMATION

The Virtual Tissues foundation model resolves spatial proteomics across scales

Johann Wenckstern[1,8], Eeshaan Jain[1,8], Benedikt von Querfurth[1,8], Yexiang Cheng[1,8], Kiril Vasilev[2], Matteo Pariset[2], Phil F. Cheng[3,4], Petros Liakopoulos[3,4], Olivier Michielin[3,4], Andreas Wicki[5,6], Gabriele Gut[5,6], Charlotte Bunne[1,7,9]

[1] School of Computer and Communication Sciences, EPFL, Lausanne, Switzerland,

[2] Department of Computer Science, ETH, Zurich, Switzerland,

[3] Faculty of Medicine, University of Geneva, Geneva, Switzerland,

[4] Department of Oncology, Geneva University Hospitals, Geneva, Switzerland,

[5] Department of Medical Oncology and Hematology, University Hospital Zurich, Zurich, Switzerland,

[6] University of Zurich, Faculty of Medicine, Zurich, Switzerland,

[7] Swiss Institute for Experimental Cancer Research, School of Life Sciences, EPFL, Lausanne, Switzerland.

[8] These authors contributed equally.

[9] Correspondence to: charlotte.bunne@epfl.ch.

Supplementary Figures

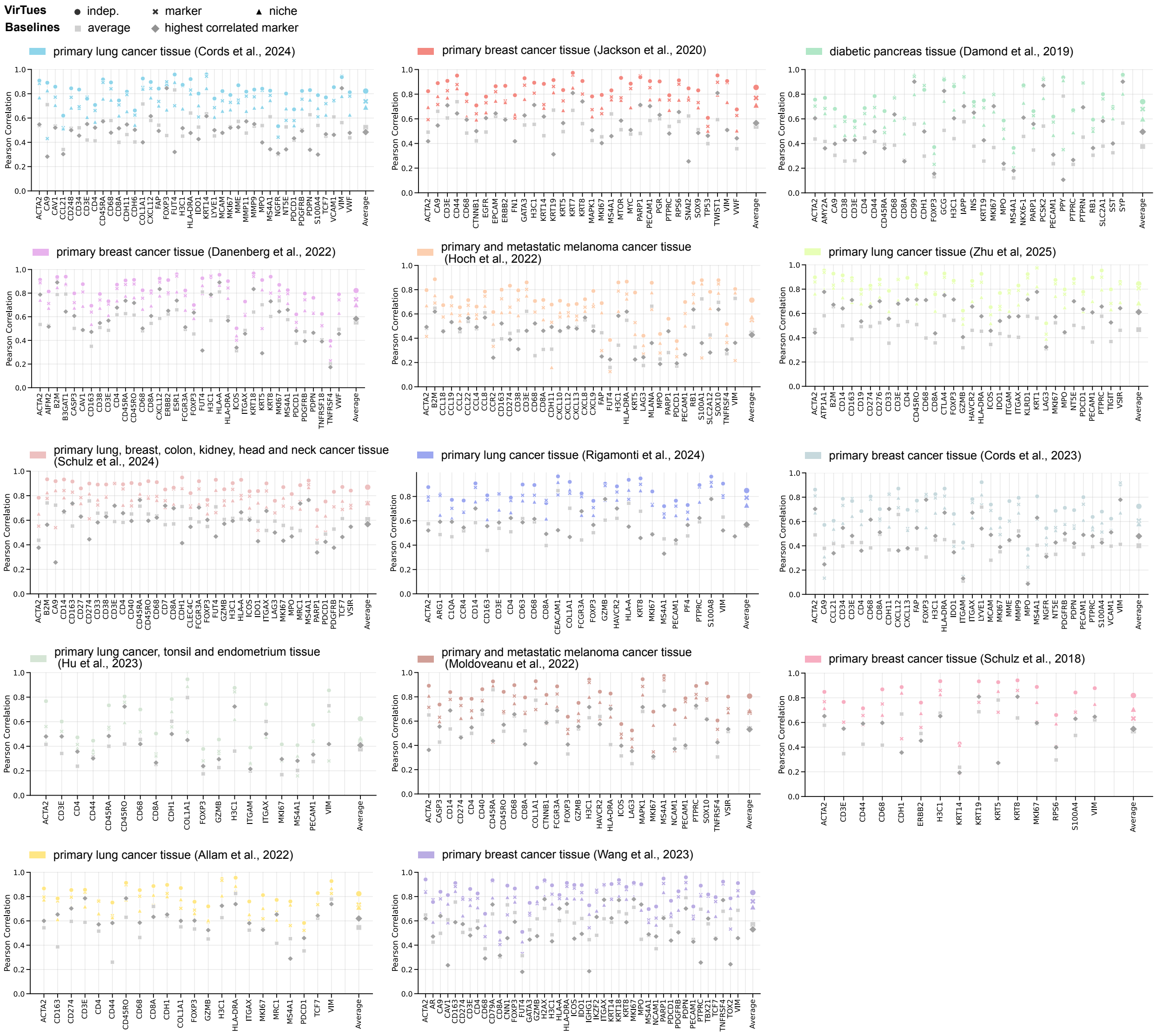

Supplementary Fig. 1: Overview of marker-wise reconstruction performance, measured by Pearson correlation, for independent (circles), marker (crosses), and niche (triangles) masking schemes. For comparison, light gray squares represent the correlation obtained by predicting the mean channel intensity of visible pixels for all masked pixels under independent masking. Dark gray diamonds indicate the correlation of each marker with the highest correlated other marker.

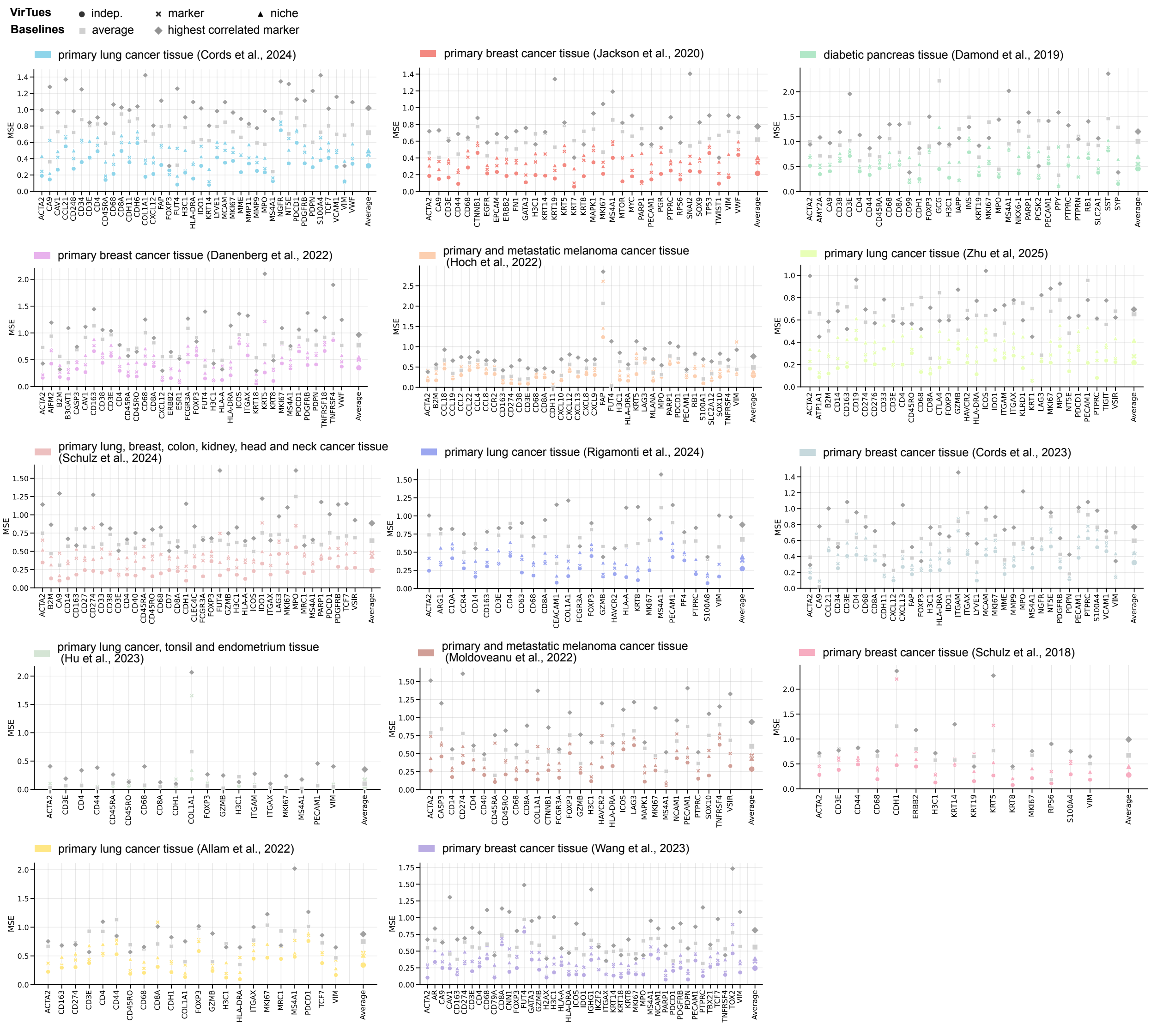

Supplementary Fig. 2: Overview of marker-wise reconstruction performance, measured by mean squared error (MSE), for independent (circles), marker (crosses), and niche (triangles) masking schemes. MSE values are computed on the standardized images. For comparison, light gray squares represent the performance obtained by predicting the mean channel intensity of visible pixels for all masked pixels under independent masking. Dark gray diamonds indicate the performance when predicting for each marker the highest correlated other marker.

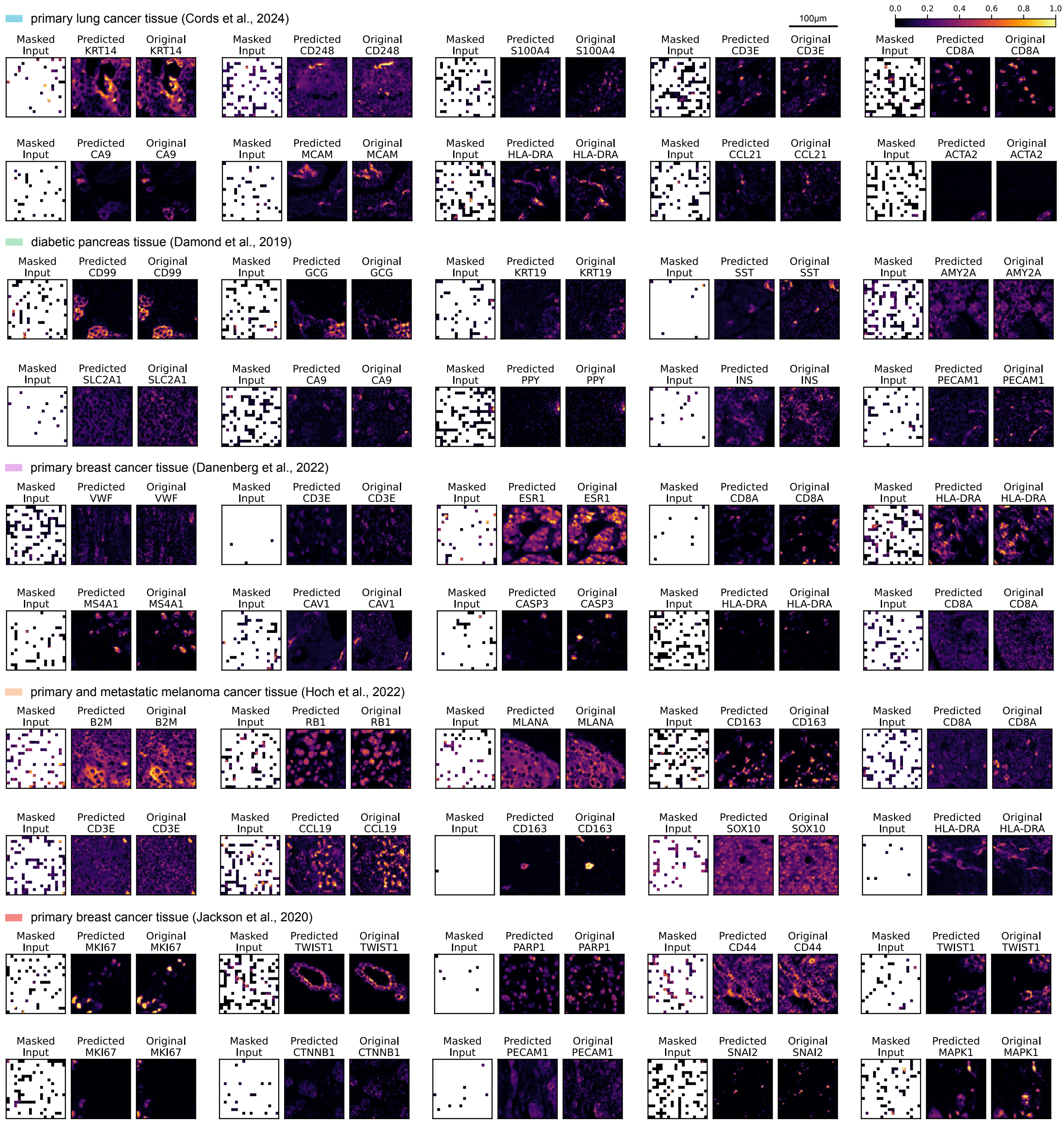

Supplementary Fig. 3: Examples of image reconstruction results using independent masking, with masking ratios between 60% and 100%, for Cords et al. [42], Damond et al. [81], Danenberg et al. [6], Hoch et al. [48] and Jackson et al. [43].

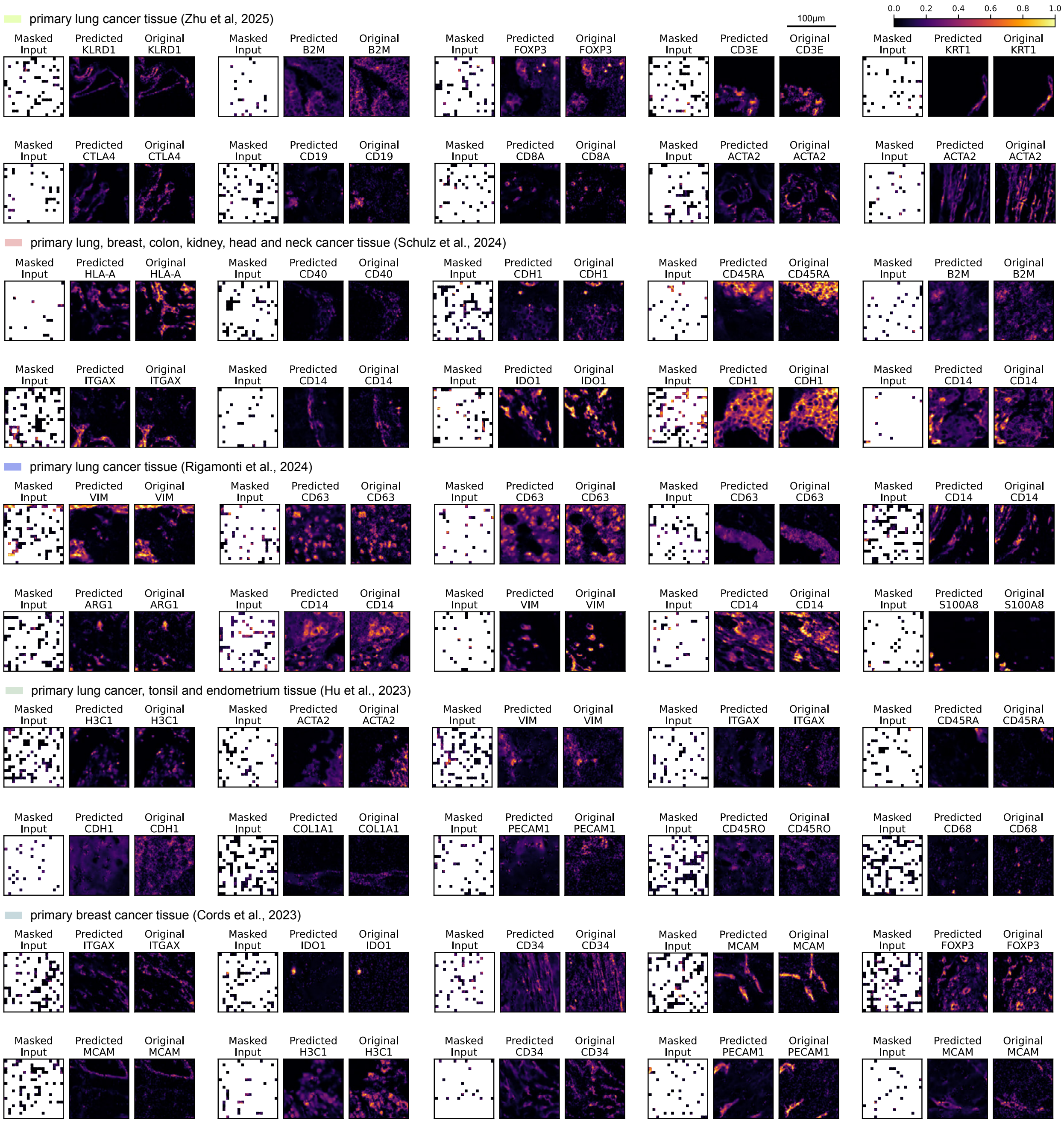

Supplementary Fig. 4: Examples of image reconstruction results using independent masking, with masking ratios between 60% and 100%, for Zhu et al.[77], Schulz et al.[53], Rigamonti et al.[8], Hu et al.[78] and Cords et al.[52].

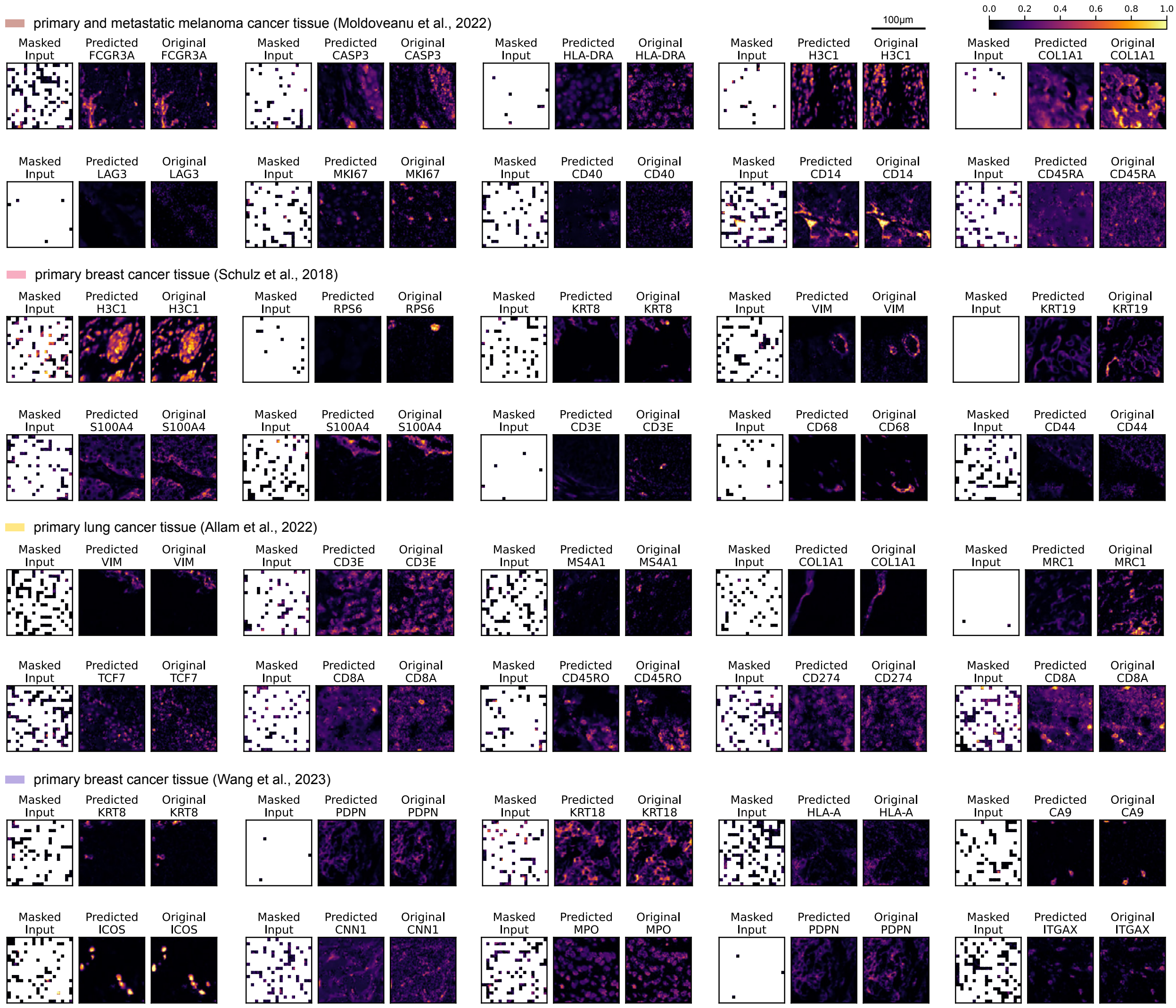

Supplementary Fig. 5: Examples of image reconstruction results using independent masking, with masking ratios between 60% and 100%, for Moldoveanu et al.[56], Schulz et al.[80], Allam et al.[79] and Wang et al.[2].

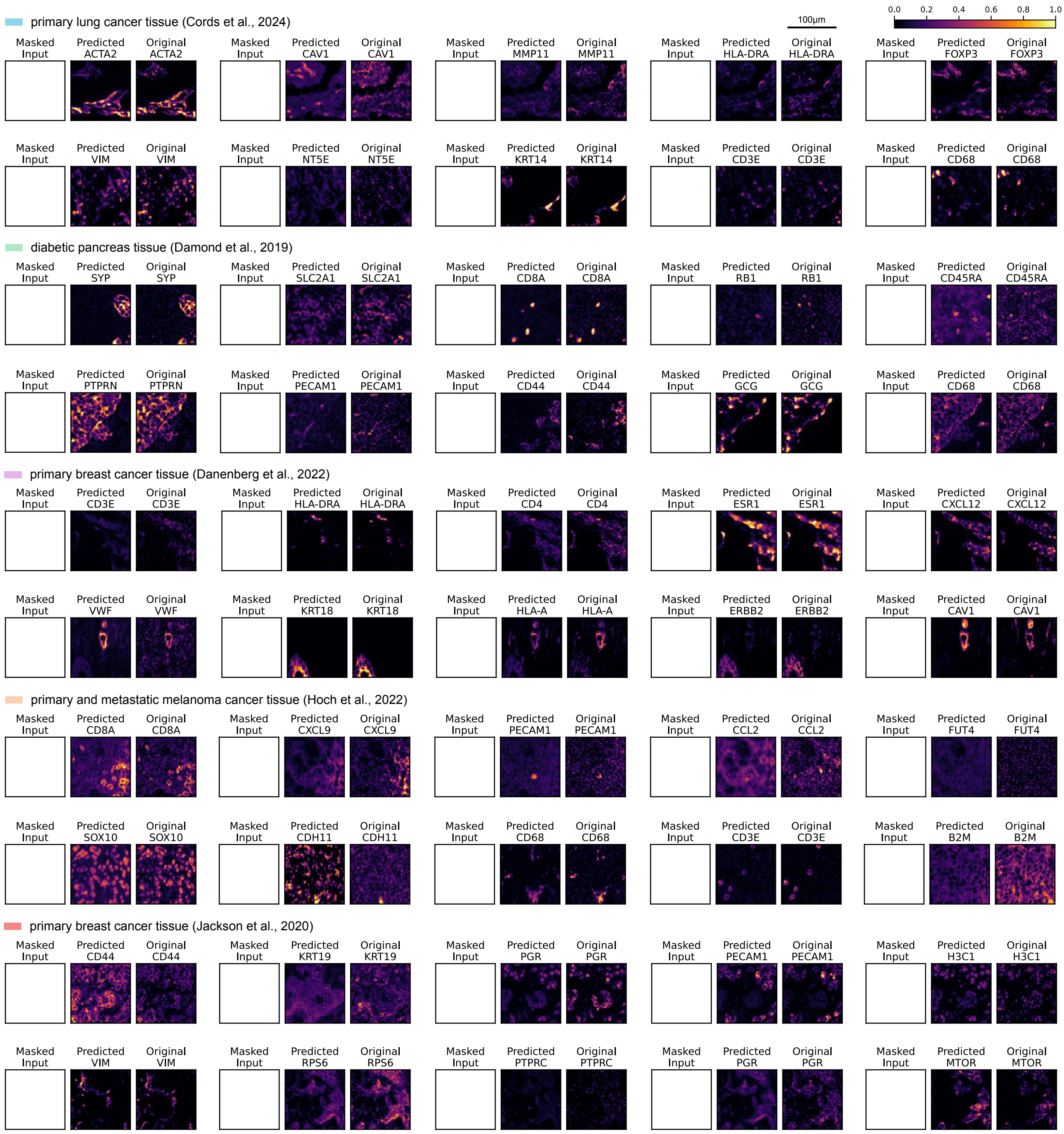

Supplementary Fig. 6: Examples of image reconstruction results after masking an entire marker for Cords et al. [42], Damond et al. [81], Danenberg et al. [6], Hoch et al. [48] and Jackson et al. [43]. Each row depicts different channels of the same tissue niche.

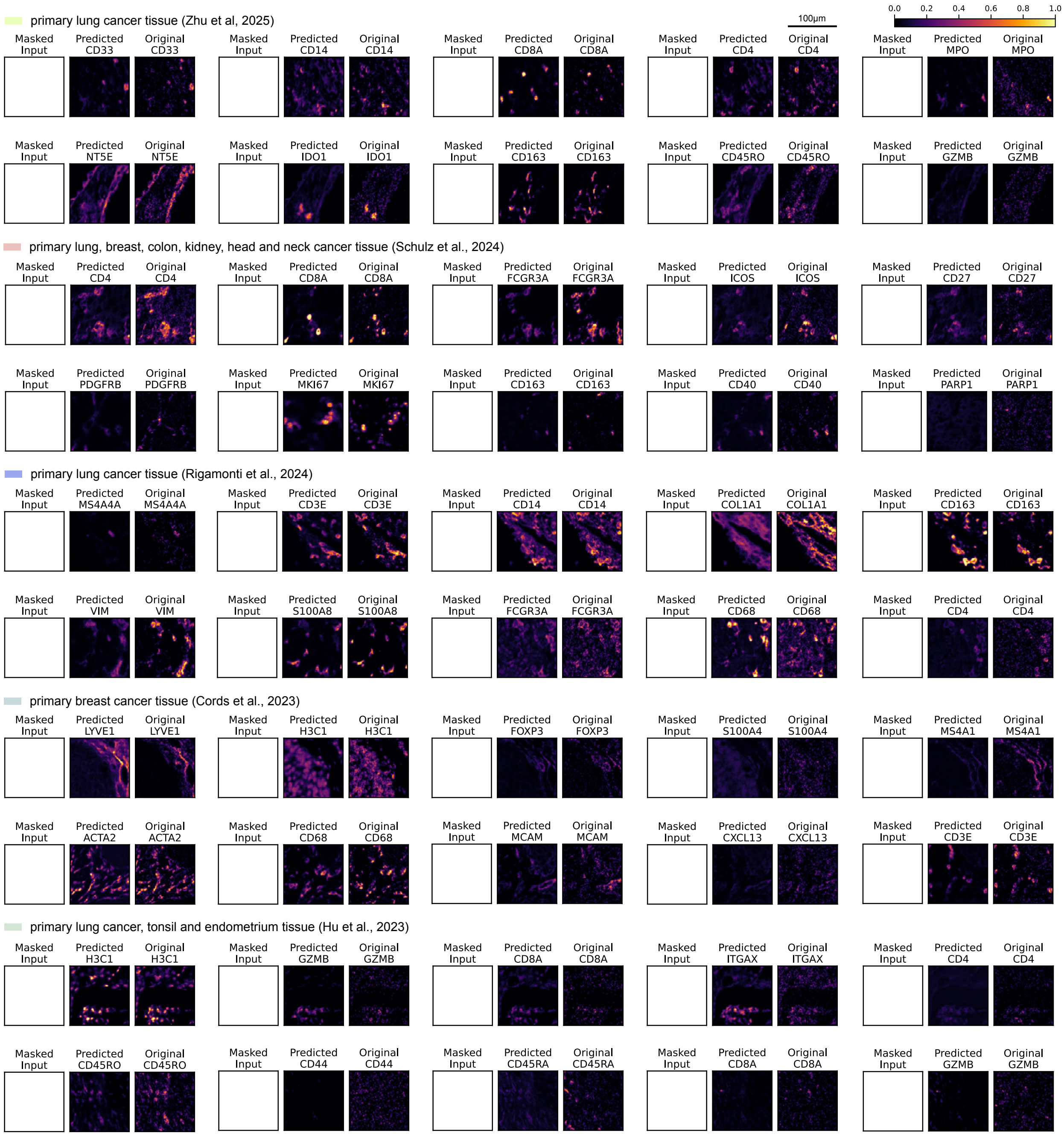

Supplementary Fig. 7: Examples of image reconstruction results after masking an entire marker for Zhu et al.[77], Schulz et al.[53], Rigamonti et al.[8], Hu et al.[78] and Cords et al.[52]. Each row depicts different channels of the same tissue niche.

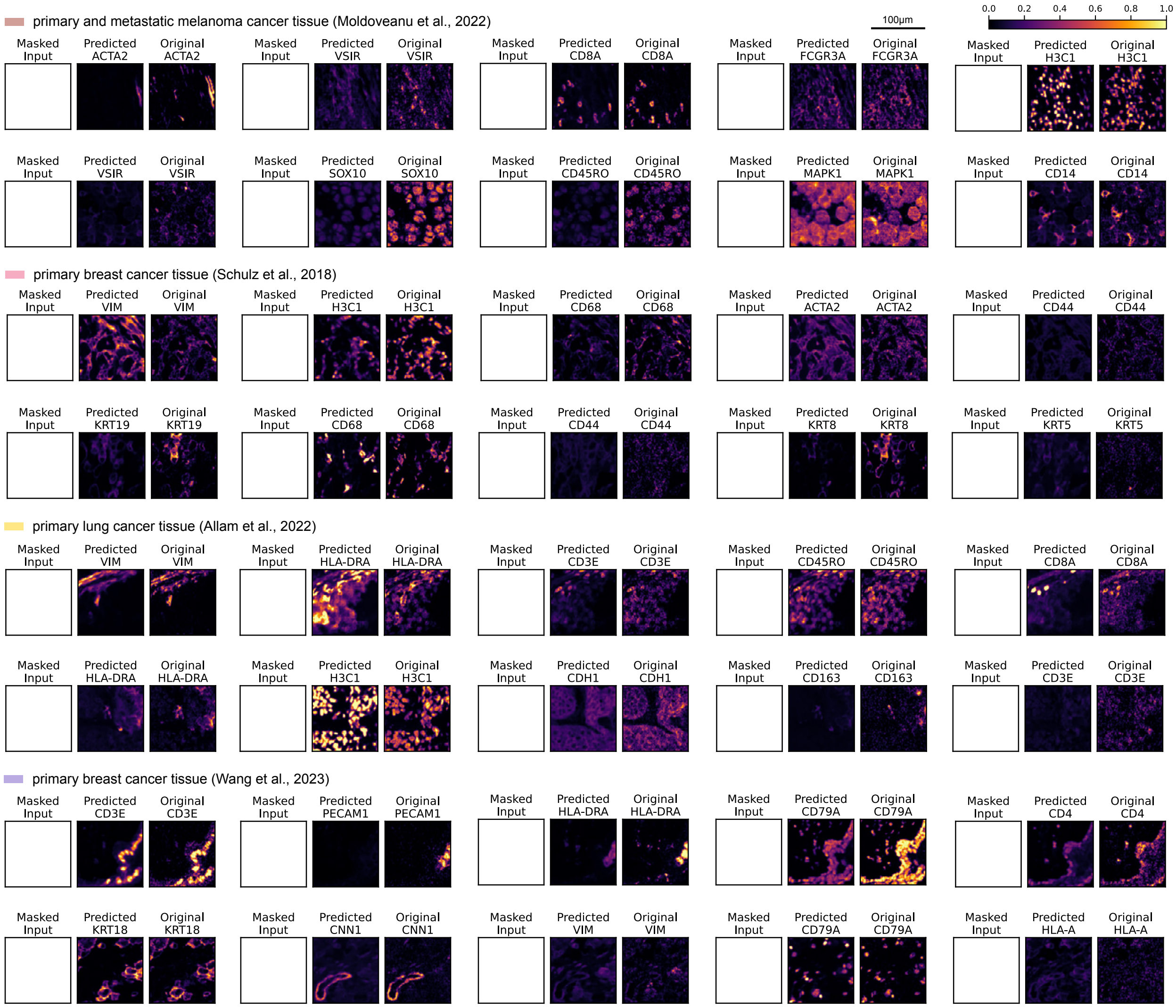

Supplementary Fig. 8: Examples of image reconstruction results after masking an entire marker for Moldoveanu et al. [56], Schulz et al. [80], Allam et al. [79] and Wang et al. [2]. Each row depicts different channels of the same tissue niche.

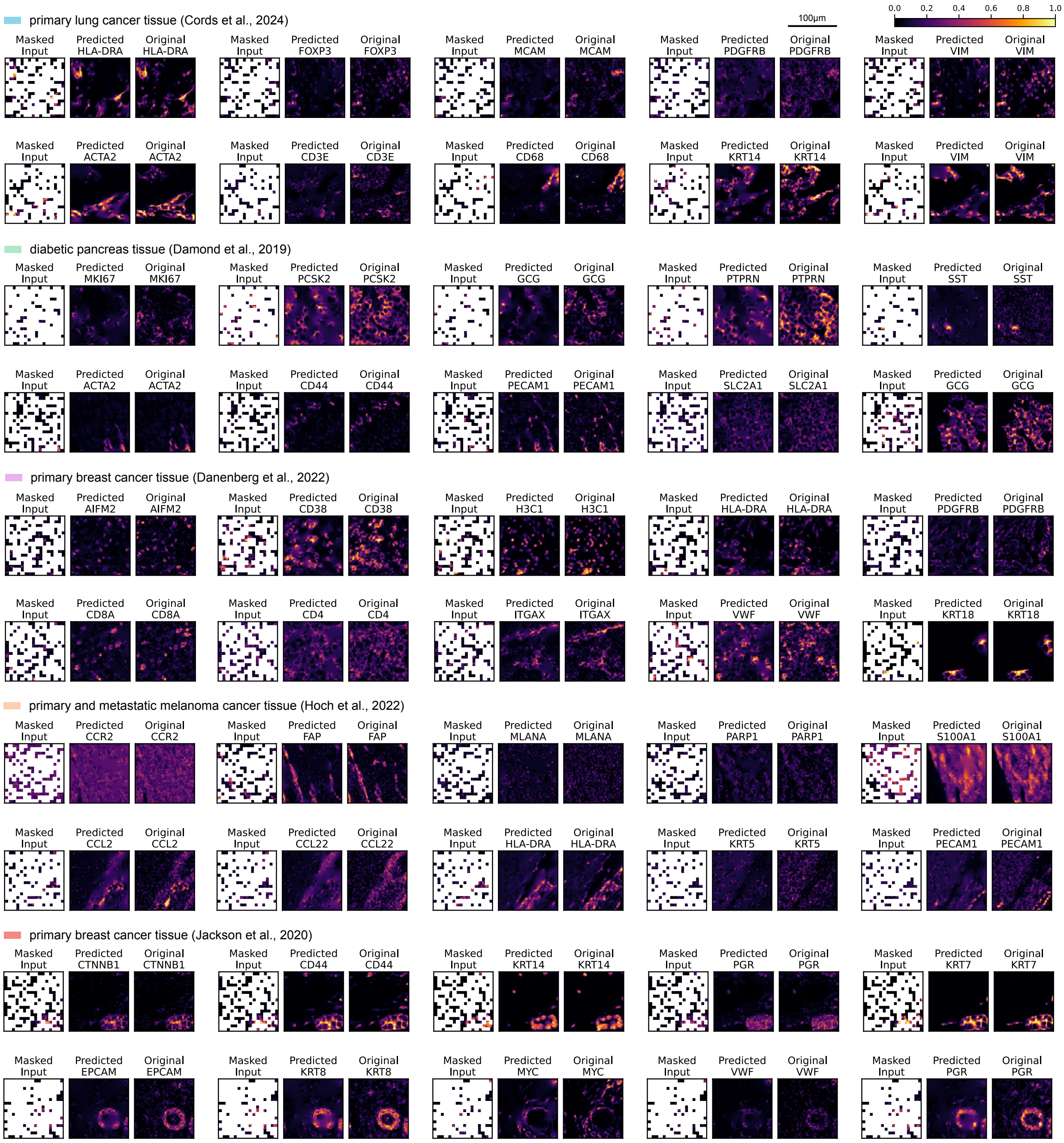

Supplementary Fig. 9: Examples of reconstruction results using niche masking, with masking ratio between 60% and 100%, for Cords et al. [42], Damond et al. [81], Danenberg et al. [6], Hoch et al. [48] and Jackson et al. [43]. Each row depicts different channels of the same tissue niche.

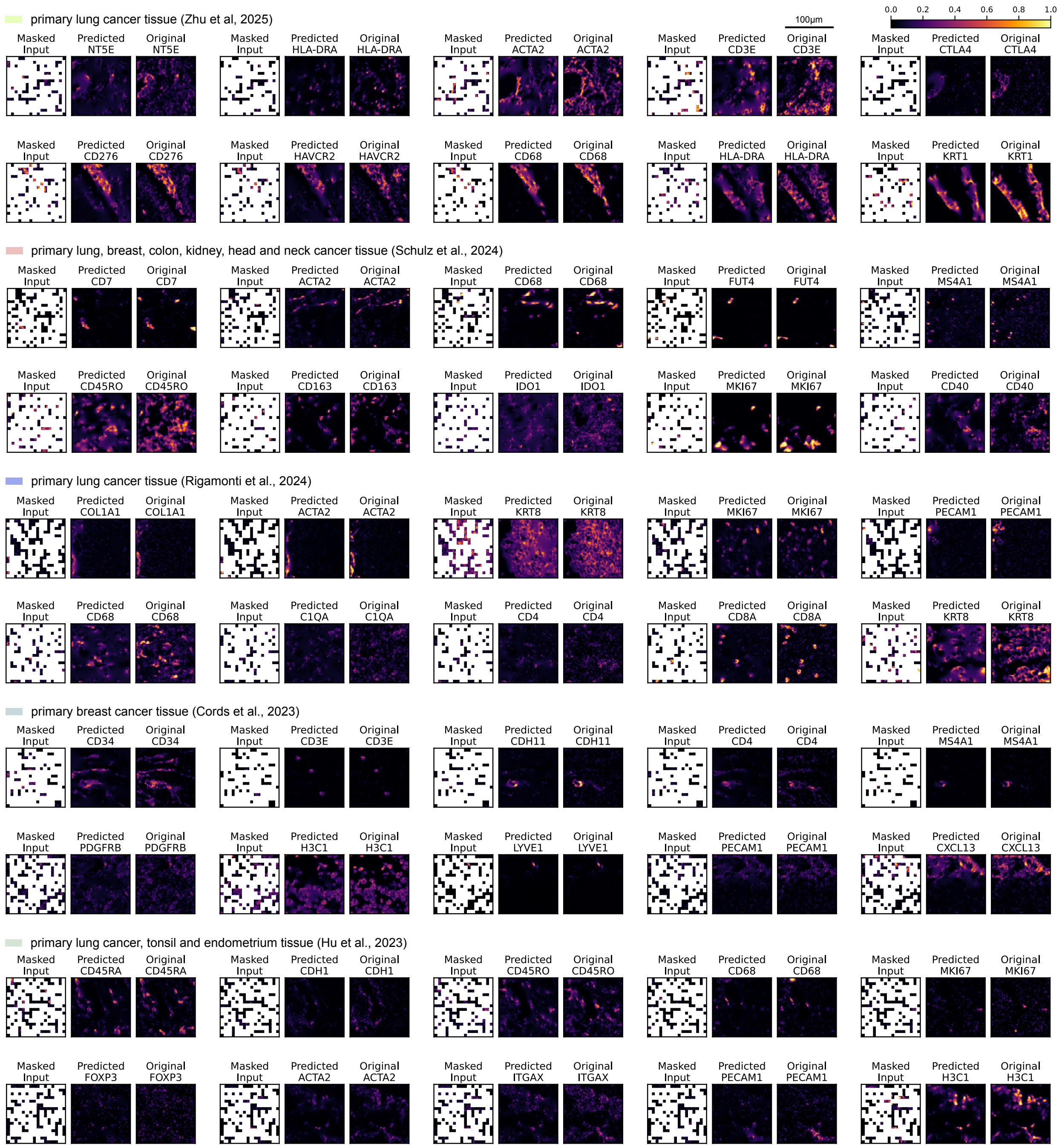

Supplementary Fig. 10: Examples of reconstruction results using niche masking, with masking ratio between 60% and 100%, for Zhu et al. [77], Schulz et al. [53], Rigamonti et al. [8], Hu et al. [78] and Cords et al. [52]. Each row depicts different channels of the same tissue niche.

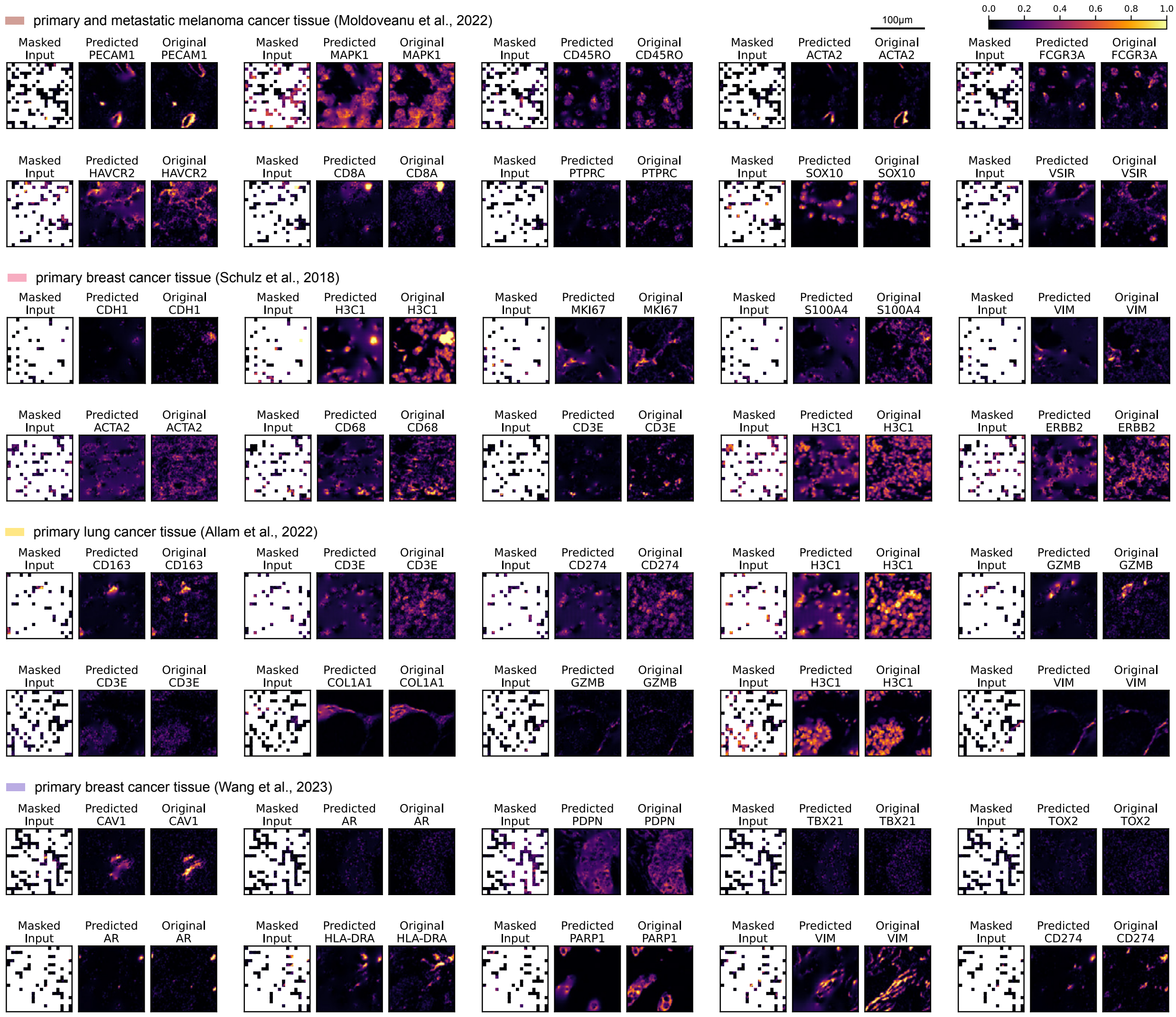

Supplementary Fig. 11: Examples of reconstruction results using niche masking, with masking ratio between 60% and 100%, for Moldoveanu et al. [56], Schulz et al. [80], Allam et al. [79] and Wang et al. [2]. Each row depicts different channels of the same tissue niche.

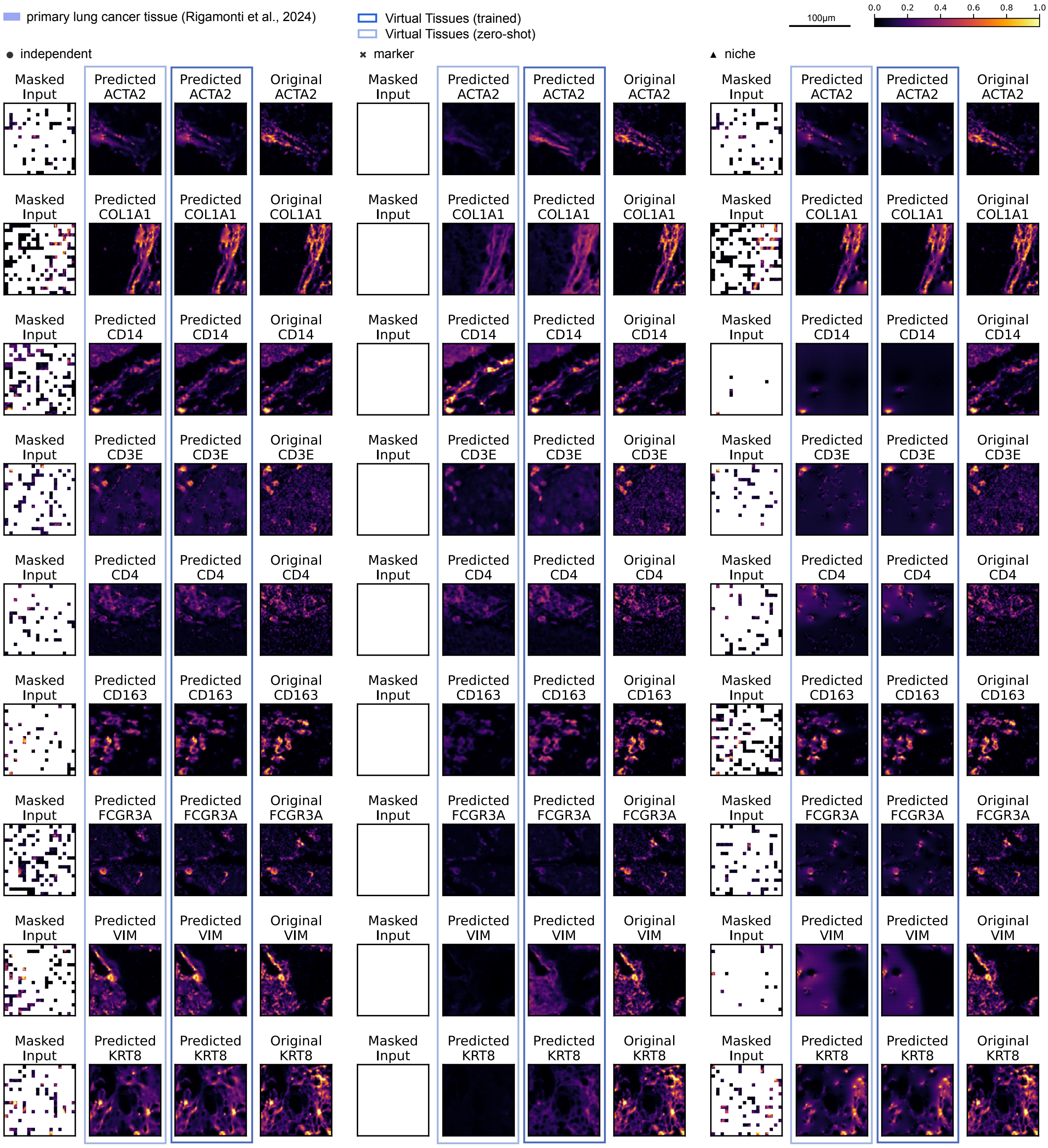

Supplementary Fig. 12: Examples of zero-shot reconstruction results using independent (left), marker (middle) and niche (right) masking for markers of Rigamonti et al. [8] observed during pretraining. For comparison, the same samples are also reconstructed using a model instance pretrained on Rigamonti et al. [8].

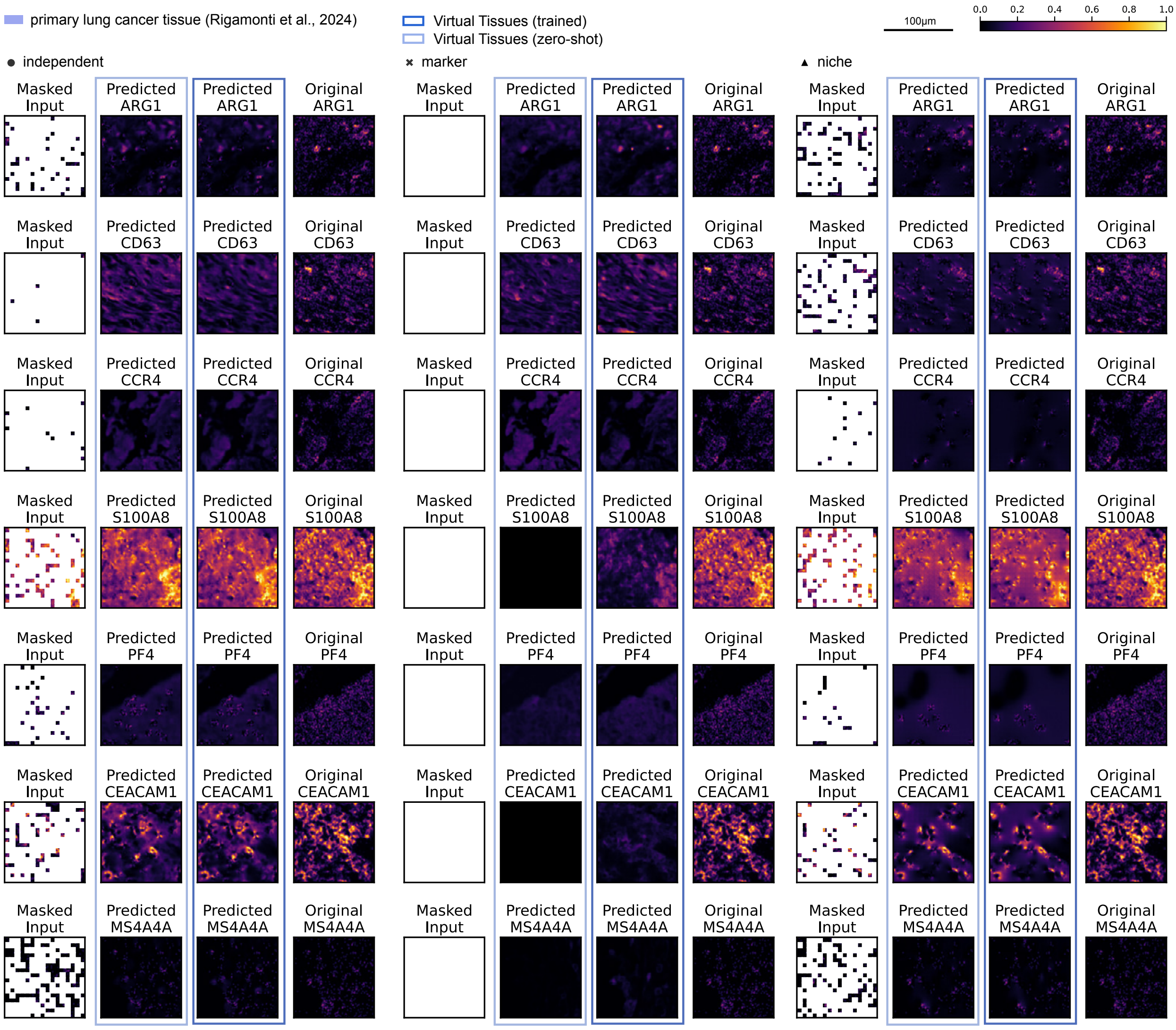

Supplementary Fig. 13: Examples of zero-shot reconstruction results using independent (left), marker (middle) and niche (right) masking for markers of Rigamonti et al. [8] not observed during pretraining. For comparison, the same samples are also reconstructed using a model instance pretrained on Rigamonti et al. [8].

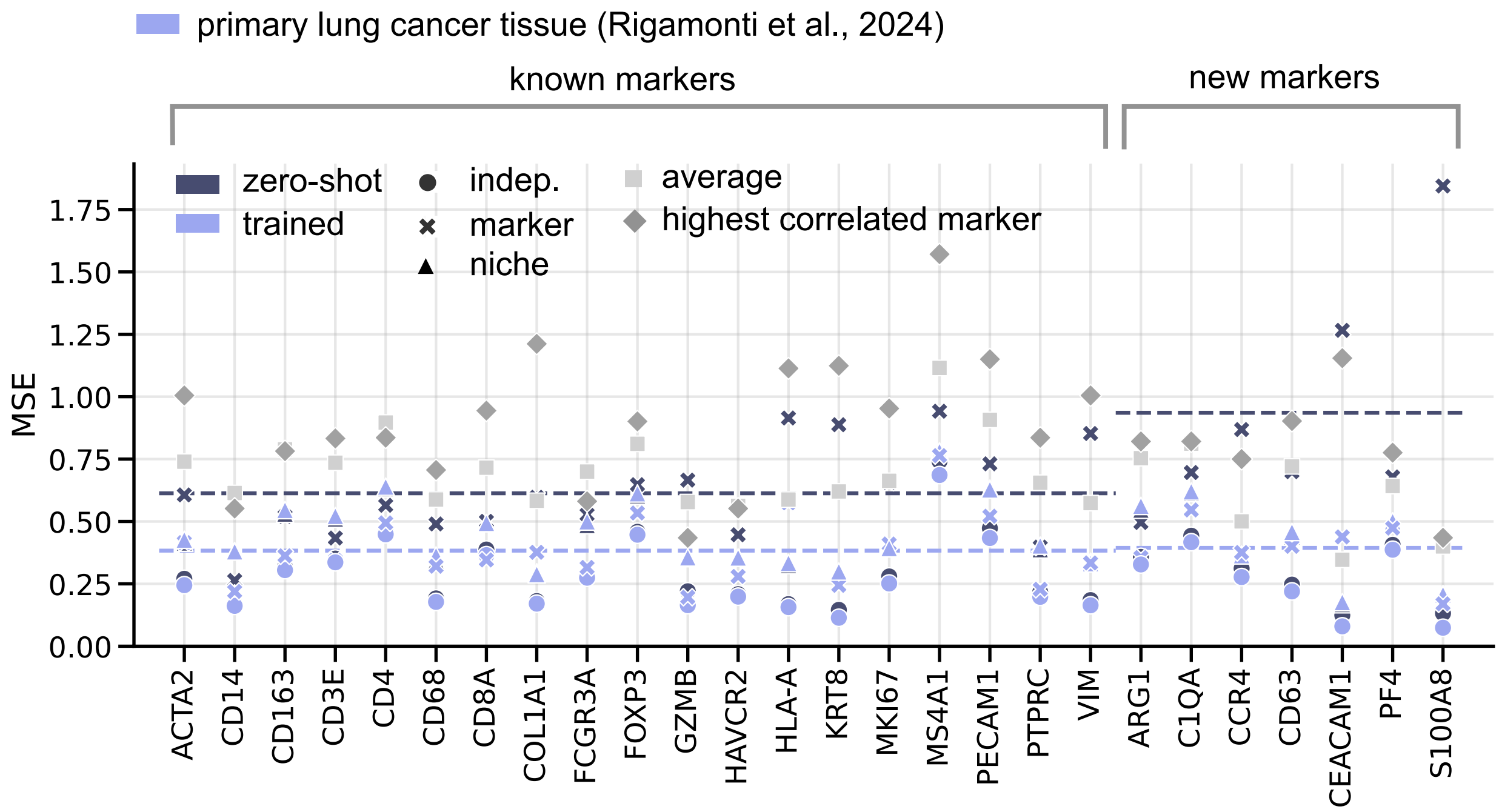

Supplementary Fig. 14: Comparison of marker-wise reconstruction performance in the zero-shot and non-zero-shot setting on Rigamonti et al.[8], measured by mean squared error (MSE). Markers are grouped into those seen during pretraining (left) and unseen ones (right). Dashed lines indicate the averages across markers using marker masking.

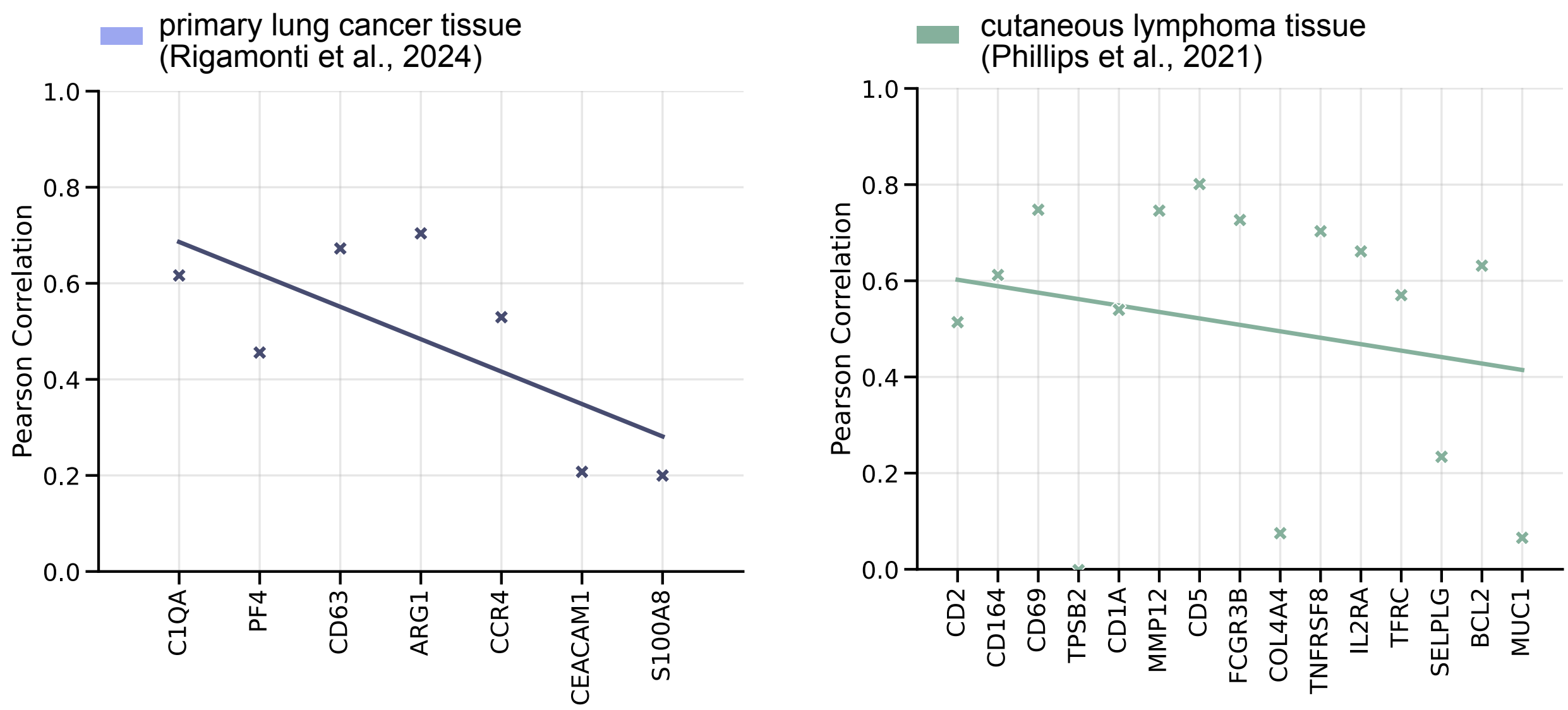

Supplementary Fig. 15: Quantitative analysis of how marker similarity relates to zero-shot reconstruction performance. Using the local density in ESM embedding space as a proxy for similarity, that is, whether a zero-shot marker lies in a "dense" neighborhood of markers seen during pretraining (many nearby proteins in sequence space) or in a "sparse" neighborhood (few or no close pretrained neighbors). For each zero-shot marker we compute the average L2 distance between its ESM embedding and those of its k nearest training markers ($k = 3$) (sorted from left to right), and relate this distance to the achieved Pearson correlation in the zero-shot setting for novel markers on the IMC dataset of Rigamonti et al.[8] (left) and the CODEX dataset of Phillips et al.[44] (right). Solid lines indicate a linear regression fit.

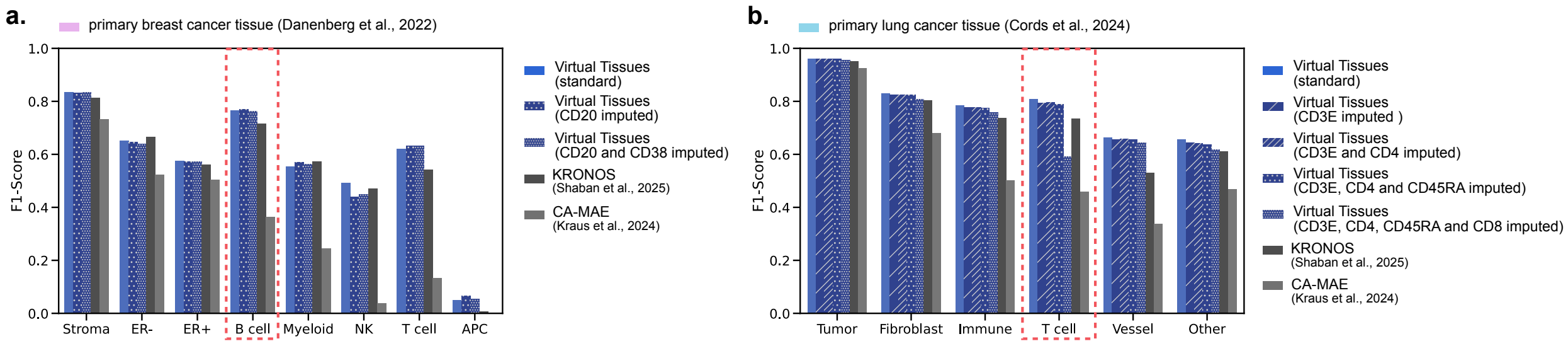

Supplementary Fig. 16: Evaluation of virtual staining for downstream cell phenotyping. VirTues is used to reconstruct subsets of markers, generating virtual stains that are incorporated into a partially virtual multiplex image for cell phenotyping. Performance is reported using class-wise F1 scores. For reference, the performance of VirTues, KRONOS, and CA-MAE using the full multiplex image, based on all real channels, is also shown. **a,** On Danenberg et al. [6] we reconstruct (i) CD20 and (ii) CD20 and CD38. Classification results for B cells, which are primarily identified by these markers, are highlighted. **b,** On Cords et al. [42] we reconstruct (i) CD3E, (ii) CD3E and CD4, (iii) CD3E, CD4 and CD45RA and (iv) CD3E, CD4, CD45RA and CD8. Classification results for T cells, which are primarily identified by these markers, are highlighted.

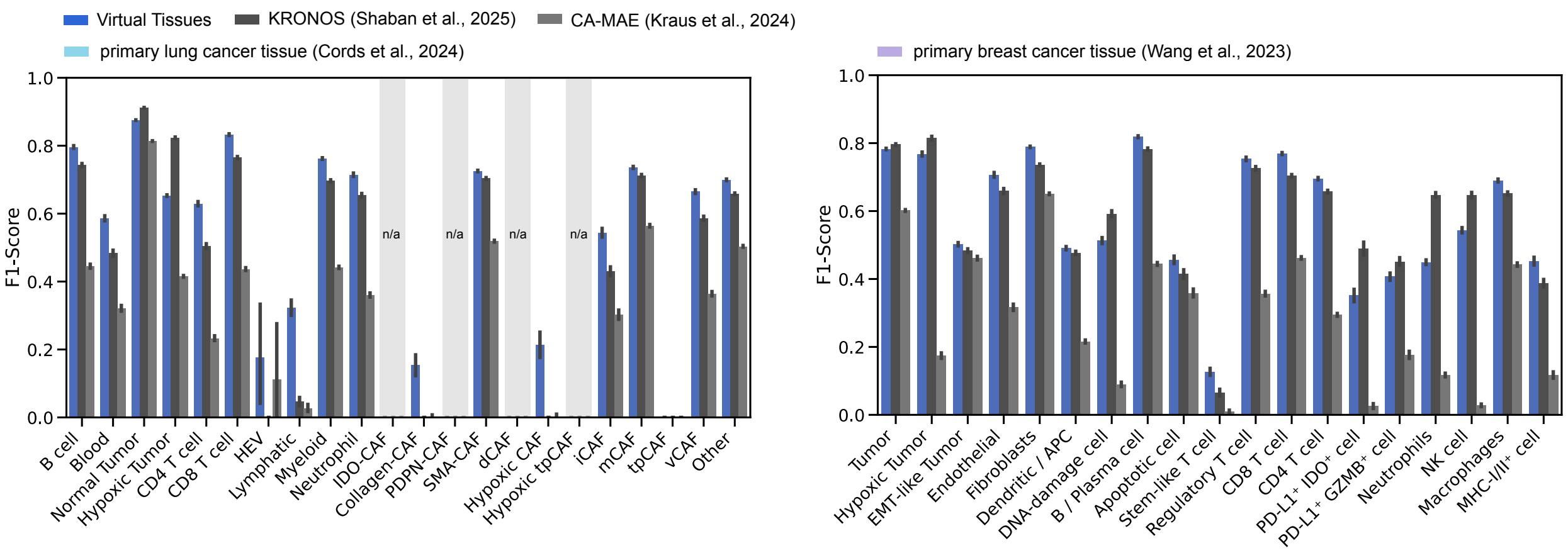

Supplementary Fig. 17: F1-scores for fine-grained cell type classification on Cords et al. [42] and Wang et al. [2]. Error bars indicate 95% confidence intervals estimated via bootstrapping. Grey panels indicate rare classes that are absent from the test split.

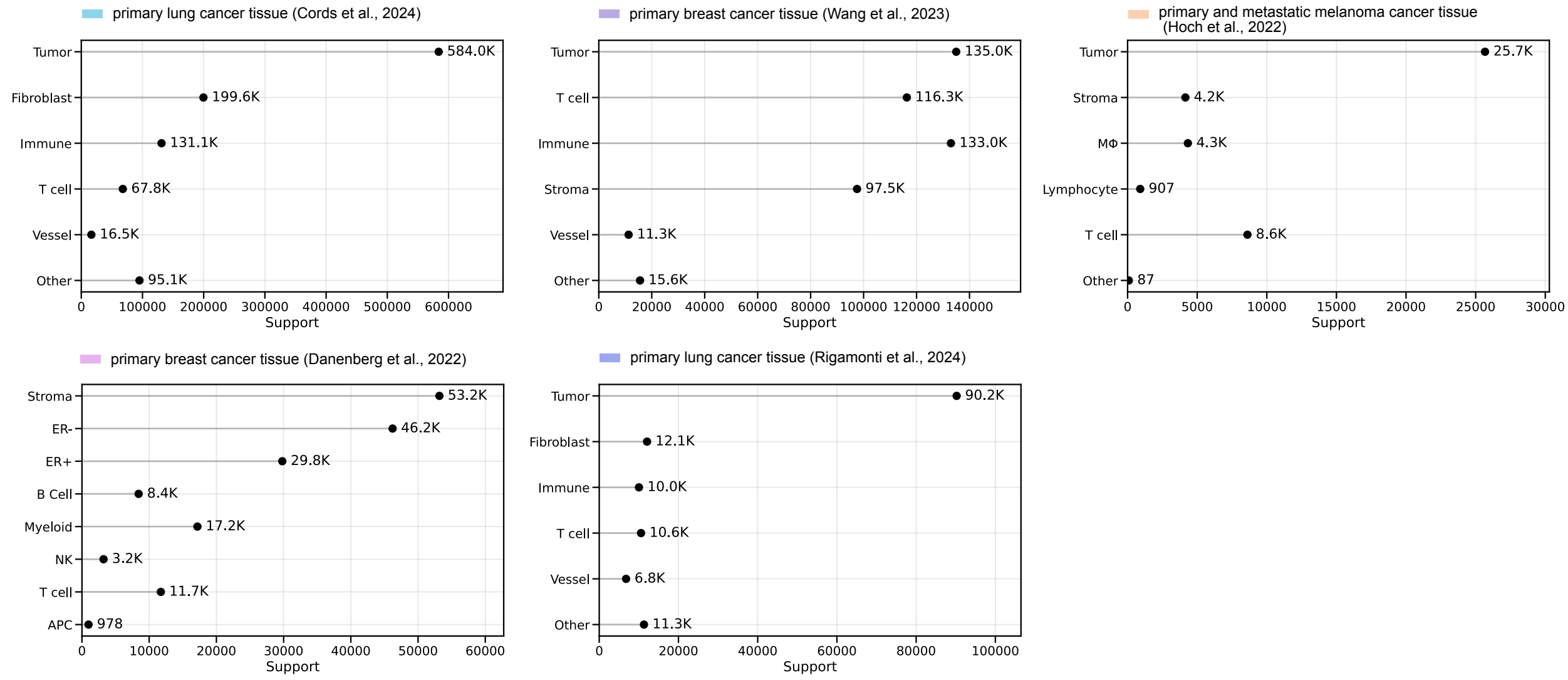

Supplementary Fig. 18: Support distribution for coarse cell types across the test sets of Cords et al.[42], Wang et al.[2], Hoch et al.[48], Danenberg et al.[6], and Rigamonti et al.[8], illustrating the imbalance in cell type representation in cell classification tasks.

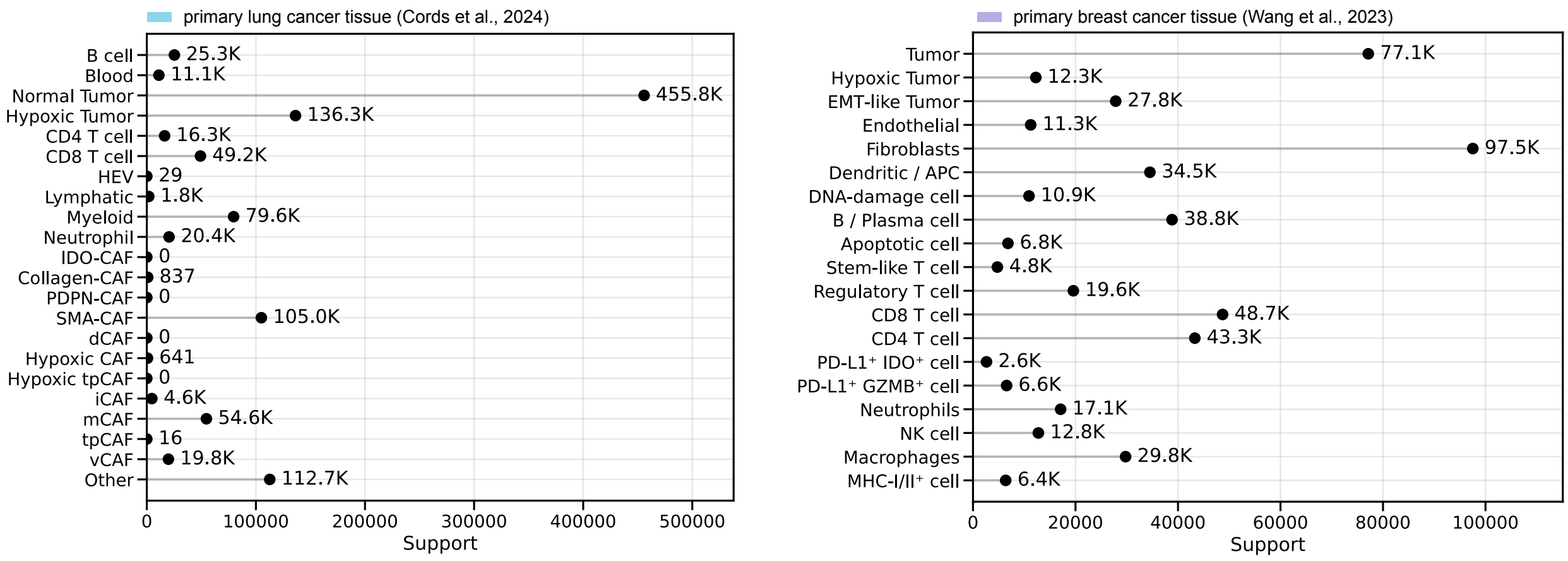

Supplementary Fig. 19: Support distribution for fine-grained cell types across the test sets of Cords et al.[42] and Wang et al.[2], highlighting the imbalance in cell type representation in cell classification tasks.

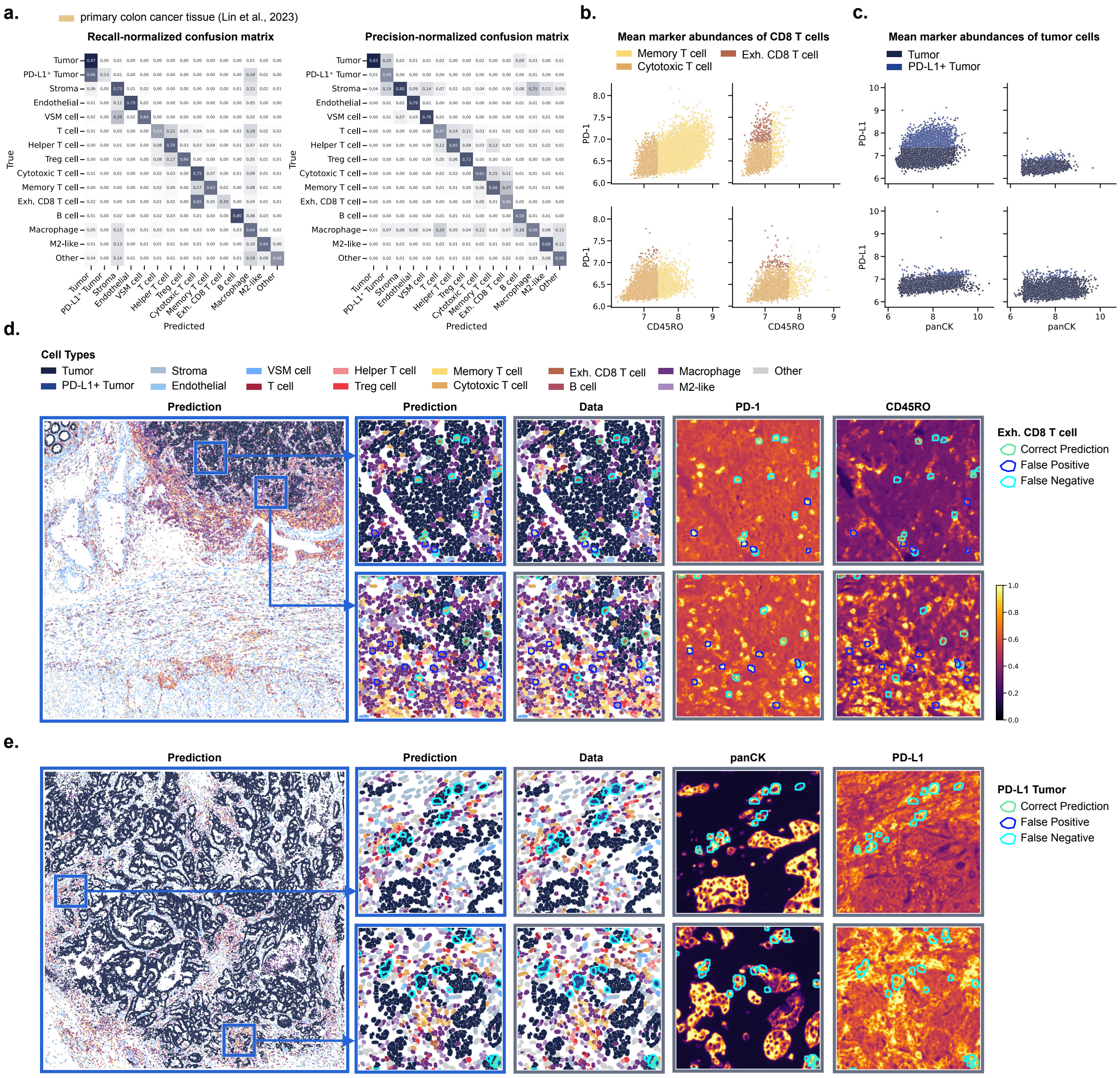

Supplementary Fig. 20: Error analysis of cross-technology cell phenotyping performance on Lin et al. (2023). **a,** Recall- and precision-normalized confusion matrices. **b,** Arcsinh-transformed mean-marker abundances for CD8 T cell subpopulations of four different tissues colored by ground truth annotations. **c,** Arcsinh-transformed mean-marker abundances for tumor cell subpopulations of four different tissues colored by ground truth annotations. **d-e,** Representative examples of predicted versus ground-truth cell type masks. **d,** Correct predictions, false positives and false negatives for exhausted CD8 T cells are visually highlighted. **e,** Correct predictions, false positives and false negatives for PD-L1 tumor cells are visually highlighted.

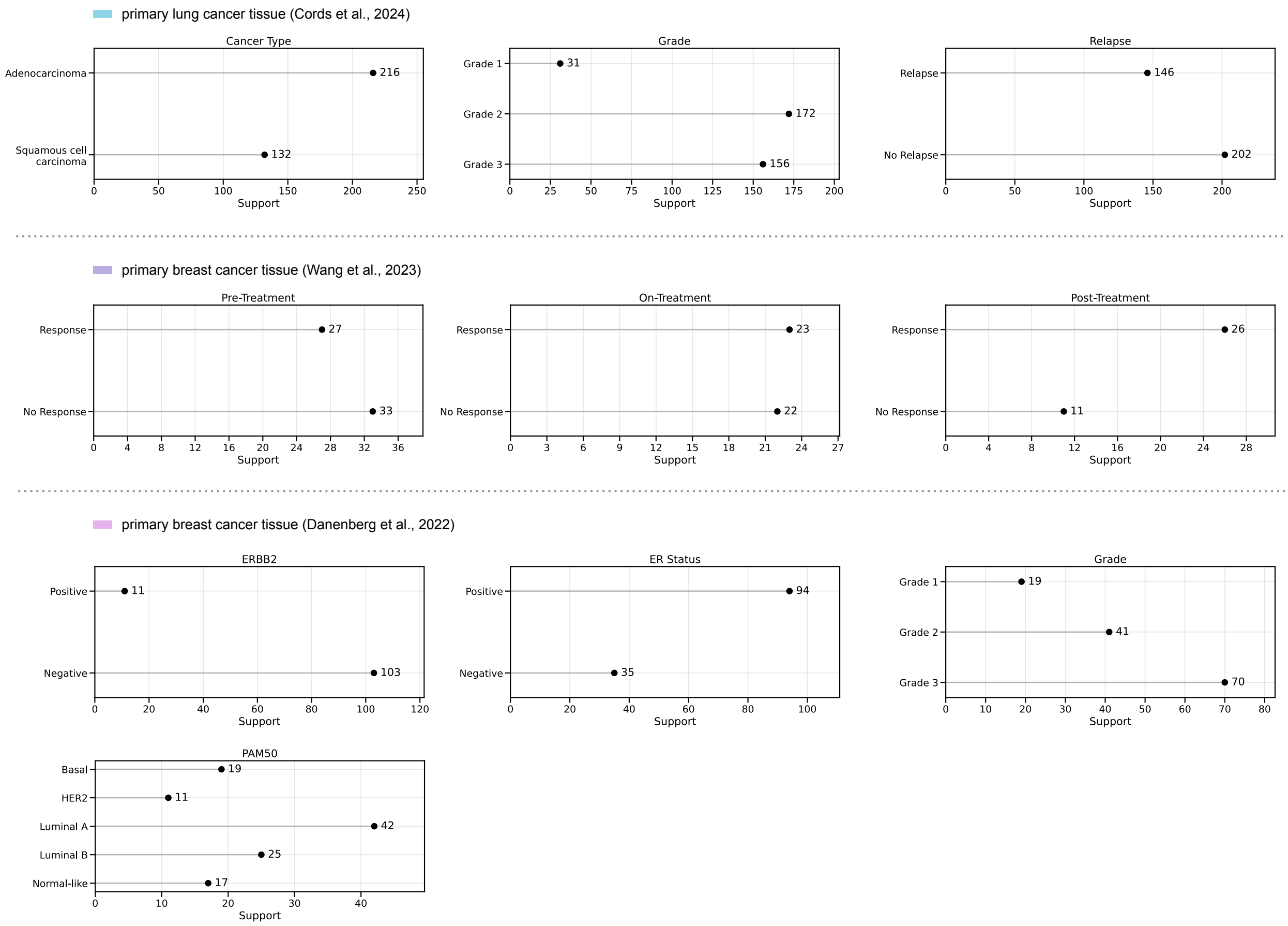

Supplementary Fig. 21: Support distribution of labels for tissue-level classification tasks across the test sets of Cords et al. [42], Wang et al. [2] and Danenberg et al. [6].

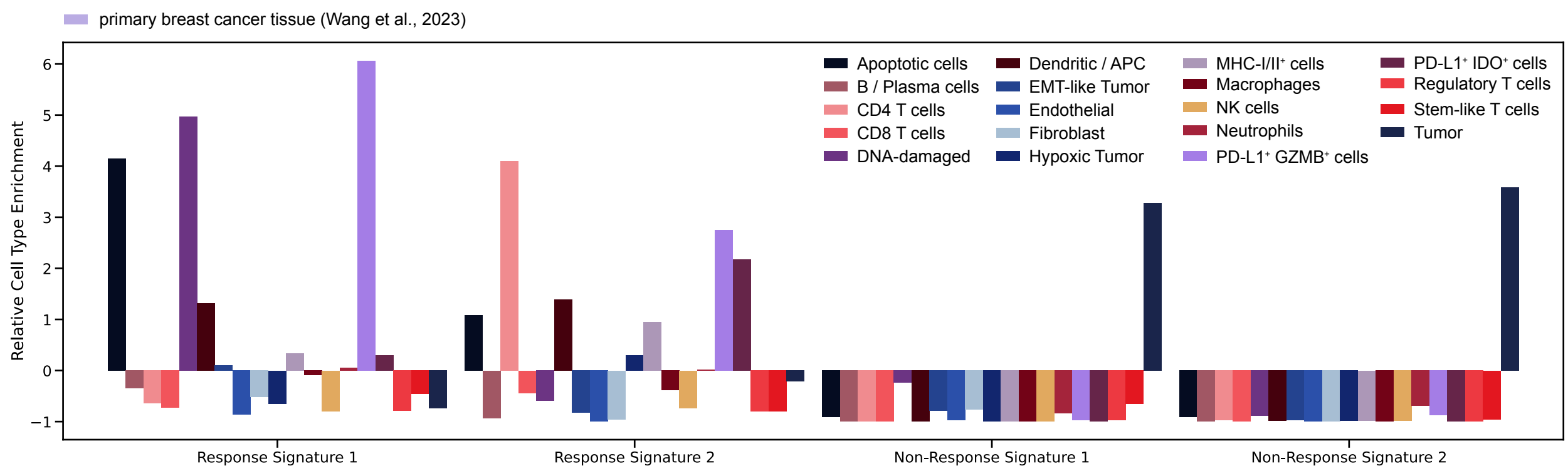

Supplementary Fig. 22: Relative enrichment of cell types in selected response and non-response clusters of Wang et al. [2] compared to overall proportions.

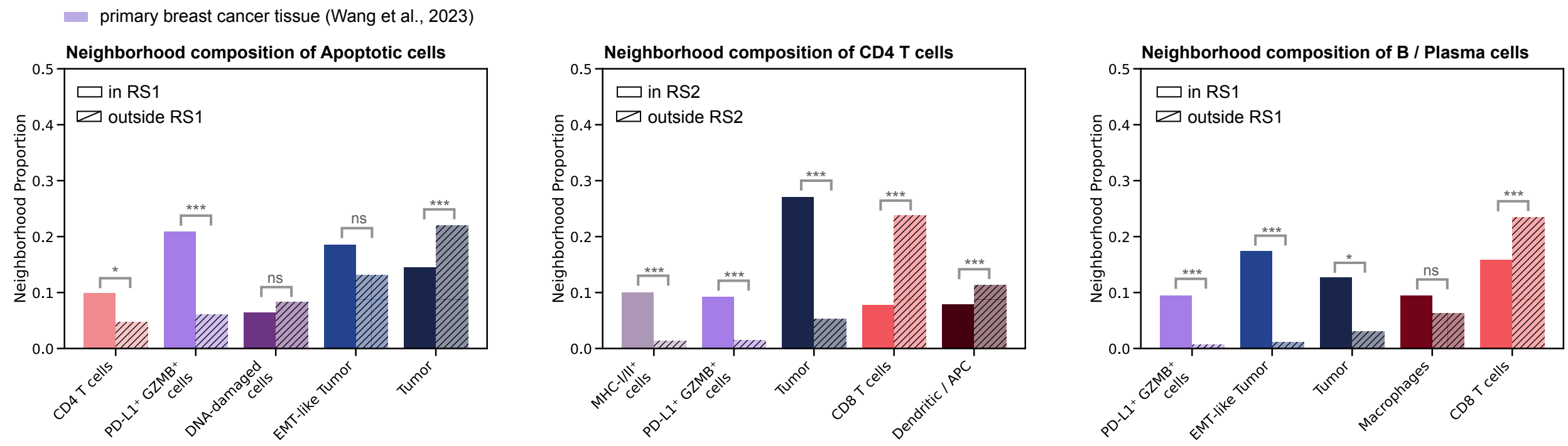

Supplementary Fig. 23: Comparison of neighborhood densities of selected cell types inside and outside the response signatures (RS). Bar diagrams show, from left to right, neighborhood composition of apoptotic cells inside versus outside RS1, CD4 T cells inside versus outside RS2, and B / Plasma cells inside versus outside RS1. Only the five most frequent neighboring cell types are shown. Statistical significance of the differences between inside and outside the signature tested by a two-sided proportions z-test is indicated above brackets: $p < 0.05$ (*), $p < 0.01$ (**), $p < 0.001$ (***); ns indicates $p \geq 0.05$.

Supplementary Tables

Supplementary Tab. 1: Overview of IMC datasets. Only images used for the training and evaluation of VirTues are considered. Cell counts are indicated only for datasets with collected single cell annotations and masks.

Dataset	Tissue Origin	Patients	Images	Crops	Cells
Cords et al.[42]	primary lung cancer	1'048	1'969	50'236	5'880'664
Danenberg et al.[6]	primary breast cancer	688	688	11'837	938'561
Hoch et al.[48]	primary and metastatic melanoma cancer	35	50	2'015	325'881
Jackson et al.[43]	primary breast cancer	284	711	15'788	1'237'470
Damond et al.[81]	healthy and diabetic pancreas	12	843	10'013	1'775'386
Zhu et al.[77]	primary lung cancer	62	1'645	66'982	-
Schulz et al.[53]	primary lung, breast, colon, kidney, head and neck cancer	179	179	3'544	-
Rigamonti et al.[8]	primary lung cancer	83	158	12'793	659'561
Cords et al.[52]	primary breast cancer	12	108	6'281	-
Hu et al.[78]	primary lung cancer, tonsil and endometrium tissue	12	51	4'112	-
Moldoveanu et al.[56]	primary and metastatic melanoma cancer	90	90	4'584	227'592
Schulz et al.[80]	primary breast cancer	77	77	1'758	-
Allam et al.[79]	primary lung cancer	26	26	2'073	-
Wang et al.[2]	primary breast cancer	279	1'842	41'480	2'538'185
Meyer et al.[3]	primary breast cancer	215	450	13'459	928'993
Total	-	3'102	8'887	246'955	≥14'512'293

Supplementary Tab. 2: Overview of additional datasets used for extended multi-technology ablation. Only images used for the training and evaluation of VirTues are considered. Cell counts are indicated only for datasets with collected single cell annotations and masks.

Dataset	Tissue Origin	Patients	Images	Crops	Cells
Phillips et al. [44]	cutaneous lymphoma tissue	14	70	1'766	117'170
Lin et al. [4]	primary colon cancer	40	41	579'287	62'480'753
Lin et al. [85]	primary colon cancer	6	13	30'757	-
Ruf et al. [84]	primary liver cancer	17	17	61'631	-
Monkman et al. [87]	primary lung cancer	42	42	2'754	-
Schürch et al. [83]	primary colon cancer	102	251	5'830	316'570
Consortium [86]	spleen and lymph node tissue	15	80	67'184	-
Cho et al. [93]	primary and metastatic pancreas cancer	12	61	3'963	-
Ehret [94]	colorectal cancer organoid	55	108	5'883	-
Fischer et al. [91]	primary and metastatic breast cancer	892	1'252	34'277	-
Gupta et al. [96]	healthy breast tissue	527	1'492	182'134	3'293'927
Hickey et al. [82]	small intestine and colon tissue	8	64	32'811	-
Malchiodi et al. [92]	primary pancreas cancer	63	164	9'549	-
Rumberger et al. [89]	primary breast cancer	117	1'258	122'786	-
Salié et al. [95]	primary liver cancer	96	194	20'971	-
Xiao et al. [90]	primary melanoma	26	158	6'226	662'266
Liu et al. [88]	healthy tonsil, lymph node, thymus, spleen and placental tissue; primary salivary gland, bladder, breast, soft-tissue and colorectal cancer	28	164	3'042	340'183
Total	-	2'060	5'429	1'170'851	$\geq$ 67'210'869

Supplementary Tab. 3: Nested subsets of markers from Cords et al. [42], as used in the analysis of Fig. 1e, grouped according to their presumed informativeness for general tissue characterization.

Top 10 Markers	Top 11–20 Markers	Top 21–30 Markers	Top 31–40 Markers
FAP	ACTA2	MPO	TCF7
CD68	HLA-DRA	S100A4	MME
CD3E	CA9	H3C1	CD45RA
PDCD1	MS4A1	MCAM	CD248
NT5E	IDO1	CDH11	LYVE1
VIM	MMP9	COL1A1	VWF
FOXP3	PDGFRB	VCAM1	CXCL12
CD8A	CD34	PDPN	CCL21
KRT14	CD4	MMP11	CDH6
MKI67	FUT4	NGFR	CAV1

Supplementary Tab. 4: Grouping of the original cell phenotypes available in the metadata of Danenberg et al.[6] into seven high-level classes used in the cell classification task.

**High-Level Class**	**Original Cell Phenotypes**
Stromal Cell	Fibroblasts<br>Myofibroblasts<br>Endothelial<br>Myofibroblasts $PDPN^+$<br>Fibroblasts $FSP1^+$
Myeloid	$CD15^+$<br>Macrophages<br>Granulocytes<br>$MHC^{high}CD15^+$
Natural Killer Cell	MHC $I^{high}CD57^+$<br>$CD57^+$
ER-Positive Epithelial Cell	CK^+ $CXCL12^+$<br>$ER^{high}CXCL12^+$<br>$HER2^+$<br>Ep $Ki67^+$<br>CK8-18^+ ER^{high}<br>$CK^{low}ER^{med}$
ER-Negative Epithelial Cell	$CK^{med}ER^{low}$<br>$CK^{low}ER^{low}$<br>CK8-$18^{high}CXCL12^{high}$<br>Basal cells<br>CK8-$18^{high}ER^{low}$<br>Ep $CD57^+$
T Cell	$CD8^+$ T cell<br>$CD4^+$ T cell<br>T_{Reg} and T_{Ex} cells
B Cell	B cells<br>$CD38^+$ lymphocytes
Antigen-Presenting Cell	MHC I^{high} & II^{high}

Supplementary Tab. 5: Grouping of the original cell phenotypes from the Hoch et al.[48] metadata into five high-level classes used in the cell classification task.

**High-Level Class**	**Original Cell Phenotypes**
Tumor	Tumor, HLA-DR
T Cell	$CD8^+$ T cells, $CD8^-$ T cells
Macrophage	Macrophage
Lymphocyte	CD38, Neutrophil
Stroma	Stroma, Vasculature
Other	Other

Supplementary Tab. 6: Grouping of the original cell phenotypes available in the metadata of Wang et al. [2] into six high-level classes and 19 low-level classes used in the cell classification task.

High-Level Class	Low-Level Class	Original Cell Phenotypes
Stroma	Fibroblasts	Fibroblasts<br>$PDPN^+$Stromal<br>Myofibroblasts
Immune	Dendritic / APC	DCs<br>PD-$L1^+$APCs<br>PD-$L1^+IDO^+$APCs
	Macrophages	M2 Mac
	B / Plasma cells	$CD79a^+$Plasma<br>$CD20^+$B
	NK cells	$CD56^+$NK<br>$CD56^+$NE
	Neutrophils	$CD15^+$<br>Neutrophils
Tumor	Hypoxic Tumor	$CA9^+$<br>$CA9^+$Hypoxia
	Tumor cells	$CK8/18^{med}$<br>$panCK^{med}$<br>$CK^{hi}GATA3^+$<br>$CK^{lo}GATA3^+$<br>Basal<br>AR^+LAR
	DNA-damage cells	$pH2AX^+$DSB
	EMT-like Tumor	$Vimentin^+$EMT
	Apoptotic cells	Apoptosis
T Cell	CD4 T cells	$CD4^+TCF1^+T$<br>$CD4^+PD1^+T$
	Regulatory T cells	Treg<br>$Helios^+$
	CD8 T cells	$CD8^+T$<br>$CD8^+TCF1^+T$<br>$CD8^+PD1^+T_{Ex}$<br>$CD8^+GZMB^+T$
	Stem-like T cells	$TCF1^+$
Vessel	Endothelial	Endothelial
Other	PD-$L1^+GZMB^+$ cells	PD-$L1^+GZMB^+$
	PD-$L1^+IDO^+$ cells	PD-$L1^+IDO^+$
	MHC-$I\&II^+$ cells	MHC $I\&II^{hi}$

Supplementary Tab. 7: Grouping of the original cell phenotypes available in the metadata of Phillips et al. [44] into eleven high-level classes used in the cell label transfer experiment.

High-Level Class	Original Cell Phenotypes
Tumor	Tumor Cells<br>Tumor Cells Intraepithelial
T Cell	CD4+ T Cells<br>CD8+ T Cells<br>Tregs<br>CLA+ leukocytes
B Cell	B Cells<br>Plasma Cells
Myeloid	DCs CD11c+<br>Macrophages (M1=M2)<br>Macrophages (M1>M2)<br>Macrophages (M2>M1)
Stromal	IDO+ Stromal Cells<br>Stroma
Epithelium	Epithelium
Langerhans	Langerhans Cells
Vessel	Lymphatics<br>Vasculature
Mast	Mast Cells
Nerve	Nerves
Neutrophil	Neutrophils

Supplementary Tab. 8: Grouping of original annotations into harmonized cell types across datasets.

**Harmonized Cell Type**	**Original Cell Types by Dataset**<br>Cords et al. [52]	Danenberg et al. [6]	Wang et al. [2]	Meyer et al. [3]	Hoch et al. [48]	Moldoveanu et al. [56]	Cords et al. [42]	Rigamonti et al. [8]	Schulz et al. [53]	Lin et al. [4]
Tumor	hypoxia<br>normal<br>hypoxic	$CK^{med}ER^{lo}$<br>$ER^{hi}CXCL12^{+}$<br>$CK8\text{-}18^{hi}CXCL12^{hi}$<br>$CK^{lo}ER^{lo}$<br>$CK^{+}$ $CXCL12^{+}$<br>$CK8\text{-}18^{hi}ER^{lo}$<br>$CK8\text{-}18^{+}$ ER^{hi}<br>Ep $Ki67^{+}$<br>$CK^{lo}ER^{med}$<br>$Ki67^{+}$<br>$HER2^{+}$<br>Basal<br>Ep $CD57^{+}$	$CA9^{+}$<br>$CK8/18^{med}$<br>$panCK^{med}$<br>$pH2AX^{+}DSB$<br>$CK^{hi}GATA3^{+}$<br>$CK^{lo}GATA3^{+}$<br>Basal<br>Apoptosis<br>$PD\text{-}L1^{+}IDO^{+}$<br>$AR^{+}LAR$<br>$CA9^{+}$Hypoxia	tumor	Tumor	melano	Tumor cells<br>Ki67+	normal<br>hypoxic	Tumor	Tumor<br>PD-L1+ tumor
Fibroblast / Stroma	Vimentin high<br>SMA-Fibro<br>CD10-CAF<br>FN-Cdh11-mCAF<br>Fibroblast<br>vCAF<br>CXCL13-CAF<br>CD73-CAF<br>CCL21-CAF<br>IDO-CAF<br>CD34-CAF<br>CA9-CD10-CAF	Fibroblasts<br>Myofibroblasts $PDPN^{+}$<br>Myofibroblasts<br>Fibroblasts $FSP1^{+}$	Fibroblasts<br>$PDPN^{+}$Stromal<br>Myofibroblasts<br>$Vimentin^{+}$EMT	Stromal	Stroma		aSMA+<br>Vim	Collagen-CAF<br>hypoxic-CAF<br>mCAF<br>SMA-CAF<br>tpCAF<br>iCAF<br>vCAF<br>dCAF<br>hypoxic-tpCAF<br>IDO-CAF<br>PDPN-CAF	Mural	Stroma<br>Vascular smooth muscle
Myeloid	Myeloid<br>Macrophage<br>Neutrophil	Macrophages<br>$CD15^{+}$<br>Macrophages & granulocytes<br>Granulocytes<br>$MHC^{hi}CD15^{+}$	DCs<br>M2 Mac<br>$PD\text{-}L1^{+}$APCs<br>$CD15^{+}$<br>Neutrophils<br>$PD\text{-}L1^{+}IDO^{+}$APCs	HLA-DR<br>Monocyte<br>Neutrophil	Macrophage<br>Neutrophil<br>HLA-DR	macro.mono	Mf	Myeloid<br>Neutrophil	MacCD163<br>DC<br>HLADR<br>Neutrophil<br>pDC	M2-like<br>Macrophage
CD8 T cell	CD8-Treg<br>CD8<br>ki67-CD8<br>CD8-CXCL13<br>IDO-CD8	$CD8^{+}$ T cells	$CD8^{+}$T<br>$CD8^{+}TCF1^{+}$T<br>$CD8^{+}PD1^{+}$T-Ex<br>$CD8^{+}GZMB^{+}$T<br>$TCF1^{+}$	CD8-T-cell	CD8+ T cell	Tc.ae<br>Tc.naive	CD8+	ki67-CD8<br>CD8<br>IDO-CD8<br>TCF1/7-CD8	CD8	CTL<br>Memory T<br>Exhausted CD8 T cell
CD4 T cell	CD4-Treg<br>CD4<br>ki67-CD4<br>CD4-CXCL13<br>IDO-CD4	$CD4^{+}$ T cells & APCs<br>$CD4^{+}$ T cells	$CD4^{+}TCF1^{+}$T<br>Treg<br>$CD4^{+}PD1^{+}$T<br>$Helios^{+}$	CD4-T-cell	CD8- T cell	Th.ae<br>Treg<br>Th.naive	CD4+<br>Treg	CD4<br>ki67-CD4<br>IDO-CD4<br>TCF1/7-CD4<br>CD4-Treg<br>PD1-CD4	CD4<br>Treg	Th<br>Treg
B cell	Bcell	$CD38^{+}$ lymphocytes<br>B cells	$CD79a^{+}$Plasma<br>$CD20^{+}$B	B-cell	CD38	B	Bcells	Bcell	plasma<br>B	B cell
Vessel / Endo.	Blood<br>Lymphatic<br>HEV	Endothelial	Endothelial	Endothelial	Vasculature	CD31	vessels	HEV<br>Blood<br>Lymphatic		Endothelial
Unknown	other<br>T cell	MHC I & II^{hi}<br>T-Reg & T-Ex<br>$CD57^{+}$<br>MHC $I^{hi}CD57^{+}$	$CD56^{+}$NK<br>$PD\text{-}L1^{+}GZMB^{+}$<br>MHC $I\&II^{hi}$<br>$CD56^{+}$NE	Immune-low	Other	others	other	Other<br>other	unlabelled<br>NK<br>BnT	T Cell<br>Other

Supplementary Tab. 9: Pairings of evaluation and training datasets for cross-cohort cell typing with MAPS [54]. For each evaluation dataset, the corresponding training dataset was selected to maximize the number of shared markers while matching the organ of origin whenever possible.

Evaluation Dataset	Training Dataset
Cords et al. [52]	Wang et al. [2]
Danenberg et al. [6]	Wang et al. [2]
Meyer et al. [3]	Wang et al. [2]
Wang et al. [2]	Danenberg et al. [6]
Cords et al. [42]	Rigamonti et al. [8]
Rigamonti et al. [8]	Cords et al. [42]
Moldoveanu et al. [56]	Hoch et al. [48]
Schulz et al. [53]	Wang et al. [2]
Lin et al. [4]	Wang et al. [2]

Supplementary Tab. 10: Canonical markers per dataset and harmonized cell type used for configuration of Astir.

**Harmonized Cell Type**	**Original Cell Types by Dataset**								
	Cords et al. [52]	Danenberg et al. [6]	Wang et al. [2]	Meyer et al. [3]	Moldoveanu et al. [56]	Cords et al. [42]	Rigamonti et al. [8]	Schulz et al. [53]	Lin et al. [4]
Tumor	Keratin-AE1-AE3	panCK	panKeratin-AE3	pan Cytokeratin	SOX10<br>S100	PanCytokeratin	panCK	Ecad	Pan-CK<br>E-cadherin
Fibroblast / Stroma	FAP<br>SMA<br>Vimentin<br>Fibronectin-Collagen-I<br>Cadherin-11	SMA<br>PDGFRB	SMA<br>Vimentin<br>PDGFRbeta	SMA<br>Vimentin<br>Fibronectin		aSMA<br>Vimentin<br>Coll-I	SMA<br>FAP<br>CDH11<br>Collagen I<br>VIM<br>PDGFRB	SMA<br>PDGFRb	SMA
Myeloid	CD68<br>CD11c<br>HLA-DR	CD68<br>CD15<br>CD11c<br>HLA-DR	CD68<br>CD11c<br>CD15<br>HLA-DR	CD15<br>CD68<br>CD11c<br>HLA-DR	CD68<br>CD14<br>HLA-DR	CD68<br>CD14<br>HLA-DR	CD68<br>CD15	CD68<br>CD15<br>CD11c<br>HLA-DR	CD68<br>CD163
CD8 T cell	CD3<br>CD8a	CD3e<br>CD8	CD3<br>CD8	CD3<br>CD8a	CD3<br>CD8a	CD3<br>CD8	CD3e<br>CD8	CD3<br>CD8a	CD3e<br>CD8a
CD4 T cell	CD3<br>CD4	CD3e<br>CD4	CD3<br>CD4	CD3<br>CD4	CD3<br>CD4	CD3<br>CD4	CD3e<br>CD4	CD3<br>CD4	CD3e<br>CD4
B cell	CD20	CD20	CD20	CD20	CD20	CD20	CD20	CD20	CD20
Vessel / Endo.	vWF-CD31<br>CD31<br>Podoplanin	CD31-vWF<br>PDPN	CD31<br>Podoplanin	CD31	CD31	CD31	vWF<br>LYVE-1<br>PDPN		CD31

Supplementary Tab. 11: List of datasets and source repositories.

Dataset	Data Source URL
Cords et al.[42]	https://doi.org/10.5281/zenodo.7961843
Danenberg et al.[6]	https://doi.org/10.5281/zenodo.5850951
Jackson et al.[43]	https://doi.org/10.5281/zenodo.3518283
Hoch et al.[48]	https://doi.org/10.5281/zenodo.6004985
Damond et al.[81]	https://doi.org/10.17632/cydmwsfztj.2
Zhu et al.[77]	https://www.synapse.org/Synapse:syn54951674/wiki/626860 (ID: syn61802885)
Schulz et al.[53]	https://doi.org/10.5281/zenodo.12912566
Rigamonti et al.[8]	Received from authors via data access request
Cords et al.[52]	https://doi.org/10.5281/zenodo.5769017
Hu et al.[78]	https://doi.org/10.5281/zenodo.6784250
Moldoveanu et al.[56]	https://doi.org/10.5281/zenodo.5903189
Schulz et al.[80]	https://doi.org/10.17632/m4b97v7myb.1
Allam et al.[79]	https://doi.org/10.5281/zenodo.6784252
Wang et al.[2]	https://doi.org/10.5281/zenodo.7990869 (data access request required)
Meyer et al.[3]	https://doi.org/10.5281/zenodo.10890543
Phillips et al.[44]	https://immunoatlas.org/NOLN/210920-1/
Lin et al.[4]	https://doi.org/10.5281/zenodo.7637655
Consortium[86]	https://portal.hubmapconsortium.org/
Cho et al.[93]	https://doi.org/10.5281/zenodo.15601930
Ehret[94]	https://doi.org/10.5281/zenodo.15198803
Fischer et al.[91]	https://doi.org/10.5281/zenodo.7494726
Gupta et al.[96]	https://doi.org/10.5281/zenodo.18418220
Hickey et al.[82]	https://doi.org/10.5061/dryad.gmsbcc2sq
Lin et al.[85]	https://doi.org/10.5281/zenodo.7506942
Liu et al.[88]	https://doi.org/10.5281/zenodo.5542726
Malchiodi et al.[92]	https://doi.org/10.5281/zenodo.10582037
Monkman et al.[87]	https://doi.org/10.5281/zenodo.10258577
Ruf et al.[84]	https://doi.org/10.7937/BHOR-Y074
Rumberger et al.[89]	https://huggingface.co/datasets/JLrumberger/Pan-Multiplex/tree/main
Salié et al.[95]	https://doi.org/10.5281/zenodo.10793608<br>https://doi.org/10.5281/zenodo.10794903
Schürch et al.[83]	https://doi.org/10.7937/TCIA.2020.FQN0-0326
Xiao et al.[90]	https://doi.org/10.5281/zenodo.6836991

Supplementary Discussion

Further limitations

Most of VirTues' current limits trace to a single source: the model is only as broad as the data it has seen. Because markers are represented through their protein sequences, reconstruction and prediction stay strong for markers resembling those in the training set but degrade as biochemical relatedness weakens. Virtual augmentation is therefore best treated as a hypothesis to be confirmed against measured channels rather than a substitute for direct measurement. Limited coverage likewise makes rare cell states and uncommon tissue architectures difficult, reflecting the composition of the corpus rather than any architectural ceiling. And while our data span 32 cohorts across 17 organ sites and four technologies, establishing universality will require broader validation across diseases, tissue-processing protocols, and imaging platforms. In each of these cases the remedy is the same, targeted curation of underrepresented contexts, and we expect performance to keep improving as the corpus grows.

A second set of caveats concerns how far the present results can be pushed. Our survival analyses, while supportive, are primarily unadjusted demonstrations of signal; covariate-adjusted models, assessment of proportional hazards across independent cohorts, and prospective validation are required for clinical adoption. Interpretation carries a similar qualification: attention maps localize the markers and regions a prediction draws on, but they capture only part of the model's behavior, and causal or perturbational analyses will be needed to confirm that these features are mechanistically, rather than merely correlatively, informative.

A final limitation is intrinsic to the assay rather than the model. Spatial proteomics yields deep molecular readouts but is costly and tissue-intensive, whereas H&E is cheap, fast, and ubiquitous[21,69]. Fusing the two is a natural next step, both to extend VirTues toward routinely available data and to pair molecular depth with scalable morphology in a single model.
