# Supplementary material for: The Virtual Tissues foundation model resolves spatial proteomics across scales
Source: Nature. 2026 Aug 5;657(8132):744–54. doi: 10.1038/s41586-026-10884-y (PMC13581600; doi:10.1038/s41586-026-10884-y)
Supplement: Supplementary file 2 [file 41586_2026_10884_MOESM2_ESM.pdf]

nature portfolio

Corresponding author(s): Charlotte Bunne

Last updated by author(s): Jun 24, 2026

Reporting Summary

Nature Portfolio wishes to improve the reproducibility of the work that we publish. This form provides structure for consistency and transparency in reporting. For further information on Nature Portfolio policies, see our Editorial Policies and the Editorial Policy Checklist.

Statistics

For all statistical analyses, confirm that the following items are present in the figure legend, table legend, main text, or Methods section.

n/a	Confirmed	
☐	☒	The exact sample size (*n*) for each experimental group/condition, given as a discrete number and unit of measurement
☒	☐	A statement on whether measurements were taken from distinct samples or whether the same sample was measured repeatedly
☐	☒	The statistical test(s) used AND whether they are one- or two-sided<br>*Only common tests should be described solely by name; describe more complex techniques in the Methods section.*
☒	☐	A description of all covariates tested
☒	☐	A description of any assumptions or corrections, such as tests of normality and adjustment for multiple comparisons
☐	☒	A full description of the statistical parameters including central tendency (e.g. means) or other basic estimates (e.g. regression coefficient) AND variation (e.g. standard deviation) or associated estimates of uncertainty (e.g. confidence intervals)
☐	☒	For null hypothesis testing, the test statistic (e.g. *F*, *t*, *r*) with confidence intervals, effect sizes, degrees of freedom and *P* value noted<br>*Give P values as exact values whenever suitable.*
☒	☐	For Bayesian analysis, information on the choice of priors and Markov chain Monte Carlo settings
☒	☐	For hierarchical and complex designs, identification of the appropriate level for tests and full reporting of outcomes
☐	☒	Estimates of effect sizes (e.g. Cohen's *d*, Pearson's *r*), indicating how they were calculated

Our web collection on statistics for biologists contains articles on many of the points above.

Software and code

Policy information about availability of computer code

Data collection	All data were obtained from publicly available sources or through correspondence and data access requests. Data preprocessing was performed using Python (v3.12.9) with the following libraries: NumPy (v2.2.4), scikit-image (v0.25.2), zarr (v3.1.5), and pandas (v2.2.3).
Data analysis	The following software was used for data analysis: Python (v3.12.9), NumPy (v2.2.4), PyTorch (v2.5.1, CUDA 12.1), FlashAttention-2 (v2.7.4), fair-esm (v2.0.0), pandas (v2.2.3), matplotlib (v3.10.3), seaborn (v0.13.2), scikit-learn (v1.5.2), scikit-image (v0.25.2), scikit-survival (v0.24.1), lifelines (v0.30.0), cuML (v25.8.0), instanseg-torch (v0.1.1), napari (v0.5.5), wsireg (v0.3.10) and QuPath (v0.5.1).<br><br>Code developed for this study can be accessed at: https://github.com/bunnelab/virtues

For manuscripts utilizing custom algorithms or software that are central to the research but not yet described in published literature, software must be made available to editors and reviewers. We strongly encourage code deposition in a community repository (e.g. GitHub). See the Nature Portfolio guidelines for submitting code & software for further information.

Data

Policy information about availability of data

All manuscripts must include a data availability statement. This statement should provide the following information, where applicable:

- Accession codes, unique identifiers, or web links for publicly available datasets
- A description of any restrictions on data availability
- For clinical datasets or third party data, please ensure that the statement adheres to our policy

All datasets used in this study were collected from public sources, except those from Rigamonti et al. (2024) and Wang et al. (2024), which were obtained from the respective authors through data access requests. The complete harmonized data corpus, including derived annotations across multiple spatial proteomics technologies, is available at https://github.com/bunnelab/virtues#datasets with the exception of the dataset of Wang et al. (2024) (https://doi.org/10.5281/zenodo.7990869) which cannot be redistributed due to licensing restrictions.

The publicly available datasets including their source repository are as follows: Cords et al. (2024) (https://doi.org/10.5281/zenodo.7961843), Danenberg et al. (2022) (https://doi.org/10.5281/zenodo.5850951), Jackson et al. (2020) (https://doi.org/10.5281/zenodo.3518283), Hoch et al. (2022) (https://doi.org/10.5281/zenodo.6004985), Damond et al. (2019) (https://doi.org/10.17632/cydmwsfztj.2), Zhu et al. (2025) (https://www.synapse.org/Synapse:syn54951674/wiki/626860; syn61802885), Schulz et al. (2024) (https://doi.org/10.5281/zenodo.12912566), Cords et al. (2023) (https://doi.org/10.5281/zenodo.5769017), Hu et al. (2023) (https://doi.org/10.5281/zenodo.6784250), Moldoveanu et al. (2022) (https://doi.org/10.5281/zenodo.5903189), Schulz et al. (2018) (https://doi.org/10.17632/m4b97v7myb.1), Allam et al. (2022) (https://doi.org/10.5281/zenodo.6784252), Meyer et al. (2025) (https://doi.org/10.5281/zenodo.10890543), Phillips et al. (2021) (https://immunoatlas.org/NOLN/210920-1/), Lin et al. (2023) (https://doi.org/10.5281/zenodo.7637655), HuBMAP-Consortium (2019) (https://portal.hubmapconsortium.org/), Cho et al. (2025) (https://doi.org/10.5281/zenodo.15601930), Ehret (2025) (https://doi.org/10.5281/zenodo.15198803), Fischer et al. (2023) (https://doi.org/10.5281/zenodo.7494726), Gupta et al. (2026) (https://doi.org/10.5281/zenodo.18418220), Hickey et al. (2023) (https://doi.org/10.5061/dryad.gmsbcc2sq), Lin et al. (2022) (https://doi.org/10.5281/zenodo.7506942), Liu et al. (2022) (https://doi.org/10.5281/zenodo.5542726), Malchiodi et al. (2024) (https://doi.org/10.5281/zenodo.10582037), Monkman et al. (2024) (https://doi.org/10.5281/zenodo.10258577), Ruf et al. (2023) (https://doi.org/10.7937/BH0R-Y074), Rumberger et al. (2025) (https://huggingface.co/datasets/JLrumberger/Pan-Multiplex/tree/main), Salié et al. (2025) (https://doi.org/10.5281/zenodo.10793608; https://doi.org/10.5281/zenodo.10794903), Schürch et al. (2020) (https://doi.org/10.7937/TCIA.2020.FQN0-0326), Xiao et al. (2022) (https://doi.org/10.5281/zenodo.6836991).

Protein marker sequences were obtained from https://www.uniprot.org/ and the specific UniProt IDs used can be found in the harmonized data corpus. A tool to automatically download the corresponding amino acid sequences is provided with the code.

Trained model weights are available at https://github.com/bunnelab/virtues#models.

Research involving human participants, their data, or biological material

Policy information about studies with human participants or human data. See also policy information about sex, gender (identity/presentation), and sexual orientation and race, ethnicity and racism.

Reporting on sex and gender	n/a
Reporting on race, ethnicity, or other socially relevant groupings	n/a
Population characteristics	n/a
Recruitment	n/a
Ethics oversight	n/a

Note that full information on the approval of the study protocol must also be provided in the manuscript.

Field-specific reporting

Please select the one below that is the best fit for your research. If you are not sure, read the appropriate sections before making your selection.

☒ Life sciences ☐ Behavioural & social sciences ☐ Ecological, evolutionary & environmental sciences

For a reference copy of the document with all sections, see nature.com/documents/nr-reporting-summary-flat.pdf

Life sciences study design

All studies must disclose on these points even when the disclosure is negative.

Sample size	Sample sizes were predetermined by the datasets used in the study. With the exception of the data filtering described below, all available samples are used. P-values are indicated throughout the study to validate the statistical significance of conclusions.

Data exclusions | During quality control, we filtered out IMC images with insufficient tissue coverage due to tearing or damage to the TMA cores.

Replication | Attempts at replication were successful for the reported model results. The model implementation and weights can be accessed at https://github.com/bunnelab/virtues.

Randomization | Each dataset was divided randomly into an 80/20 train–test split, ensuring that all samples from a given patient were contained entirely within either the training or the testing set. For the biomarker analysis on the dataset of Wang et al. (2023), we used repeated cross validation following the procedure of Wang et al. (2023) to ensure comparability of the results (see Methods for further details).

Blinding | Test splits were withheld from both pretraining and downstream training, but no blinding was performed. Fully blinded tests would have been impractical due to the uneven split sizes, which were necessary to ensure sufficient training data given the moderate sample sizes of the available datasets.

Reporting for specific materials, systems and methods

We require information from authors about some types of materials, experimental systems and methods used in many studies. Here, indicate whether each material, system or method listed is relevant to your study. If you are not sure if a list item applies to your research, read the appropriate section before selecting a response.

Materials & experimental systems

n/a	Involved in the study
☒	☐ Antibodies
☒	☐ Eukaryotic cell lines
☒	☐ Palaeontology and archaeology
☒	☐ Animals and other organisms
☒	☐ Clinical data
☒	☐ Dual use research of concern
☒	☐ Plants

Methods

n/a	Involved in the study
☒	☐ ChIP-seq
☒	☐ Flow cytometry
☒	☐ MRI-based neuroimaging

Plants

Seed stocks | *Report on the source of all seed stocks or other plant material used. If applicable, state the seed stock centre and catalogue number. If plant specimens were collected from the field, describe the collection location, date and sampling procedures.*

Novel plant genotypes | *Describe the methods by which all novel plant genotypes were produced. This includes those generated by transgenic approaches, gene editing, chemical/radiation-based mutagenesis and hybridization. For transgenic lines, describe the transformation method, the number of independent lines analyzed and the generation upon which experiments were performed. For gene-edited lines, describe the editor used, the endogenous sequence targeted for editing, the targeting guide RNA sequence (if applicable) and how the editor was applied.*

Authentication | *Describe any authentication procedures for each seed stock used or novel genotype generated. Describe any experiments used to assess the effect of a mutation and, where applicable, how potential secondary effects (e.g. second site T-DNA insertions, mosiacism, off-target gene editing) were examined.*
