# Supplementary material for: The Virtual Tissues foundation model resolves spatial proteomics across scales
Source: Nature. 2026 Aug 5;657(8132):744–54. doi: 10.1038/s41586-026-10884-y (PMC13581600; doi:10.1038/s41586-026-10884-y)
Supplement: Supplementary file 3 [file 41586_2026_10884_MOESM3_ESM.pdf]

nature portfolio

Peer Review File

The Virtual Tissues foundation model resolves spatial proteomics across scales

Corresponding Author: Professor Charlotte Bunne

Any redactions in this file are there to maintain patient confidentiality, the confidentiality of unpublished data, or to remove third-party material.

This file contains all reviewer reports in order by version, followed by all author rebuttals in order by version.

Version 0:

Reviewer comments:

Referee #1

(Remarks to the Author)
This manuscript presents VirTues, a spatial proteomic foundation model that learns rich representations from imaging mass cytometry (IMC) patches. The model shows improved performance in marker reconstruction, cell phenotyping, and niche annotation tasks. In addition, VirTues-derived features identified spatial grounding patterns associated with treatment response and disease-free survival in triple-negative breast cancer (TNBC). Below are our comments.

General comments:
Overall, this manuscript presents an ambitious foundation-model approach for spatial proteomics and demonstrates promising performance across multiple downstream tasks using IMC data. Several methodological and conceptual limitations currently reduce the strength of the conclusions. Key concerns include limited evidence of generalizability beyond IMC platforms, incomplete descriptions in marker selection and reporting, and marginal performance improvement in clinically critical settings such as pretreatment pCR prediction. Clarification of modeling choices, evaluation protocols, and biological assumptions could further strengthen rigor, interpretability, and translational relevance.

Specific comments:
1. This manuscript focuses exclusively on IMC imaging techniques in developing the VirTues model. However, the generalizability of VirTues to other widely used multiplexed immunofluorescence (mIF) approaches, such as Cycif, Codex, and MIBI, remains unknown. These techniques often provide higher spatial resolution and more detailed characterization of the tumor microenvironment.
2. The datasets used in this study include more than 40 markers. Several of them, such as ER, PR, HER2, PD-L1, GZMB, play critical roles in tumor-immune interactions. The authors could more explicitly justify their strategy for selecting these 40 markers and address potential selection bias to support the robustness of their findings.
3. Similar concerns apply to the zero-shot marker reconstruction experiments. Not all evaluated markers are reported in the main text or supplemental materials.
4. The performance of cell phenotype prediction appears limited when applied to previously unseen biomarkers, particularly for rare cell populations that may play essential roles in the TME. This limitation may restrict the model’s utility for discovering novel niches or rare but biologically important cell types.
5. Zero-shot marker reconstruction performance is sometimes limited and can underperform simple baselines, such as the average or the most highly correlated marker, especially for new markers. This limits the model’s generalization to cohorts with different biomarker panels and suggests the possibility of shortcut learning. A detailed error analysis would strengthen this section.
6. For pathological complete response (pCR) prediction in Fig. 4f, the manuscript reports results from five runs using different random seeds. Five-fold cross-validation would be preferable to improve statistical power and reliability. In addition, VirTues underperforms CA-MAE for pretreatment TNBC pCR prediction, which is arguably the most clinically relevant time point for informing early treatment decisions.
7. Regarding the identification of foundation-model-based biomarkers, the authors could clarify the rationale for the chosen

number of repetitions (e.g., 10 repetitions for cluster rank selection and 25 for joint prediction). Do these experiments share the same data split across folds? In addition, the comparison across biomarkers is limited. The authors could strengthen this analysis by expanding the reference biomarker set and including an additional baseline that uses tabular spatial single-cell data to replicate the same workflow.
8. Ground truth inaccuracy: The authors should discuss the potential sources of error or uncertainty in the cell-type annotations, as this may affect the validity of performance evaluations. Providing refined or updated cell phenotype labels would be a valuable contribution to the research community.
9. On page 18, the terminology of "marker attention" and "channel attention" is a bit ambiguous. In the VirTues encoder, the index m appears to be used interchangeably to denote both the m-th channel and the m-th marker. Clearer definitions and notation would improve interpretability.
10. On page 18, the decoder input is described as concatenating non-masked encoded tokens with masked image tokens (i.e., the tokens prior to masking). However, in real zero-shot scenarios, these image tokens are not available. The authors could clarify how masked tokens are decoded in practice.
11. For heteromeric proteins, did the authors focus exclusively on the primary signaling chain (e.g., the alpha-chain) while excluding other chains? If so, how might this simplification affect the learned representations? Would the results differ if alternative chains were used (e.g., CD8A versus CD8B)?
12. Could the authors provide the exact amino acid sequence used as inputs to the ESM-2 model for each biomarker to improve reproducibility?
13. In Supplemental Figure S12, using the mean intensity of each marker to separate components may not be optimal. A Gaussian mixture model (GMM) or similar probabilistic approach may yield a more robust separation.
14. In Figure 5b, the distributional shifts across pre-, on-, and post-treatment samples are not visually pronounced. Including a formal statistical test would help clarify whether these differences are meaningful.
15. Existing methods, such as GigaTIME[1] and HistoPlexer[2], generate virtual mIF tissues and offer a cost- and time-efficient approach for deriving proteomic information and characterizing the tumour immune microenvironment. Could the authors clarify how VirTues improves beyond these prior models?

Additional minor points:
1. Page 9 Line 284: There is a typographical error ("the the") in the sentence: First, we compared whether the the retrieved 285 samples matched the reference samples in their clinical and diagnostic variables.

[1]. Valanarasu, Jeya Maria Jose, et al. "Multimodal AI generates virtual population for tumor microenvironment modeling." Cell (2025).
[2]. Andani, Sonali, et al. "Histopathology-based protein multiplex generation using deep learning." Nature Machine Intelligence 7.8 (2025): 1292-1307.

(Remarks on code availability)
The code provides a README file with instructions for running the application.

Referee #2

(Remarks to the Author)
The authors designed and trained a transformer-based MAE, which they call Virtual Tissues or VirTues, with the following changes to the architecture, training, and inference that make it more amenable to processing highly-multiplex imaging data like those produced by immunofluorescent microscopy and imaging mass cytometry:

1. a factorized attention scheme in the encoder that alternates between channel-only and spatial-only blocks, allowing the encoder process many dozens of channels per image,

2. ESM2 embeddings as input tokens, which are added to patch tokens and encode the identity of each channel's imaged protein, allowing flexible processing of the various channel combinations during training and inference, and

3. a channel of "patch summary" input tokens along with a decoding scheme which forwards each encoded channel, together with the encoded patch summary channel, through the decoder individually, encouraging the information needed for reconstruction to be stored in the encoded patch summary tokens and not in the cross-channel attention weights.

The authors use the VirTues embeddings in a wide variety of clinically-relevant tasks in which they outperform related vision models and methods:

Niche and marker inpainting:
- Reconstructions are qualitatively (visually) plausible, even in the zero-shot marker case.
- Reconstructions are quantitatively similar to IID inpainting, except in the difficult zero-shot marker inpainting case, and even this is poor for only 3 of the 8 new markers.

Cell type classification:
- More accurate than KRONOS, and dramatically more accurate than CA-MAE, on classifying cell types from cell summary embeddings in the in-domain, zero-shot, and cross-cohort transfer settings, especially on rare cell types.
- This results in a more accurate cell-level annotation of the entire tissue, as qualitatively demonstrated in Figure 3f

Patient stratification:
- ER-positive patients, represented by the proportion of their cell summary embeddings across 120 “phenotypic states” (k-means clusters), clustered into high-risk and low-risk groups with statistically significant differences in their survival curves with concordant differences in the structures of their tumor microenvironments.

Tissue diagnostic annotations:
- VirTues embeddings generally outperformed KRONOS, CA-MAE and ResNet50 embeddings when used as input to an ABMIL classification model for subtype and grade in lung cancer, and ER status, ERBB2 status, PAM50 subtype, and grade in breast cancer.

Tissue prognostic annotations:
- VirTues embeddings generally outperformed KRONOS, CA-MAE and ResNet50 embeddings when used as input to an ABMIL classification model for treatment response in triple-negative breast cancer.

Tissue database lookup:
- VirTues embeddings generally outperformed KRONOS, CA-MAE and ResNet50 when retrieving similar tissues from a database of patient images

Predictive biomarker discovery for treatment response and disease-free survival:
- VirTues embeddings separate therapy response better than mean marker intensities, with responders showing greater separation than non-responders.
- VirTues embeddings of pre-treatment samples were used to create a “virtual” biomarker of 4 response signatures (2 response, 2 non-response) of distinct heterogeneous cell populations, which were more predictive of response than previous work and common clinical immune-ratio baselines.
- These response signatures were transferred to a OOD triple-negative breast cancer cohort via trained classifiers, leading to a stratification of patients into low-, medium, and high-risk groups that achieve a concordance index higher than competing methods and immune-ratio baselines.

These results represent a significant scientific contribution to the computational understanding of cancer using multiplex imaging of marker proteins.

VirTues is most closely related to KRONOS, yet the changes in architecture, training, and inference described above are novel and represent an original contribution to the field.

The methodologies and statistical tests used throughout this work are sound, and limitations are addressed in the Discussion.

The data used is relevant and represents a standard collection of publicly-available multiplex cancer patient samples. In fact, this work is more comprehensive than other published works in that it is able to combine datasets have vastly different sets of markers.

The presentation, including figures, is generally of very high quality and helpful for understanding the dense collection of results in this paper.

Questions:

1. Zero-shot marker reconstruction: it is reasonable to assume that the performance of the new markers in the zero-shot reconstruction case would be a function of their similarity or functional relationship to markers in the training set. Figure 2f seems to be an attempt at showing this, but the UMAP is far from helpful in this regard. Do you see any evidence for such a function? Whether or not you do, it would be helpful to comment on this in the paper.

2. The many appeals to reconstructions being visually plausible are hard to judge, even for experts. Visually plausible and biologically valid are far from the same thing. Is there a way you could provide evidence for the biological validity of the reconstructions? E.g., do inferred annotations remain the same between the original and inpainted images?

3. Why weren’t KRONOS and CA-MAE embeddings used for comparison in the patient stratification task?

4. So many of these tasks require assigning image patches to cells and tissues, which is done using software other than VirTues. How do these results depend on the choice of assignment? And why not train a cell and tissue assignment model together with VirTues?

5. How are niche summary tokens produced? The text says they are “trained to capture tissue composition, architecture and interactions between different cellular neighborhoods” but I don’t see an explanation of this in the text.

6. Why do you use all tokens when calculating reconstruction metrics, rather than just the masked one? It is far more common to report the latter, and avoids the bias resulting from including “reconstructed” unmasked tokens.

7. The use of OT-based approaches for tissue retrieval is done without comment or explanation. Do the results change if another distributional-distance measure is used? Please explain why this and not other means of measuring token similarity

was chosen.

8. Please comment on why single-cell morphology models like scDINO [1] or Doron et al. [2] were not included with KRONOS and CA-MAE as competitive baselines. Did you try to build representations from such models, and if so, what were the results?

Suggestions:

1. Using "virtual" in the name of this model is possibly problematic as recently the term has come to refer to models that predict the outcomes of interventions on the data. In this paper, VirTues is used only to represent data, and each application either 1) compares / clusters embeddings, or 2) classifies / annotates embeddings, and lacks any in silico simulation of interventions. I recommend the following:
- Add such an application, e.g. something similar to the T-cell effector state simulation reported in this blog post by Noetik [3].
- Address the recent virtual cell literature more directly (e.g. the oft-cited Cell perspective by Bunne et al.) and describe how VirTues fits into it.

2. It is incorrect to refer to the pretrained model as multi-scale. It is trained at a single scale (128 x 128 crop of original image), and only the embeddings are aggregated (sometimes via learning) at various scales to provide representations of cells, niches, tissues, and patients. A true multiscale model would be trained using inputs at multiple scales, not derive them after the fact.

3. I think it can be misleading to call the proposed encoder attention scheme "sparse" - it is factorized into alternating layers of dense channel-only and spatial-only attention blocks. This is not a terribly important point, but I would recommend referring to this as factorized / alternating attention rather than sparse.

4. Unlike KRONOS, CA-MAE was not purposefully designed for applications like spatial proteomics. While it is fair to make comparisons with CA-MAE since it is one of the few vision models that is intended to encode multiplex cellular microscopy images, it is also fair to mention that the intended application of CA-MAE was encoding Cell Painting and Brightfield images.

5. The section titled "Spatial and marker attention provides explainability of algorithmic decisions" is an interesting examination of the connection of markers and learned attention weights, but it is at best unclear what "algorithmic decisions" is referring to, since there are no decisions made, algorithmic or not, either in this section or in the rest of the paper. I recommend clarifying what is meant by this phrase with examples, or rewording the section and title to more accurately reflect the scope of the results.

6. The tree diagrams in Figure 5g are visually harder to interpret than simple bar graphs, I recommend changing these.

[1] Pfaendler, Ramon, et al. "Self-supervised vision transformers accurately decode cellular state heterogeneity." Biorxiv (2023): 2023-01.
[2] Doron, Michael, et al. "Unbiased single-cell morphology with self-supervised vision transformers." bioRxiv (2023).
[3] https://www.noetik.blog/p/how-do-you-use-a-virtual-cell-to-6c6

(Remarks on code availability)
The code is runnable, with useful README and notebooks, however I did not get as far as reproducing the results. The weights of the trained model are also on HF but I did not try to load this model.

Version 1:

Reviewer comments:

Referee #1

(Remarks to the Author)
The authors have addressed the majority of our concerns. The revised manuscript shows substantial improvements, particularly in the standardization of terminology and the enhanced demonstration of generalizability across multiplexed imaging platforms, including CODEX and Orion.

1. Regarding the newly integrated CODEX and Orion datasets, some of these datasets provide tabular single-cell expression matrices rather than curated phenotype labels. The current method, which references general gating and clustering procedures, lacks sufficient detail to ensure reproducibility. In addition, making the curated single-cell phenotype labels publicly available would greatly benefit the spatial proteomics community.

2. Cell phenotyping performance for clinically important biomarkers, such as PD-L1+ and exhausted T cells, remains suboptimal (F1 score < 0.5 in Orion). Given their critical clinical significance and the inherent challenges in establishing reliable ground truth for these cell populations, it would be valuable provide a high-quality subset of representative cases for visualization. This would better demonstrate the practical applicability of VirTues.

3. 95% confidence intervals (95% CIs) are missing from several key figures, including Figure 1e; Figures 3b, 3c, 3d, and 3g;

Figure 4h; Figure 5k; and multiple panels in the Supplemental Figures. For Figure 5e, DeLong's test should be used for ROC comparison. Additionally, please justify the use of a one-sided Mann-Whitney U test in the tissue-level classification Section on Page 24.

4. Some CIs are not described in the corresponding figure legends (e.g., Figure 4f). In addition, please report exact p-values throughout the manuscript, except in cases where $P < 0.001$ (e.g., $P < 0.1$ and $P < 0.07$ on Pages 8 and 9).

5. Figure 1d visually suggests that VirTues operates on approximately 40 markers; however, the text states that VirTues was trained on more than 140 distinct markers. Please clarify this discrepancy.

6. In Figure S19, several bars corresponding to VirTues are missing. Please clarify whether these represent zero values or missing data.

(Remarks on code availability)
The code provides a README file with instructions for running the application.

Referee #2

(Remarks to the Author)
I want to thank the authors for thoroughly addressing both my and reviewer 1's comments. The authors have sufficiently addressed my concerns and answered my questions.

(Remarks on code availability)

Referee #3

(Remarks to the Author)
A strong concern is with the use of the terms "foundation model" and "virtual tissue." To my mind, "virtual tissue" should only be used for a model that captures all or the most important properties of a tissue. This should include being able to generate new examples of the tissue de novo including all of the specific structures. Otherwise it is just a predictive model for a specific property or properties.

A central concern is then whether VirTues provides an advance over current approaches for each of the tasks it addresses. In other words, if contemplating how to analyze data from a new project, would one expect better results from VirTues or a specialized tool or tools? The discussion provides some comments regarding limitations, but the impression that is left is that a potential user would need to carry out their own comparison to other tools. For example, the previous review pointed out the issue of reliance on visual evaluation. New results are presented in Figures S12 and S12 to address this, but those results show large variability in performance for different markers and tissues. They do not give confidence that future uses of VirTues would be reliable for a chosen application.

There is also little methodological innovation since it uses well established methods.

My overall opinion therefore is that, while the work presented is extensive and interesting, it is not of sufficient significance for publication in Nature.

(Remarks on code availability)
I have examined the GitHub repo and it appears to be complete, including adequate instructions for installing and running. I did not attempt to install and run the code.

Version 3:

Reviewer comments:

Referee #1

(Remarks to the Author)
The authors have addressed the questions I raised previously. Thank you.

(Remarks on code availability)
The code provides a README file with instructions for running the application.

Referee #2

(Remarks to the Author)
The revisions, including the expanded analyses on a much larger dataset, as well the stated commitment to share this dataset and publicly, strengthen the value of this work for the under-addressed area of spatial proteomics.

(Remarks on code availability)

Referee #3

(Remarks to the Author)
The manuscript has been extensively revised in response to the reviews. Most significantly, the training data of their system has been dramatically expanded and the authors have demonstrated that the system outperforms existing systems for cell segmentation and typing. The authors demonstrate strong practical utility by using VirTues to develop biomarkers that outperform current markers for predicting patient outcomes. VirTues is therefore anticipated to be remarkably useful to the community.

(Remarks on code availability)
I have examined the code repo and found it to be well documented, including Jupyter notebooks for using pretrained models. I have not attempted to install and run the code.

Open Access This Peer Review File is licensed under a Creative Commons Attribution 4.0 International License, which permits use, sharing, adaptation, distribution and reproduction in any medium or format, as long as you give appropriate credit to the original author(s) and the source, provide a link to the Creative Commons license, and indicate if changes were made.
In cases where reviewers are anonymous, credit should be given to 'Anonymous Referee' and the source.
The images or other third party material in this Peer Review File are included in the article’s Creative Commons license, unless indicated otherwise in a credit line to the material. If material is not included in the article’s Creative Commons license and your intended use is not permitted by statutory regulation or exceeds the permitted use, you will need to obtain permission directly from the copyright holder.
To view a copy of this license, visit https://creativecommons.org/licenses/by/4.0/

Responses to Reviewers

We thank all reviewers for their careful and constructive assessment. The feedback was very helpful in sharpening the scope and strengthening the evidence, in particular by motivating new experiments on generalization beyond a single technology and a deeper analysis of zero-shot behavior. We updated the Introduction, Results, Discussion, Methods, and Supplement accordingly.

For readability, figures that appear only in this rebuttal are labeled Fig. R# and may be referenced across reviewer responses. Figures labeled Fig. # and Fig. S# refer to figures in the revised main manuscript and revised Supplementary Material, respectively.

Response to Reviewer 1

We thank the reviewer for the careful and constructive assessment and for concrete suggestions that materially strengthened the manuscript. We implemented a **substantial revision** that adds cross-technology experiments (IMC, CODEX, and Orion), expands the zero-shot section with marker-level analyses and validation of virtual staining for cell phenotyping, and adds additional validation and statistical tests for the clinical and biomarker results. Below, we address each point in detail and indicate where changes were incorporated into the revised manuscript and Supplement.

> General comments:
Overall, this manuscript presents an ambitious foundation-model approach for spatial proteomics and demonstrates promising performance across multiple downstream tasks using IMC data. Several methodological and conceptual limitations currently reduce the strength of the conclusions. Key concerns include limited evidence of generalizability beyond IMC platforms, incomplete descriptions in marker selection and reporting, and marginal performance improvement in clinically critical settings such as pretreatment pCR prediction. Clarification of modeling choices, evaluation protocols, and biological assumptions could further strengthen rigor, interpretability, and translational relevance.

In response to these overarching concerns, we made three major updates.

- **Generalizability beyond IMC:** We added a new cross-technology evaluation spanning IMC, CODEX, and Orion, covering both (i) multi-technology training of VirTues on a pooled corpus and (ii) strict IMC-only training followed by zero-shot transfer to CODEX and Orion. The revised results not only show that the Virtual Tissues platform (VirTues) is the **best spatial proteomics foundation model across all technologies**, but also that it is the *only* model that robustly generalizes across all technologies. Strikingly, VirTues trained on only IMC still outperforms or is on par with multi-technology competitors, demonstrating **strong zero-shot capabilities** under substantial marker-panel and technology shifts.
- **Zero-shot analysis and validation:** We expanded the evaluation of VirTues' zero-shot marker reconstruction with additional experiments and new datasets across technologies, including an analysis linking reconstruction degradation to marker similarity. We further show that downstream tasks and cell-type phenotyping (including rare populations) remain largely stable, even when phenotyping is performed from markers that are virtually stained by VirTues.
- **Clinical and biomarker evaluation checks:** We added additional validation and statistical tests, and clarified the evaluation protocol where needed for the clinical and biomarker results.

We will release trained models for IMC and across the new technologies, as well as a dataset overview and the splits of the multi-technology corpus (Suppl. Tab. S1 and S2).

> 1. This manuscript focuses exclusively on IMC imaging techniques in developing the VirTues model. However, the generalizability of VirTues to other widely used multiplexed immunofluorescence (mIF) approaches, such as Cycif, Codex, and MIBI, remains unknown. These techniques often provide higher spatial resolution and more detailed characterization of the tumor microenvironment.

We thank the reviewer for raising this point. We agree that demonstrating robustness beyond a single spatial proteomics technology is important for a foundation model intended for broad use. To address it directly, we have conducted **extensive new experiments on an expanded multi-technology corpus** comprising CODEX, Orion, and IMC technologies (Fig. R1a). We want to emphasize that **we designed the VirTues architecture to be technology-agnostic**. Consequently, we were able to apply VirTues to this cross-technology data corpus without any technical adaptations, reimplementation, or architectural changes.

Specifically, we augmented the original IMC data corpus with public spatial proteomics datasets comprising seven CODEX studies (156k crops incl. data from Schürch et al., 2020; Lin et al., 2023; Ruf et al., 2023; Phillips et al., 2021; Monkman et al., 2024; Hickey et al., 2023; HuBMAP Consortium, 2023) and one Orion study (417k crops incl. data from Lin et al., 2023) in addition to the 15 IMC datasets (247k crops) used in the original manuscript (Fig. R1a). Using this expanded multi-technology dataset, we trained VirTues (multi-technology) jointly on the pooled corpus with the same implementation and training recipe as in the manuscript. We compared it to VirTues (IMC-only) as well as KRONOS and CA-MAE and evaluated both tissue- and cell-level prediction tasks across all three technologies (CODEX, Orion, and IMC). We summarize the main outcomes below:

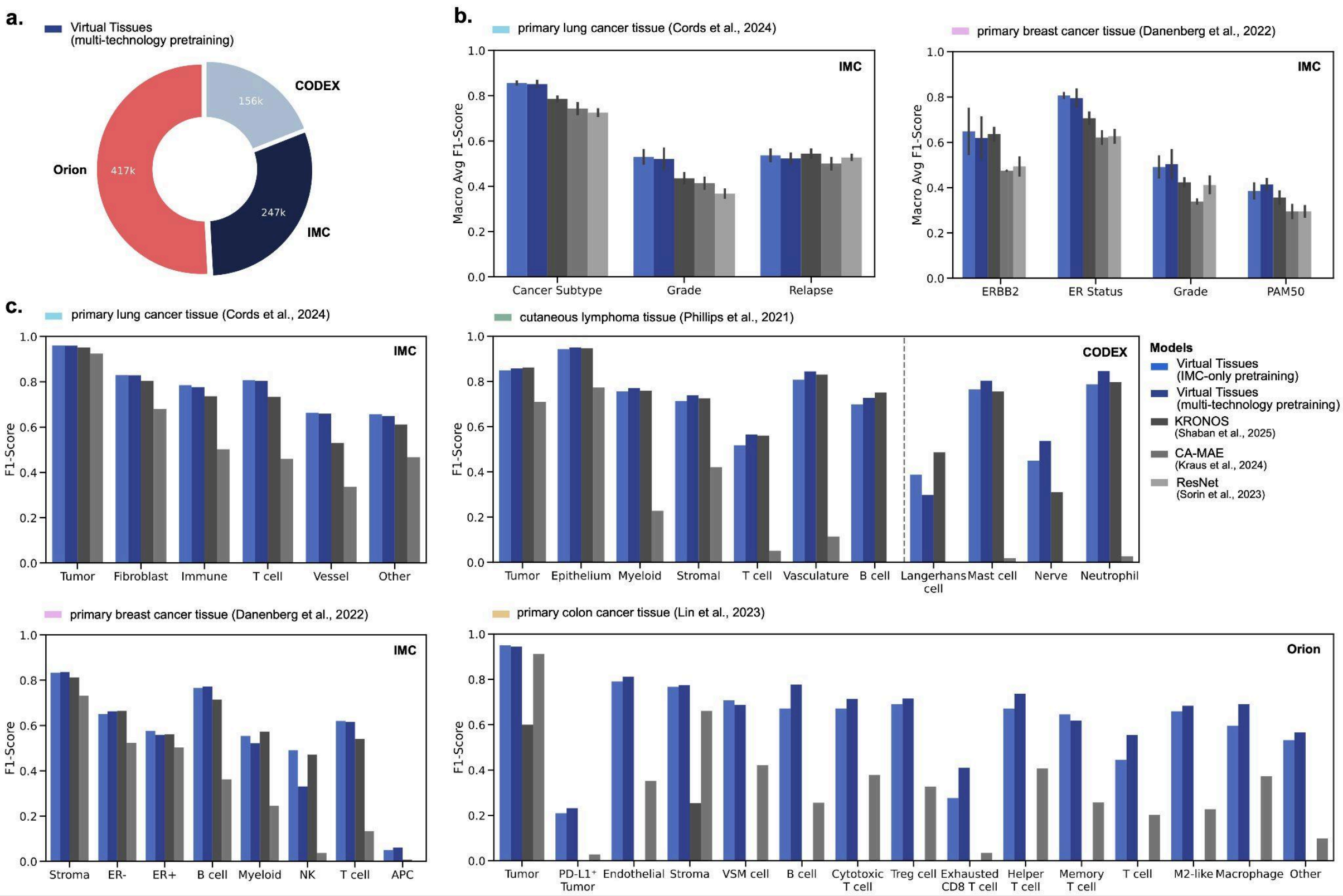

Figure R1. Application of VirTues to imaging technologies beyond IMC. **a,** Number of image crops per technology in the extended data corpus comprising 23 datasets. **b–c,** Comparison of downstream performance between VirTues (IMC-only), VirTues (multi-technology), and baseline methods. **b,** Macro-averaged F1 scores for tissue-level prediction on the IMC datasets from Cords et al. (2024) and Danenberg et al. (2022). **c,** F1 scores for cell-type classification on IMC datasets from Danenberg et al. (2022) and Cords et al. (2024), the CODEX dataset from Phillips et al. (2021), and the Orion dataset from Lin et al. (2023). For Phillips et al. (2022), a dashed gray line separates rare cell types, defined as classes with fewer than 100 test cells (out of n = 27,739 total test cells). For the IMC datasets, we report results of a custom instance of KRONOS trained on our IMC data collection. For CODEX and Orion datasets, we utilise the model released by Shaban et al. (2025).

1. **VirTues achieves state-of-the-art performance across all technologies.** For cell type classification in the cutaneous lymphoma CODEX dataset (Phillips et al., 2021), VirTues (multi-technology) achieves state-of-the-art performance with a macro-averaged F1 score of 0.662 (Fig. R1c), surpassing KRONOS (0.649 macro-F1) by 1.9%.
   Similarly, on the colorectal cancer Orion dataset (Lin et al., 2023), VirTues (multi-technology) attains leading performance in cell type classification (0.661 macro-F1) outperforming both KRONOS and CA-MAE by a substantial margin.
   On the tissue-level and cell type classification prediction tasks on two IMC datasets (Cords et al., 2024, Danenberg et al., 2022), VirTues (multi-technology) achieves close-to-on-par performance to the best performing VirTues (IMC-only) (Fig. R1b,c) and again outperforms KRONOS and CA-MAE.
2. **Critically, VirTues trained exclusively on IMC data shows strong zero-shot generalization to unseen technologies.** VirTues (IMC-only) shows strong zero-shot cell phenotyping performance on CODEX (Phillips et al., 2021) and Orion (Lin et al., 2023) datasets, achieving macro-F1 scores of 0.639 and 0.619 respectively. Notably, approaching the performance of the multi-technology model despite never encountering either technology during training. Beyond, this zero-shot performance substantially exceeds KRONOS, which performs substantially worse even though it was trained on proprietary Orion data (Shaban et al., 2025; Suppl. Tab. 1).

To summarize, the results show that **VirTues is the best performing spatial proteomics foundation model across CODEX, Orion, and IMC** when trained on the pooled multi-technology corpus. Most strikingly, an **IMC-only trained VirTues model exhibits strong zero-shot transfer**, performing well on CODEX and Orion despite never seeing these technologies during training. In contrast, competing methods tend to perform well on only a subset of technologies and do not generalize consistently across platforms. We hypothesize that this strong zero-shot transfer arises because VirTues learns technology-invariant spatial representations from multi-marker context and thus captures transferable biological signal, whereas KRONOS relies more on marker-specific, low-plex patch pretraining that is more sensitive to platform-dependent imaging statistics.

We have added the cross-technology experiments to the revised manuscript (Fig. S21, Suppl. Table 2) and discuss the implications for broader spatial proteomics applications in the revised Discussion. Further, we will release trained IMC-only and multi-technology models for the curated multi-technology data corpus (Suppl. Table. 2).

> 2. The datasets used in this study include more than 40 markers. Several of them, such as ER, PR, HER2, PD-L1, GZMB, play critical roles in tumor-immune interactions. The authors could more explicitly justify their strategy for selecting these 40 markers and address potential selection bias to support the robustness of their findings.

We thank the reviewer for this comment. We believe our description of the data collection and marker handling may have been misunderstood. The study does not rely on a manually selected set of “40 markers” (except from the technical scaling analysis in Fig. 1e). Instead, all cohorts were obtained from previously published studies, and the marker panels were defined by the original authors for their specific experimental and clinical questions. For training and evaluation, we used all available markers provided in each dataset, with only a rare consolidation step when multiple channels corresponded to the same protein target (for example, H3 and pH3), in which case we retained a single representative channel to avoid redundancy. We have revised the Methods to make this procedure explicit.

Across all datasets VirTues is trained and evaluated on 147 distinct targets (gene symbols listed in Fig. 1b). This includes ESR1 (ER), PGR (PR), ERBB2 (HER2), CD274 (PD-L1), and GZMB. These markers were included in multiple cohorts because they are commonly assayed in the underlying studies, not because we selected them for their biological relevance.

We would like to emphasize that VirTues can integrate spatial proteomics datasets with any combination or subsets of markers, including new markers not present in training (see the zero-shot results and remarks below), as our architecture does not rely on a fixed panel but on a marker-agnostic design. In contrast, all competitor methods are tied to a fixed marker panel and require retraining and/or architectural changes when the panel changes or new markers are introduced.

> 3. Similar concerns apply to the zero-shot marker reconstruction experiments. Not all evaluated markers are reported in the main text or supplemental materials.

For the Rigamonti et al. (2024) dataset used in the zero-shot marker reconstruction experiments, we retained the complete marker panel from that study, comprising all 27 measured protein and RNA channels. Fig. 2h reported reconstruction performance separately for each of these 27 markers, including the 20 markers present in the training set and the 7 markers evaluated zero-shot. So no marker from the original study was omitted. During our revision, however, we decided to exclude MS4A4 from further analysis (see Response 5 for further details).

> 4. The performance of cell phenotype prediction appears limited when applied to previously unseen markers, particularly for rare cell populations that may play essential roles in the TME. This limitation may restrict the model's utility for discovering novel niches or rare but biologically important cell types.

We respectfully disagree with the reviewer's assessment that VirTues' cell phenotyping performance is limited when applied to previously unseen markers. In the manuscript, zero-shot cell phenotyping is evaluated in Fig. 3d across four independent benchmarks in which each dataset contains markers not observed during training: three in Cords et al. (2024), four in Danenberg et al. (2022), 13 in Hoch et al. (2022), and eight in Wang et al. (2023). Despite the introduction of these previously unseen markers, zero-shot performance remains stable relative to the in-domain setting, with negligible degradation across datasets (maximum Δmacro-F1 ≤ 0.028).

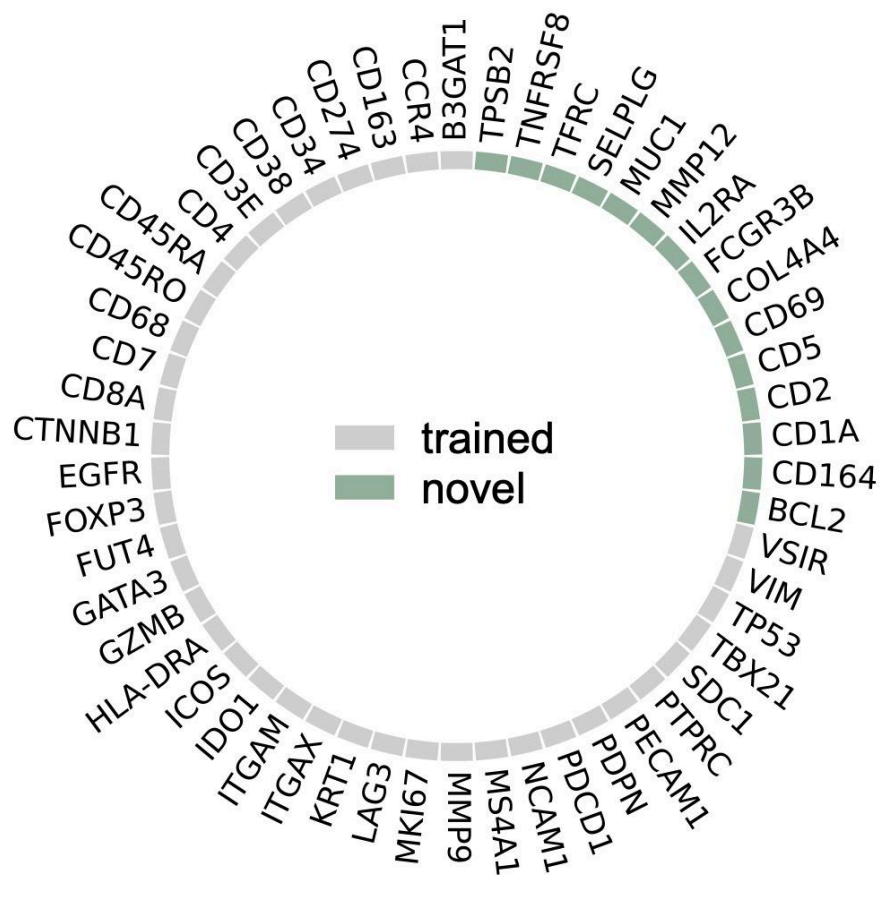

Figure R2. Overview of markers imaged in the CODEX dataset of Phillips et al. (2021). Markers shown in grey were included in the IMC pretraining datasets seen by VirTues (IMC-only), whereas markers shown in green are novel.

Moreover, the new cross-technology zero-shot phenotyping results on the CODEX cohort of Phillips et al. (2021) using an IMC-only model (Fig. R1) further support VirTues' ability to phenotype cells under substantial marker-panel shift and unseen markers (see Response 1 for details). Specifically, in Phillips et al. (2021) 15 out of the 54 markers were not present in the IMC training corpus (Fig. R2), VirTues (IMC-only) achieved strong cell classification performance with a macro-averaged F1-score of 0.639, close to VirTues (multi-technology) and maintains high accuracy on two extremely rare cell type populations: mast cells (F1=0.765; ~0.53% of cells) and neutrophils (F1=0.787; ~0.37% of cells).

Finally, in the Response to Reviewer 2 (Remark 2), we have further added an experiment where we analyze VirTues' ability to annotate cell types when **key lineage markers are not measured but replaced by VirTues-reconstructed (virtual) channels.** As suggested by Fig. R8, VirTues' annotations remain largely stable across B and T cell settings except in the hardest case where multiple lineage-defining markers are simultaneously removed (Fig. R8, Fig. S8). These results show that VirTues supports robust cell phenotyping under unseen marker conditions, cross-technology panel shifts, and even partially virtual marker panels.

> 5. Zero-shot marker reconstruction performance is sometimes limited and can underperform simple baselines, such as the average or the most highly correlated marker, especially for new markers. This limits the model's generalization to cohorts with different biomarker panels and suggests the possibility of shortcut learning. A detailed error analysis would strengthen this section.

We appreciate the reviewer's comment on this point. In the following, we provide a more detailed evaluation on VirTues' zero-shot reconstruction performance as well as an extended analysis for which markers zero-shot performance reduces.

For clarity, we distinguish two levels of difficulty for zero-shot marker reconstruction: (1) reconstructing markers that were seen during pretraining, but evaluated on a held-out clinical study that was not included in pretraining (dataset-level zero-shot), and (2) reconstructing markers that were never encountered during pretraining and evaluated on a held-out clinical study (marker-level and dataset-level zero-shot).

In the first setting, reconstruction performance remains strong. As shown in Fig. 2h, under independent and niche masking, the drop in performance compared to the in-domain setting is minimal ($\Delta r = 0.016$ resp. $\Delta r = -0.002$). Under marker masking, performance decreases more noticeably ($\Delta r = 0.129$), but the overall correlation remains high (avg. $r = 0.667$) and VirTues outperforms both baselines (average and highest correlating marker) on 15 out of 19 markers. Overall, these results indicate that VirTues transfers robustly to unseen studies when the target markers are within the pretrained vocabulary.

For novel markers, we acknowledge that zero-shot reconstruction under marker masking is lower than in-domain ($\Delta r = 0.286$), with substantial marker-to-marker variability. VirTues achieves high correlations for ARG1, C1QA, and CD63, whereas reconstruction of CEACAM1, MS4A4A, and S100A8 remains limited. This limitation is specific to the hardest setting (marker masking), where the entire channel is missing. Under independent and niche masking, reconstruction remains close to in-domain levels, including for CEACAM1 and S100A8, and independent masking consistently outperforms niche masking. This suggests that when any within-channel signal is available (independent masking), VirTues can leverage it to infer the target marker, whereas marker masking isolates the fully missing-channel case.

In line with the reviewer's suggestion, we conducted an **analysis to understand when zero-shot reconstruction is expected to perform well versus when it is likely to fail**. In doing so, we observed the following trend: zero-shot reconstruction performs best when the target marker has close analogs among proteins seen during pretraining, corresponding to strong local support in the pretrained protein representation space. (Fig. R7; see also our response to Reviewer 2, Question 1). Concretely, zero-shot markers located in denser neighborhoods of pretrained markers (that is, proteins whose sequences are close to many proteins seen during pretraining) tend to be reconstructed better, whereas markers in sparse neighborhoods (few or no nearby pretrained proteins) tend to be reconstructed worse. In particular, CEACAM1 and S100A8 (Fig. 2h, Fig. R7a) lie among the sparsest neighborhoods, consistent with their limited reconstruction performance. To provide additional evidence, we repeated the same analysis on an independent zero-shot cohort (Phillips et al., 2021) and observed a similar relationship (Fig. R7b), indicating that the trend is *not* cohort-specific. We have added the analysis to Fig. S17 and updated the manuscript.

This behavior is not consistent with shortcut learning, where performance would be dominated by copying a single highly correlated marker or reverting to a cohort-level mean and would therefore be largely insensitive to the target marker's identity. Instead, performance varies systematically with marker identity in a way that is predicted by sequence-level similarity to the pretrained marker vocabulary, and the same dependence is reproduced across cohorts.

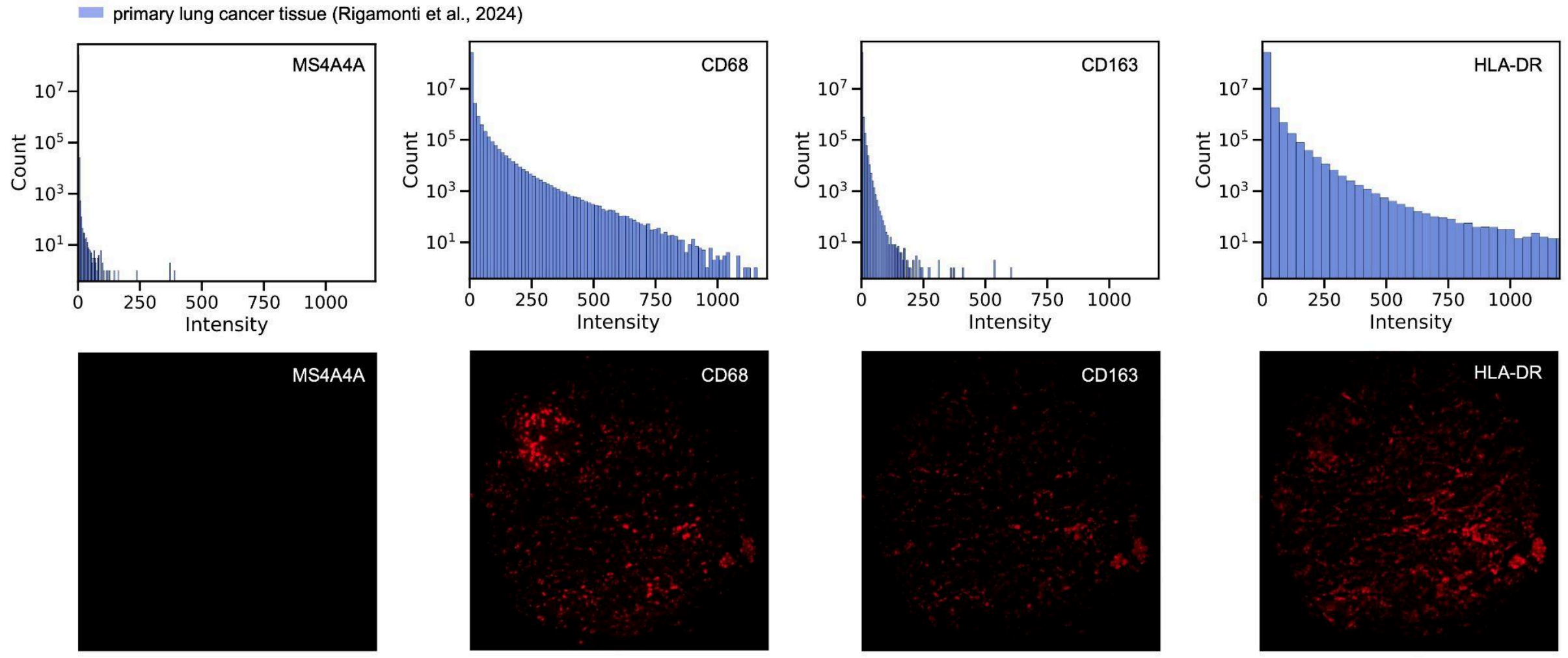

Figure R3. Comparison of intensity distributions of MS4A4A and CD68, CD163, and HLA-DR across all samples of Rigamonti et al. dataset (top row), with corresponding representative sample channels (bottom row).

Lastly, during this extended analysis, we realized that for MS4A4A, the measured channel exhibits extremely low and sparse signal intensities relative to other macrophage markers (Fig. R3). Upon reviewing the original publication, we found that this marker had been deliberately excluded from the original analysis (Suppl. Table 3 of Rigamonti et al., 2024). We therefore suspect a measurement failure and excluded MS4A4A from our analysis as well.

Importantly, the reconstruction limits discussed here do not prevent effective downstream use on novel cohorts with different panels. For example, virtual biomarkers discovered in the TNBC cohort of Wang et al. (2023) transfer to an independent breast cancer cohort (Meyer et al., 2025) despite only 19 overlapping markers between panels, improving patient risk stratification and supporting broad generalization to new marker panels (Fig. 5). Further, as multiplex panels continue to expand and additional public cohorts are released, the coverage of the pretrained marker vocabulary will naturally broaden, reducing the frequency of entirely out-of-vocabulary markers in this reconstruction setting.

> 6. For pathological complete response (pCR) prediction in Fig. 4f, the manuscript reports results from five runs using different random seeds. Five-fold cross-validation would be preferable to improve statistical power and reliability. In addition, VirTues underperforms CA-MAE for pretreatment TNBC pCR prediction, which is arguably the most clinically relevant time point for informing early treatment decisions.

We agree that cross-validation can increase statistical power. However, the primary goal of Fig. 4f is to compare embedding methods under a strict out-of-sample setting where the evaluation tissues are *not seen during pretraining*. Performing 5-fold cross-validation over the full dataset would place some pretraining tissues into evaluation folds, which would confound the comparison by partially measuring familiarity with pretraining samples rather than representation quality on held-out tissues. We therefore used the same fixed 80:20 split as in pretraining and

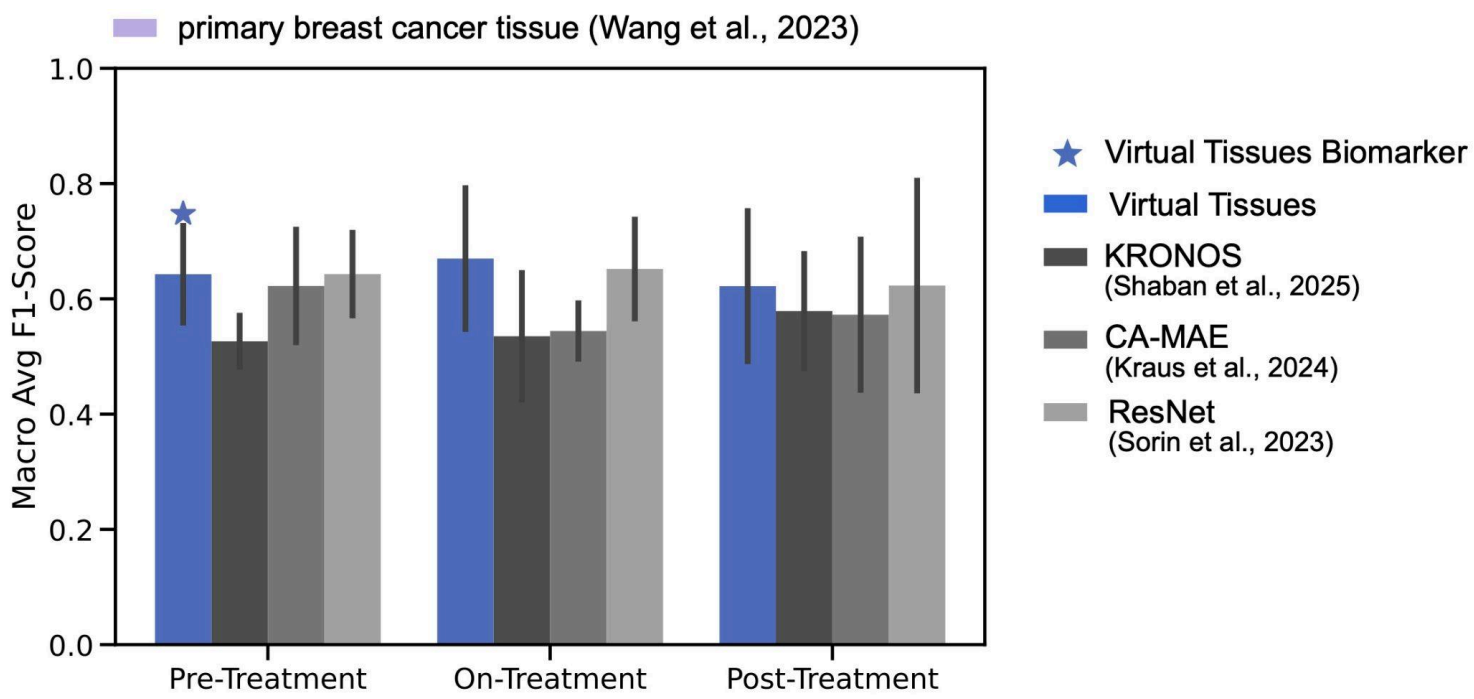

Figure R4. Macro-averaged tissue-level F1 scores for response prediction on the Wang et al. (2024) dataset using 5-fold cross-validation. Error bars indicate the standard deviation across folds. For reference, the predictive performance of the foundation model-based biomarkers on pre-treatment samples is indicated by a star.

reported variability across five random seeds, so that Fig. 4f reflects embedding quality on tissues held out from pretraining. Since the hold-out test sets of Cords et al. (2024) and Dannenberg et al. (2022) are large, n>300 and n>110, respectively, the setup provides stable out-of-sample estimates. Wang et al. (2023), however, contains substantially fewer patients and therefore yields a much smaller held-out test set (e.g., n=60 for pre-treatment), making performance estimates under a single 80:20 split less stable.

To address the reviewer's concern regarding the statistical significance of our conclusions, we nonetheless repeated the experiments on Wang et al. (2023) using 5-fold cross-validation. As shown in Fig. R4, the results exhibit trends consistent with those reported in the manuscript, with VirTues performing better than or on par with the other methods, though always within the error range of the runner up.

Importantly, Fig. 4f is intended as a controlled benchmark to compare embedding methods under a strict held-out-from-pretraining evaluation, rather than as a quantification of a final output from a clinical prediction pipeline. Accordingly, we developed the FM-derived biomarker framework of Fig. 5, since it provides an interpretable patient stratification and achieves substantially stronger performance (AUROC ~0.82 in Fig. 5e; macro-F1 ~0.75 in Fig. R4, star). There, we followed the repeated cross-validation protocol of Wang et al. (2023) to ensure comparability with the original study. We then validate these biomarkers on an independent TNBC cohort (zero-shot, not present in pretraining; Meyer et al., 2025), where they improve patient risk stratification (Fig. 5j).

> 7. Regarding the identification of foundation-model-based biomarkers, the authors could clarify the rationale for the chosen number of repetitions (e.g., 10 repetitions for cluster rank selection and 25 for joint prediction). Do these experiments share the same data split across folds? In addition, the comparison across biomarkers is limited. The authors could strengthen this analysis by expanding the reference biomarker set and including an additional baseline that uses tabular spatial single-cell data to replicate the same workflow.

We thank the reviewer for this suggestion and clarify the rationale and protocol used for the FM-derived biomarker analysis. Our goal was to follow the evaluation procedure of Wang et al. (2023) as closely as possible, both for statistical reliability and to enable a fair comparison to their reported biomarkers and workflow. To this end, we used repeated, stratified 4-fold cross-validation, with 10 repetitions for cluster ranking and selection and 25 repetitions for the joint prediction setting, where exact comparability to Wang et al. (2023) is required. In practice, we found that the results are not sensitive to the exact number of repetitions at this scale; 10 repetitions are sufficient to yield stable cluster rankings, and increasing repetitions only marginally changes estimates (ΔAUROC < 0.002).
During revision, we further audited the precise cross-validation scheme used by Wang et al. (2023) for the joint prediction setting and realized that our initial description did not fully match their protocol. We therefore repeated the joint prediction analysis using 100 random 75/25 train-test splits (instead of 25 repetitions of 4-fold cross-validation), aligned with Wang et al. (2023), to assess joint performance. This correction had a negligible, slightly positive effect on the reported AUROC (0.818 → 0.823). We updated the Results, Methods, and Figures accordingly. In the rebuttal, we also double-checked that indeed the cluster-ranking experiments and the joint prediction evaluation use distinct data splits.

Further, we thank the reviewer for the suggestion to include an additional baseline based on tabular spatial single-cell data. We note, however, that this comparison is already covered by the tabular spatial predictor of Wang et al. (2023). A core contribution of that work is the construction of a feature set (148 tissue-level features) that explicitly encodes cell phenotypes, marker positivities, and spatial cell-cell interactions, followed by correlation-based feature reduction and integration into a state-of-the-art predictor. Because our FM-derived biomarkers substantially outperform this established tabular spatial baseline, we believe that adding an additional tabular baseline that follows a similar workflow would be unlikely to change the conclusions or materially strengthen the analysis.

> 8. Ground truth inaccuracy: The authors should discuss the potential sources of error or uncertainty in the cell-type annotations, as this may affect the validity of performance evaluations. Providing refined or updated cell phenotype labels would be a valuable contribution to the research community.

We agree with the reviewer, and thank them for this spot-on remark. In this study, we rely on cell phenotype annotations provided by the original authors of the respective publications. These labels are typically produced by panel-adapted pipelines that combine iterative clustering, gating strategies, manual assignment, and simple machine learning models on tabular single-cell features; each step possibly introducing error or uncertainty.

In fact, during quality control of Cords et al. (2024), we identified a substantial fraction of clearly misannotated cells, which initially manifested as an unexpected drop in apparent prediction performance of VirTues. We therefore asked an expert to re-annotate using a reference subset and used it to correct labels at scale; in total, 14.6% of cells were relabeled. We therefore treat these annotations as imperfect ground truth, which necessarily skews performance. To ensure comparative methodological conclusions, all compared methods are trained and evaluated on the same labels.

The reviewer's remark underlines a central ambition and purpose of VirTues: to move beyond brittle, study-specific phenotyping pipelines and toward consistent, reusable tissue representations that support cross-cohort analysis and automated cell typing. Since VirTues provides a shared embedding space across datasets with different marker panels and different capture technologies, it can transfer cell labels to entirely new cohorts without requiring retraining or access to labels. We show that this is possible in the label-free, zero-shot label transfer experiments in Fig. 3e-g. In this way, VirTues can serve as an enabling layer for harmonizing cell typing across studies and to building spatial proteomics atlases that are comparable across cohorts, panels, and acquisition platforms. We have added a discussion of this point to the manuscript. As data collection efforts accelerate, our hope is that VirTues will help nucleate a technological rallying point to integrate and enrich spatial proteomics tissue data from which the whole community would profit.

> 9. On page 18, the terminology of "marker attention" and "channel attention" is a bit ambiguous. In the VirTues encoder, the index m appears to be used interchangeably to denote both the m-th channel and the m-th marker. Clearer definitions and notation would improve interpretability.

We have revised the manuscript to clarify terminology and notation and to use it consistently throughout. Specifically, we now refer to attention across spatial tokens within a marker as *spatial attention*, and to attention across markers (channels) at a fixed spatial location as *marker attention* (see Fig. 1d).

Regarding the stand-alone terms "channel" and "marker," we agree that the previous text could suggest they are used interchangeably as each channel in a multiplex image corresponds to exactly one marker. However, we generally aim to use the term "channel" as a technical term for an input image component, and "marker" to denote the biological target measured by that channel. We updated the manuscript to incorporate this more consistently.

> 10. On page 18, the decoder input is described as concatenating non-masked encoded tokens with masked image tokens (i.e., the tokens prior to masking). However, in real zero-shot scenarios, these image tokens are not available. The authors could clarify how masked tokens are decoded in practice.

The confusion likely stems from interpreting "masked image tokens" as the original tokens before masking. In VirTues, however, masked tokens are never passed to the decoder in their original form, i.e., including the underlying image signal. Instead, during masking, we replace the selected patch tokens with a dedicated **mask token**, a learnable vector that is part of the model parameters (see the

Masking subsection). The decoder input therefore consists of (i) the encoded tokens for unmasked patches and (ii) **mask tokens** at the masked positions; “masked image tokens” refer to these mask tokens, not to the pre-mask content, and are thus also available in zero-shot scenarios.

At inference time, the same mechanism applies. When a patch is missing, we insert the mask token at that position and the decoder predicts its content. For zero-shot reconstruction of an entirely missing marker channel, we add a placeholder channel and mark all of its patches as masked, so the encoder receives no information from that channel and the decoder reconstructs it purely from the observed channels and spatial context. We have revised the manuscript to make this decoding procedure explicit.

> 11. For heteromeric proteins, did the authors focus exclusively on the primary signaling chain (e.g., the alpha-chain) while excluding other chains? If so, how might this simplification affect the learned representations? Would the results differ if alternative chains were used (e.g., CD8A versus CD8B)?

For heteromeric proteins (e.g., CD8), we do not assume a “primary” chain. We map each channel to the specific chain targeted by the antibody in the source study (e.g., CD8A vs CD8B). Using an alternative chain would misspecify the marker identity and could add noise; in practice the effect is likely small for closely related, co-expressed subunits, but we avoid this by adhering to antibody specificity.

> 12. Could the authors provide the exact amino acid sequence used as inputs to the ESM-2 model for each biomarker to improve reproducibility?

We apologize for not describing this procedure explicitly. For each marker, we used the canonical protein sequence from UniProt (UniProtKB), mapped from the marker’s gene/protein identifier as specified in the antibody panel descriptions of the original studies. We have updated the manuscript to state this selection procedure. In addition, we have archived all downloaded FASTA files and will include them as Supplementary Material in the final publication.

> 13. In Supplemental Figure S12, using the mean intensity of each marker to separate components may not be optimal. A Gaussian mixture model (GMM) or similar probabilistic approach may yield a more robust separation.

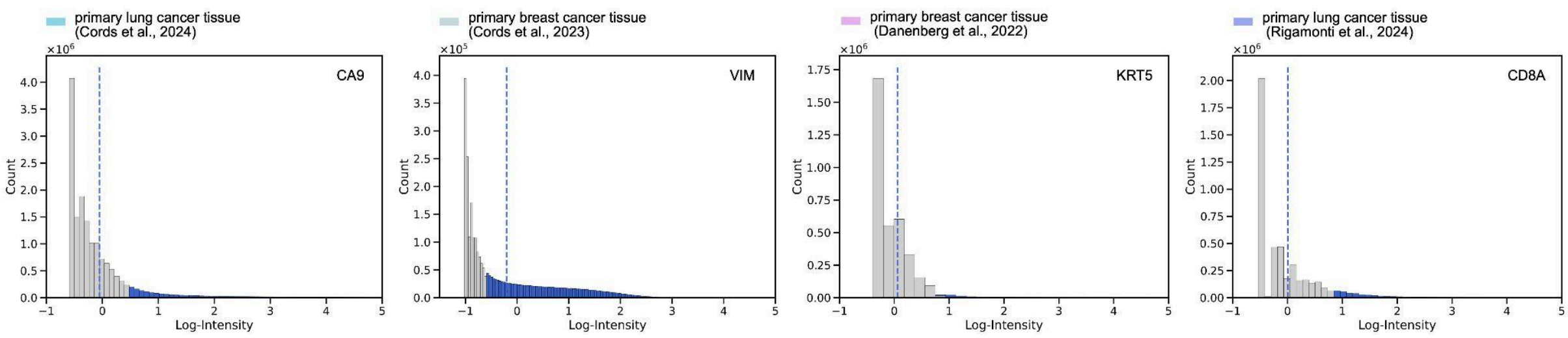

Figure R5. Histograms of log1p-transformed intensities for CA9, VIM, KRT5 and CD8A. The bar colors indicate the positivity assigned by a Gaussian mixture model. The dashed line indicates the mean.

We thank the reviewer for raising this concern. The purpose of Fig. S12 was to introduce a metric complementary to the Pearson correlation that quantifies how accurately VirTues’ reconstructions capture marker positivity. To this end, we classified pixels as positive by applying a marker-wise threshold at the mean intensity and computed the resulting F1-score between predictions and ground truth. Initiated by the reviewer’s comment, we re-evaluated this metric and found that, despite log normalization, marker intensity distributions generally do not exhibit bimodality but instead more closely follow exponential-like distributions. Consequently, neither the mean-based thresholding strategy nor the Gaussian mixture-based stratification proposed by the reviewer impose inherently

arbitrary thresholds for marker positivity (Fig. R5), making the metric overly sensitive to small errors near the threshold.

In light of this limitation, we have replaced this metric with the mean squared error (MSE), computed between standardized true and predicted intensities (see updated Fig. S13 in the revised manuscript). This choice provides a more stable and interpretable evaluation, as an MSE of one corresponds to an error of one standard deviation.

> 14. In Figure 5b, the distributional shifts across pre-, on-, and post-treatment samples are not visually pronounced. Including a formal statistical test would help clarify whether these differences are meaningful.

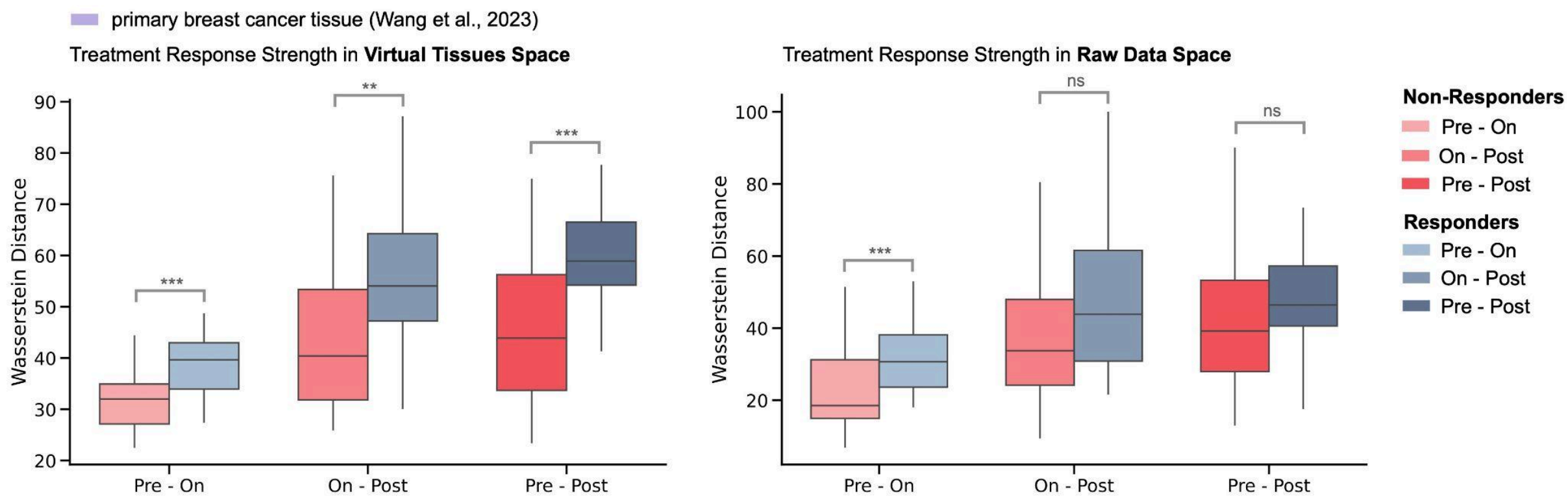

Figure R6. Comparison of treatment response strengths. Patient-level treatment response strength is reported as the Wasserstein distance between cell embeddings, stratified by compared treatment stages, pathological complete response status, and embedding space. Statistical significance tested by a two-sided Mann-Whitney U test is indicated above brackets: $p < 0.05$ (*), $p < 0.01$ (**), $p < 0.001$ (***); ns indicates $p \geq 0.05$.

We agree that a formal statistical test would strengthen our analysis to assess whether the shifts between pre-, on-, and post-treatment samples are meaningful, and we have added such an analysis. Biopsies at different time points are unpaired and unaligned, so treatment-induced change should be quantified by comparing the tissue states of two biopsies without requiring any explicit correspondence. We therefore use a distributional distance, namely the 2-Wasserstein distance, which implicitly aligns the two biopsies in the representation space (either raw marker intensity space or the VirTues embedding space) and yields a single response strength quantifying how much the tissue state has shifted. Concretely, for each patient and each interval (pre-on, on-post, pre-post), we compute the 2-Wasserstein distance between the two biopsies' tissue-state distributions and use two-sided Mann-Whitney U tests to compare response strengths between responders and non-responders for each interval for raw data and VirTues encoded samples.

As shown in Fig. R6, measurements in the Virtual Tissues space reveal significantly higher response strengths in responders compared to non-responders across all treatment stage comparisons (pre-on: p = 7.44e-4, on-post: p = 1.40e-3, pre-post: p = 4.25e-4). In contrast, when computed in the raw marker intensity space, a significant difference between responders and non-responders is observed only for the pre-on comparison, with no significant effects detected for the other treatment intervals (pre-on: p = 2.54e-4, on-post: p = 6.46e-2, pre-post: p = 8.91e-2).

Together, these results statistically substantiate Fig. 5b and show that VirTues embeddings capture treatment-induced shifts in the tumor microenvironment more sensitively and consistently than raw marker-based representations. We updated our manuscript to include this analysis in the Results, Methods and Fig. S26.

> 15. Existing methods, such as GigaTIME[1] and HistoPlexer[2], generate virtual mIF tissues and offer a cost- and time-efficient approach for deriving proteomic information and

characterizing the tumour immune microenvironment. Could the authors clarify how VirTues improves beyond these prior models?

GigaTIME (Valanarasu et al., 2025), HistoPlexer (Andani et al., 2025), and ROSIE (Wu et al., 2025) address virtual staining from H&E. VirTues is instead a **general-purpose foundation model for spatial proteomics**. While it can impute missing spatial proteomics markers from an observed subset of spatial proteomics markers, its primary goal is broader: to provide a reusable virtual tissue representation that supports a broad set of downstream tasks on any new dataset independent of the panel design, including cell, niche, and tissue annotation, among others. Furthermore, these representations enable a novel way of patient stratification and biomarker discovery.

As a further distinction, the above methods are restricted to a fixed, predefined marker set and do not support inference on previously unseen markers. Concretely, ROSIE, GigaTIME, and HistoPlexer predict expression for fixed panels of 50, 21, and 11 markers, respectively. Extending them to a new marker would require collecting paired supervision for that marker and retraining the model. VirTues in its current version supports 146 or 213 markers for IMC-only or across all technologies respectively, is designed to operate under marker shifts and can handle new markers via its marker-aware architecture.

While we believe that virtual staining methods from H&E will improve in the future, we would like to emphasize that their accuracy, especially at the single-cell level, is inherently limited by the lower information content of H&E compared to spatial proteomics. This is evident from the results reported across all studies Both GigaTIME and ROSIE report, except from trivial morphology-related markers (e.g., DAPI, Cytokeratin or EpCAM), per-marker Pearson correlations mostly in the low range of 0.1 to 0.4 (c.f. Figure 2B, Wu et al. (2025) or Figure 2B, Valanaruasu et al. (2025). HistoPlexer lacks interpretable bounded metrics, but the qualitative prediction examples likewise show limited single-cell accuracy (c.f. Figure 2a, CD8a or CD20, Andani et. al, 2025).

> Additional minor points: 1. Page 9 Line 284: There is a typographical error (“the the”) in the sentence.

We thank the reviewer for pointing out this error, which we have corrected in the revised manuscript.

Response to Reviewer 2

We thank the reviewer for the thorough and constructive assessment. The comments helped us sharpen the presentation and strengthen the evidence in several key places.

Before addressing the specific remarks, we briefly highlight a central outcome of the revision prompted by Reviewer 1, since it also directly informs several of Reviewer 2's concerns about generalization, marker-panel shift, and the biological usefulness of zero-shot virtual staining. Concretely, we expanded the analysis with a **new cross-technology evaluation spanning IMC, CODEX, and Orio**n, covering both (i) multi-technology training of VirTues on a pooled corpus and (ii) strict IMC-only training followed by zero-shot transfer to CODEX and Orion. The revised results not only show that the Virtual Tissues platform (VirTues) is the **best spatial proteomics foundation model across all technologies**, but also that it is the **only model that robustly generalizes across all technologies**. Strikingly, VirTues trained on only IMC still outperforms or is on par with multi-technology competitors, demonstrating **strong zero-shot capabilities** under substantial marker-panel and technology shifts.
This cross-technology expansion provides a direct test of generalization beyond a single platform and also underpins the new analyses prompted by the reviewer's remarks that assess zero-shot behavior and downstream phenotyping stability. We refer to these results where relevant throughout the responses below (new Fig. R1 and associated Supplementary material).

We now provide point-by-point responses below.

> Questions:

1. Zero-shot marker reconstruction: it is reasonable to assume that the performance of the new markers in the zero-shot reconstruction case would be a function of their similarity or functional relationship to markers in the training set. Figure 2f seems to be an attempt at showing this, but the UMAP is far from helpful in this regard. Do you see any evidence for such a function? Whether or not you do, it would be helpful to comment on this in the paper.

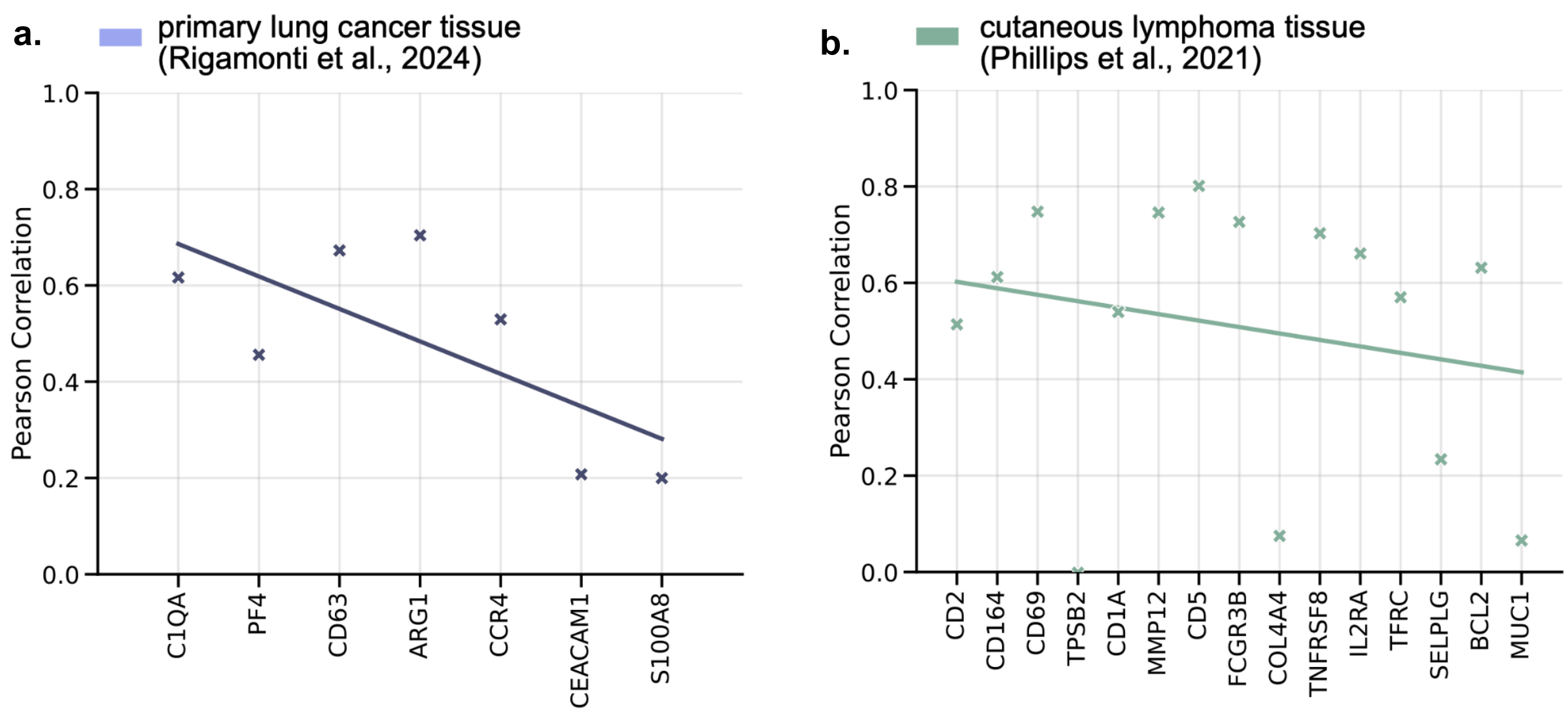

Figure R7. Reconstruction performance under marker masking measured by Pearson correlation in the zero-shot setting for novel markers on **a,** the IMC dataset of Rigamonti et al. (2024) and **b,** the CODEX dataset of Phillips et al. (2021). Markers are ordered from left to right by increasing average L2 distance to their three nearest training-markers in ESM space. Solid lines indicate a linear regression fit.

We thank the reviewer for raising this point. We agree with the underlying hypothesis: zero-shot reconstruction quality for a novel marker should depend on its similarity to markers encountered

during pretraining. We also agree that a UMAP visualization is not well suited to establish such a relationship, since it is qualitative and can distort distances.

As part of the rebuttal, we therefore conducted a quantitative analysis of how marker similarity relates to zero-shot reconstruction performance. We use local density in ESM embedding space as a proxy for similarity, that is, whether a zero-shot marker lies in a "dense" neighborhood of markers seen during pretraining (many nearby proteins in sequence space) or in a "sparse" neighborhood (few or no close pretrained neighbors). Concretely, for each zero-shot marker we compute the average L2 distance between its ESM embedding and those of its k nearest training markers (k = 3), and relate this distance to the achieved Pearson correlation. Across Rigamonti et al. (2024) zero-shot markers (Fig. 2e-h, Fig. R7a) and in an additional independent zero-shot cohort newly added in this revision (Phillips et al., 2021, CODEX) (Fig. R7b), we observe that markers with closer pretrained neighborhoods tend to achieve higher reconstruction correlations, whereas markers in sparse neighborhoods tend to be reconstructed less well (Fig. R7). We have added this quantitative analysis and an explicit discussion of the observed trend to the revised manuscript (Results and Fig. S17).

We would like to stress that **this evidence should be interpreted with appropriate caution** given the limited number of zero-shot markers per dataset and the remaining marker-to-marker variability.

> 2. The many appeals to reconstructions being visually plausible are hard to judge, even for experts. Visually plausible and biologically valid are far from the same thing. Is there a way you could provide evidence for the biological validity of the reconstructions? E.g., do inferred annotations remain the same between the original and inpainted images?

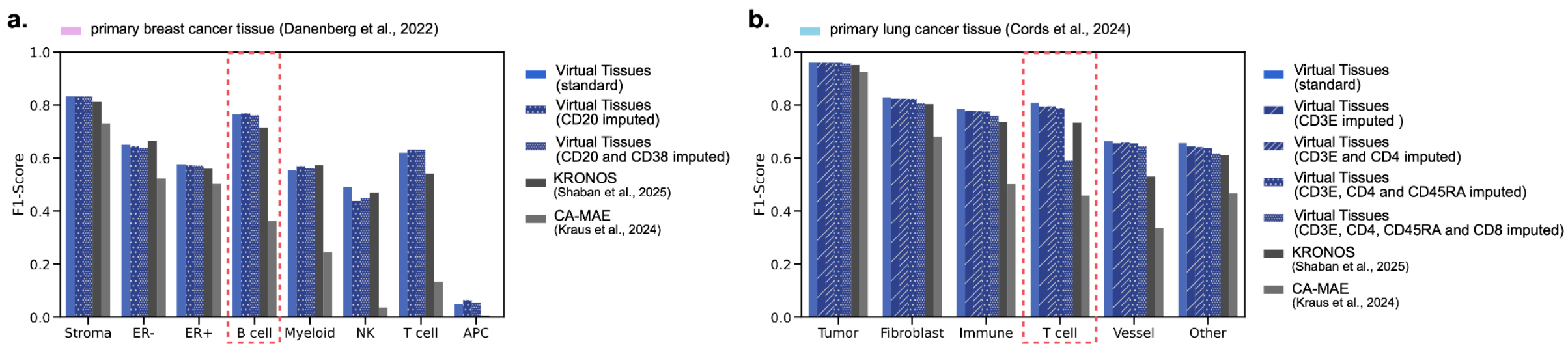

Figure R8. Evaluation of virtual staining for downstream cell phenotyping. VirTues is used to reconstruct subsets of markers, generating virtual stains that are incorporated into a partially virtual multiplex image for cell phenotyping. Performance is reported using class-wise F1 scores. For reference, the performance of VirTues, KRONOS, and CA-MAE using the full multiplex image, based on all real channels, is also shown. **a,** On Danenberg et al. (2022) we reconstruct (i) CD20 and (ii) CD20 and CD38. Classification results for B cells, which are primarily identified by these markers, are highlighted. **b,** On Cords et al. (2024) we reconstruct (i) CD3E, (ii) CD3E and CD4, (iii) CD3E, CD4 and CD45RA and (iv) CD3E, CD4, CD45RA and CD8. Classification results for T cells, which are primarily identified by these markers, are highlighted.

We thank the reviewer for raising this important concern. We agree that visual plausibility alone is insufficient to establish biological validity. Accordingly, the manuscript emphasizes (i) **quantitative reconstruction metrics comparing pixel-wise prediction to ground truth**, including held-out pixel Pearson correlation (Fig. S12) and mean-squared error (Fig. S13) under independent (i.e., randomly across the tissue), niche (i.e., for certain regions masked across all markers), and full marker masking (Fig. 2a-c), (ii) comparisons to baselines such as mean imputation and predicting a marker from its most correlated training marker (Fig. 2d, Fig. S12 and S13), and (iii) stringent zero-shot evaluation on unseen markers measured by Pearson correlation and MSE (Fig. 2e-h, S14, S15, S16).

We also thank the reviewer for suggesting an **evaluation directly at the level of downstream biological interpretations**. To this end, we tested whether cell type annotations remain stable when measured markers are replaced by reconstructed, virtual markers. Specifically, on Danenberg et al. (2022), we sequentially removed the canonical B cell markers, first CD20, then both CD20 and CD38, reconstructed them with VirTues, and then re-ran cell phenotyping on the resulting partially virtual image set. As shown in Fig. R8a, B cell classification is essentially unchanged when using

reconstructed CD20 and CD38 compared to the standard setting, indicating that the reconstructed markers preserve the biologically relevant signal needed for phenotyping. Phenotyping performance for other major cell types using the same, partially virtual image set, was largely stable, and VirTues remains competitive relative to other methods (KRONOS, CA-MAE), even when those are evaluated with fully measured panels.

On Cords et al. (2024), we performed an analogous analysis for T cell identification by progressively excluding key T cell markers with increasing difficulty (CD3E only; CD3E+CD4; CD3E+CD4+CD45RA; CD3E+CD4+CD45RA+CD8), reconstructing the missing channels using VirTues, and re-running phenotyping. T cell F1 remains close to the standard setting when virtually staining only CD3E, CD3E+CD4 or CD3E+CD4+CD45RA, and degrades as the fourth lineage-defining marker CD8 is removed (Fig. R8b). Notably, performance remains above CA-MAE across all settings and only drops below KRONOS (which has access to all measured markers) once all four markers must be reconstructed. CD8 marks cytotoxic T cells, a lineage whose spatial distribution and activation state are only weakly constrained by morphology. In Cords et al. (2024), CD8+ cells account for 65.3% of all T cells (according to annotations by Cords et al., 2024), and in this panel CD8 is the only direct lineage-defining marker for that majority. Removing CD8 on top of CD3E, CD4, and CD45RA, and without any remaining cytotoxic effector markers in the panel, therefore removes most decisive factors that the model would leverage for reconstruction, explaining the drop in overall T cell phenotyping performance (while still achieving a strong F1 score of ~0.6 given the level of difficulty).

We thank the reviewer again for suggesting these informative experiments that provide direct evidence that VirTues' reconstructions remain biologically actionable for downstream cell typing, and we have added this analysis to the revised manuscript (Fig. S8).

> 3. Why weren't KRONOS and CA-MAE embeddings used for comparison in the patient stratification task?

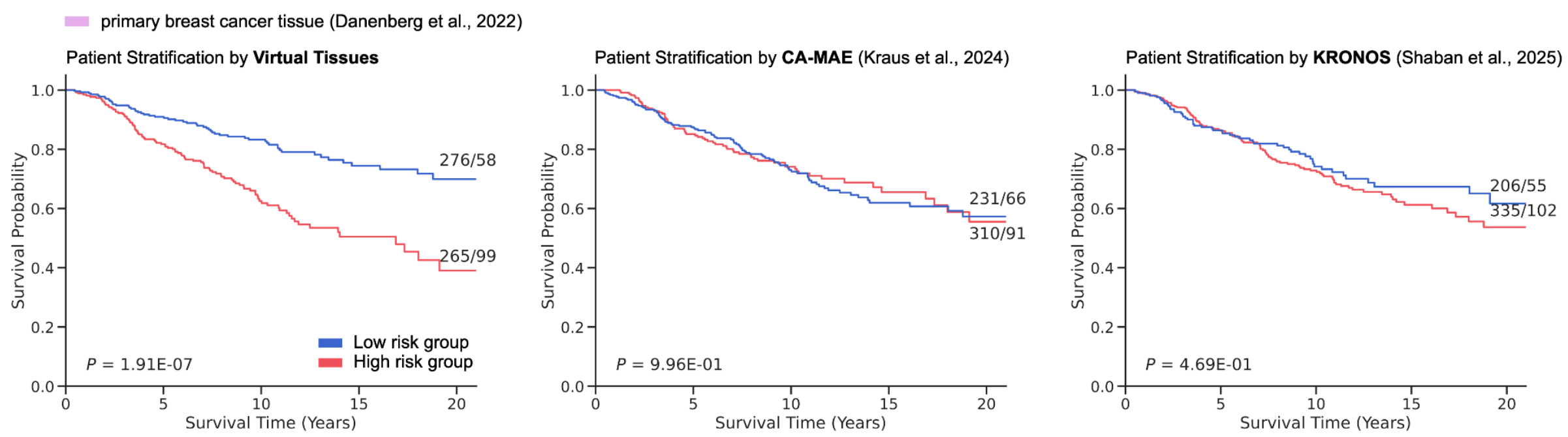

Figure R9. Comparison of patient survival stratifications based on VirTues, CA-MAE, and KRONOS. For each method, the *p*-value from a log-rank test comparing the two survival curves is reported.

The primary objective of the patient stratification analysis was to compare VirTues against the hypothesis-driven spatial proteomics literature. In Danenberg et al. (2022), survival-associated microenvironments were identified via a manual, hypothesis-driven workflow that explicitly annotated niches across tissues and linked them to patient risk through hazard ratios (Danenberg et al., 2022, Fig. 6). VirTues instead enables hypothesis-free stratification into high- and low-risk groups using its learned virtual tissue representations (Fig. 4a). We showed that this yields a strong risk stratification (Fig. 4c) and that the resulting split is explained by the presence of the same types of prognostic microenvironments in the VirTues-derived groups (Fig. 4d).

As requested by the reviewer, however, we repeated the identical stratification pipeline using KRONOS and CA-MAE embeddings, and reported the corresponding survival curves in Fig. R9. In this setting, neither baseline yields a statistically significant risk stratification. A plausible explanation

is that CA-MAE, by design, is less suited to integrate the high-plex contextual information needed to capture microenvironment architecture at scale, while KRONOS (DINOv2-based) emphasizes global instance-level invariances and yields less contextualized neighborhood representations than our masked reconstruction objective. In addition, KRONOS representations appear more panel-dependent, something we also observe in the label-free cell-type transfer experiment, where KRONOS exhibits weaker cross-cohort robustness than VirTues (Fig. 3e-g). KRONOS' panel sensitivity propagates to noisier representations of local tissue organization, which is particularly detrimental here as prognostic signal is driven by higher-order tumor microenvironment architecture (spatial interactions and neighborhood structure), not merely coarse cell-type abundance. By contrast, VirTues is explicitly trained to align across heterogeneous panels by conditioning on marker identity and learning contextualized patch neighborhoods, which likely preserves the tissue-architecture signal required for prognostic stratification in this cohort.

> 4. So many of these tasks require assigning image patches to cells and tissues, which is done using software other than VirTues. How do these results depend on the choice of assignment? And why not train a cell and tissue assignment model together with VirTues?

We thank the reviewer for this question. VirTues itself does not require segmentation. The model operates on patch tokens and produces patch-level representations from which most core outputs can be computed directly, including marker reconstruction and niche- and tissue-level representations via simple aggregation.

Tissue masks are used purely for coarse quality control and preprocessing: we retain crops with at least 30% tissue coverage and compute dataset-wide preprocessing statistics on tissue pixels. These masks are straightforward to obtain with standard image processing (Otsu thresholding with hole filling; see revised “Dataset curation” in Methods), and because they are only used as a coverage filter, we do not expect results to be sensitive to the precise tissue-masking procedure. We therefore see no need for an additional learned tissue segmentation model.

For cell-level tasks, we use the cell segmentations provided by the original studies, together with their published cell labels and clinical annotations. VirTues cell embeddings are computed by overlap-weighted aggregation of patch representations, which reduces sensitivity to minor boundary differences compared to hard assignment. Subsequently, all methods are evaluated using the same provided segmentations and labels, so the comparative results are not driven by a VirTues-specific segmentation choice.

We did not train a joint segmentation model with VirTues because segmentation is a separate, well-studied problem with strong existing specialized solutions. In practice, the most promising path to improve segmentation accuracy is likely a dedicated multimodal approach that leverages complementary cues (for example morphology from H&E stains together with multiplex signals), which is outside the scope of this work.

> 5. How are niche summary tokens produced? The text says they are “trained to capture tissue composition, architecture and interactions between different cellular neighborhoods” but I don't see an explanation of this in the text.

Both niche-level and tissue-level summary tokens are obtained by aggregating the corresponding encoded patch summary tokens. For supervised downstream tasks, we employ an attention-based aggregation mechanism that is learned via fine-tuning. In contrast, for unsupervised settings, such as the retrieval experiment, we use simple averaging as the aggregation strategy. We specified the exact details of these procedures in the paragraph ‘Aggregation into cell-, niche- and tissue-level representations’ of the Methods section.

> 6. Why do you use all tokens when calculating reconstruction metrics, rather than just the masked one? It is far more common to report the latter, and avoids the bias resulting from including "reconstructed" unmasked tokens.

We believe there might be a misunderstanding between the training objective and the evaluation protocol. During training, we compute the reconstruction loss (MSE) over all pixels for stability. In contrast, all reconstruction evaluation metrics are computed exclusively on the withheld signal, that is, on pixels corresponding to masked tokens only. Specifically, Pearson correlations are reported on masked pixels (Fig. 2d,h; Fig. S12), and MSE is likewise computed on masked pixels (Fig. S13; replacing the previous positivity F1, see Response 13 to Reviewer 1). We have clarified this distinction in the "Masked reconstruction" paragraph of the Methods section.

> 7. The use of OT-based approaches for tissue retrieval is done without comment or explanation. Do the results change if another distributional-distance measure is used? Please explain why this and not other means of measuring token similarity was chosen.

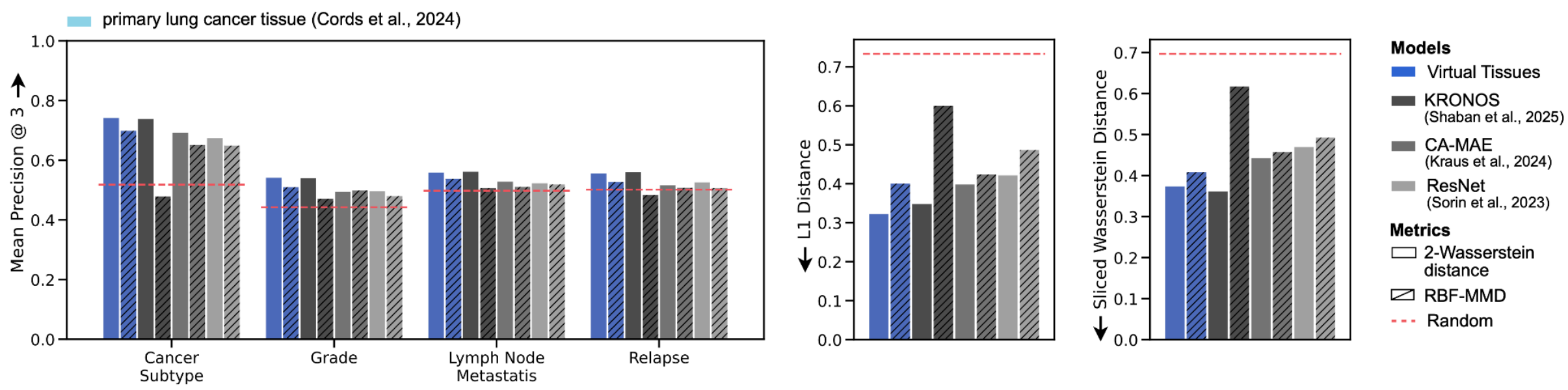

Figure R10. Ablation of distance metrics for tissue retrieval. For each embedding method, we compare the 2-Wasserstein distance and the RBF-MMD distance averaged over length scales [1, 0.5, 0.1, 0.01, 0.005] in terms of (left to right): (i) correctness of retrieved tissue annotations, (ii) similarity of cell phenotype compositions measured by the L1 distance between normalized histograms, and (iii) molecular similarity measured by the sliced Wasserstein distance between pixel intensities. Red dashed lines indicate scores attained by uniformly random retrieval.

We apologize that our motivation for using an OT-based distance for tissue retrieval was not explained clearly in the original submission.

In this experiment, we want retrieval to surface patients with similar molecular tissue phenotypes. Since tissues are heterogeneous mixtures of distinct microenvironmental states, we represent each patient by a small set of niche summary tokens rather than collapsing everything into a single pooled vector that can dilute clinically relevant states. Comparing two patients therefore requires a distributional notion of similarity between two small point sets of tokens.

We use the 2-Wasserstein (optimal transport) distance because it provides a principled metric between empirical distributions with potentially distinct supports and remains meaningful in the small-sample regime. As requested by the reviewer, we additionally compared against an alternative distributional distance, RBF-kernel MMD (averaged over length scales [1, 0.5, 0.1, 0.01, 0.005]). Across all evaluations and embedding models, MMD consistently yielded lower retrieval performance than 2-Wasserstein (Fig. R10), supporting our choice of OT. We have updated the Methods section to include this motivation and justify the metric distance selection.

> 8. Please comment on why single-cell morphology models like scDINO (Pfaendler et al., 2023) or Doron et al., (2023) were not included with KRONOS and CA-MAE as competitive baselines. Did you try to build representations from such models, and if so, what were the results?

We did not include scDINO (Pfaendler et al., 2023) or Doron et al. (2023), both based on DINO-trained vision transformer backbones, because they are not technically comparable to our setting without substantial, non-standard adaptations that would confound the benchmark. Concretely, like standard DINO formulations, they assume object-centric, fixed-channel inputs and

are typically used via a single global embedding per image, which does not transfer cleanly to highly multiplexed, multi-cell tissue tiles with variable marker panels. Instead, we add a comparison to the DINOv2-based baseline KRONOS, which has been explicitly adapted to multiplex spatial proteomics inputs, making it the appropriate DINO-family baseline for our evaluation.

More specifically, scDINO (Pfaendler et al., 2023) and Doron et al. (2023) are designed for single-cell morphology learning from high-resolution (~0.32 μm/px), low-plex (3-5 channel) fluorescence images cropped and centered on individual cells, whereas our objective is multi-scale tissue representation learning from lower-resolution (1 μm/px), highly multiplexed images (30+ channels) with intrinsically multi-cell tissue architecture. As mentioned above, both scDINO (Pfaendler et al., 2023) and Doron et al. (2023) use a DINO-style objective that, in standard usage, provides a single global embedding per input. On tissue tiles, as the case for spatial proteomics however, pooling into one vector mixes multiple cellular phenotypes and does not yield cell-level representations without an additional, method-specific cropping and aggregation pipeline. Instead, more recent DINO-family developments such as iBOT and DINOv2 are better suited to dense, multi-object imagery, and KRONOS indeed builds on these developments in a form tailored to multiplex spatial proteomics. Lastly, scDINO (Pfaendler et al., 2023) and Doron et al. (2023) assume a fixed number and ordering of input channels, which is incompatible with the varying marker panels across our cohorts and thus prevents cross-dataset training and inference.

> Suggestions:

1. Using “virtual” in the name of this model is possibly problematic as recently the term has come to refer to models that predict the outcomes of interventions on the data. In this paper, VirTues is used only to represent data, and each application either 1) compares / clusters embeddings, or 2) classifies / annotates embeddings, and lacks any in silico simulation of interventions. I recommend the following:
- Add such an application, e.g. something similar to the T-cell effector state simulation reported in this blog post by Noetik [3].
- Address the recent virtual cell literature more directly (e.g. the oft-cited Cell perspective by Bunne et al.) and describe how VirTues fits into it.

We thank the reviewer for this thoughtful point and the concrete suggestions. The “oft-cited Cell Perspective by Bunne et al.” is indeed very close to home: The last author of the present work is also the first author of that Perspective, and VirTues was developed to implement the design principles articulated there. We have now made this connection explicit in the revised manuscript, and we regret that it was not clearly stated previously.

First, we agree that “virtual” is increasingly used in the context of *in silico* interventions. At the same time, both the broader literature and the Bunne et al. (2024) Perspective use “virtual” more generally to denote learned, *in silico* representations of biological states that can be queried, compared, and composed across heterogeneous data. In this manuscript, “Virtual Tissues” refers to such virtual tissue states that encode molecular and microenvironmental tissue organization from spatial proteomics into a patient-level representation capturing cell identities, microenvironment compositions and organizations, and as a combination of those, tissue states.

Second, while VirTues is not a mechanistic perturbation simulator, we do observe that the learned virtual tissue states already support monitoring and disentangling tissue changes under treatment, which is a prerequisite for intervention modeling. Concretely, in the NeoTRIP clinical cohort (Wang et al., 2023), VirTues embeddings (unlike analyses on the raw marker space) clearly separate responders from non-responders, consistent with responders exhibiting pronounced treatment-induced shifts in tissue state while non-responders change only marginally (Fig. 5b). As part of our revisions, we further validated that these shifts are statistically significant for VirTues-derived embeddings but not for raw data using two-sided Mann-Whitney U tests (Fig. R6

and Fig. S26). The intervention-style “virtual perturbation” examples such as the Noetik blog’s T cell effector state simulations are indeed compelling illustrations, but so far not accompanied by perturbation-ground-truth validation in the same data. We therefore position perturbation simulation as a natural downstream extension building on VirTues’ tissue state representations, and we add this point and the relevant references to the Discussion.

> 2. It is incorrect to refer to the pretrained model as multi-scale. It is trained at a single scale (128 x 128 crop of original image), and only the embeddings are aggregated (sometimes via learning) at various scales to provide representations of cells, niches, tissues, and patients. A true multiscale model would be trained using inputs at multiple scales, not derive them after the fact.

We thank the reviewer for pointing this out and agree with the distinction. VirTues is trained at a fixed spatial patch resolution (128 × 128 crops), and the multi-scale aspect arises from how patch representations are subsequently aggregated (sometimes via learning) into cell-, niche-, tissue-, and patient-level representations. Our intended meaning was therefore that VirTues provides a foundation for analyses and predictions across multiple biological scales, rather than that it is a multi-resolution input architecture.
Concretely, VirTues supports marker reconstruction at the subcellular scale, cell phenotyping at the cellular scale, niche discovery and retrieval at the niche scale, cancer typing and grading at the tissue scale, and risk and response stratification at the patient level. In addition, VirTues conditions on molecular inputs via protein sequence embeddings (from a frozen protein language model encoder) that are passed through learnable projection layers, so model outputs depend on both marker identity at the molecular level and spatial tissue patches at the subcellular level.

To avoid ambiguity, we will revise the manuscript and, where appropriate, replace “multi-scale pretrained model” with wording such as “a foundation model enabling multi-scale representations and predictions”.

> 3. I think it can be misleading to call the proposed encoder attention scheme “sparse” - it is factorized into alternating layers of dense channel-only and spatial-only attention blocks. This is not a terribly important point, but I would recommend referring to this as factorized / alternating attention rather than sparse.

We thank the reviewer for this remark but politely disagree that the term “sparse” is inappropriate. Sparse attention is a common term in deep learning research referring to variants of the attention mechanism, in which given N tokens only a sparse subquadratic subset of the N^2 pairwise attention scores are computed. In our setting, with $N=CHW$ tokens, this criterion is satisfied for both attention components: spatial attention computes $C(HW)^2$ pairwise attention scores, while marker attention computes $C^2\,HW$ pairwise scores.

> 4. Unlike KRONOS, CA-MAE was not purposefully designed for applications like spatial proteomics. While it is fair to make comparisons with CA-MAE since it is one of the few vision models that is intended to encode multiplex cellular microscopy images, it is also fair to mention that the intended application of CA-MAE was encoding Cell Painting and Brightfield images.

We thank the reviewer for this comment and have clarified in the revised manuscript that CA-MAE was primarily designed for Cell Painting and brightfield images, rather than highly multiplexed spatial proteomics. We included it here as it is a potential architecture for building FMs for spatial proteomics and we wanted to thoroughly assess architecture choices for VirTues.

> 5. The section titled “Spatial and marker attention provides explainability of algorithmic decisions” is an interesting examination of the connection of markers and learned attention

weights, but it is at best unclear what "algorithmic decisions" is referring to, since there are no decisions made, algorithmic or not, either in this section or in the rest of the paper. I recommend clarifying what is meant by this phrase with examples, or rewording the section and title to more accurately reflect the scope of the results.

We thank the reviewer for pointing this out. We agree that the phrase "algorithmic decisions" is too broad and can be interpreted as referring to discrete clinical decision-making, whereas the analysis in this section focuses on model-internal attribution signals.

Our intent was to show that VirTues can provide interpretable cues about which spatial regions and which markers most influence representations as well as predictions for downstream tasks. To avoid ambiguity, we have revised the section title and wording to reflect this scope more precisely. Concretely, we replaced "algorithmic decisions" with "model predictions" or "model outputs" throughout and renamed the section to "Spatial and marker attention provide explainability of model outputs". We also added a brief sentence to clarify which predictions the attention maps are intended to help interpret.

> 6. The tree diagrams in Figure 5g are visually harder to interpret than simple bar graphs, I recommend changing these.

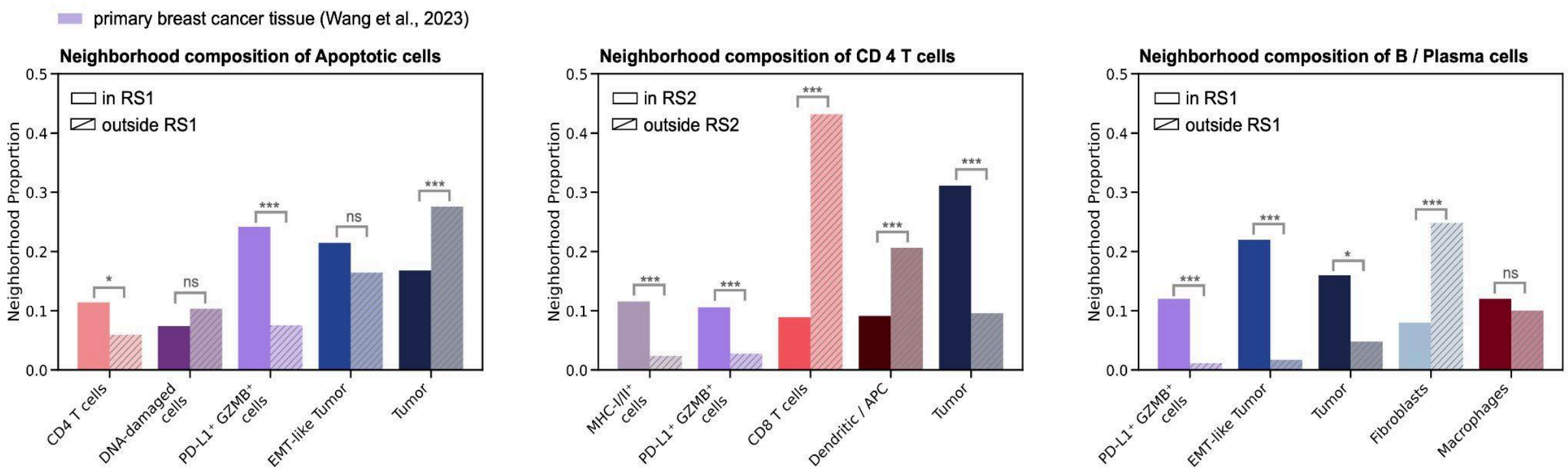

Figure R11. Comparison of neighborhood densities of selected cell types inside and outside the response signatures (RS). Bar diagrams show, from left to right, neighborhood composition of apoptotic cells inside versus outside RS1, CD4 T cells inside versus outside RS2, and B / Plasma cells inside versus outside RS1. Only the five most frequent neighboring cell types are shown. Statistical significance of the differences between inside and outside the signature tested by a proportions z-test is indicated above brackets: $p < 0.05$ (*), $p < 0.01$ (**), $p < 0.001$ (***); ns indicates $p \geq 0.05$.

We thank the reviewer for this remark. Please find above a bar graph visualization of Fig. 5g. For now we have included them as an alternative visualization in the Supplementary Material (Fig. R11, Fig. S28) as we find the current edition of tree diagrams easier to read. But if requested by the reviewer, we are happy to exchange them.

Responses to Reviewers

We thank the reviewers for a careful and constructive assessment that has strengthened the manuscript. The central additions in this revision are twofold: an extension of VirTues' capabilities as well as expanding the benchmark of VirTues against the single-purpose tools that spatial proteomics analysis currently relies on. We have revised the Results, Methods, and Supplement accordingly, and added six new figures.

Reviewers 1 and 3 raise questions that, by different routes, touch on the quality of current ground truth annotations. Reviewer 3 asked whether VirTues is a concrete advance over specialized tools. Across nine datasets spanning different tissues, marker panels, and imaging technologies, all withheld from training, **VirTues performs cell segmentation and cell typing** in a single zero-shot pass and **more accurately than every state-of-the-art tool** we tested (new Figs. 3, R2, S24 and S25). In several of these datasets, its **predictions match the marker signal more faithfully than the published reference annotations**, which are built from clustering and marker-wise gating (new Figs. R3, R4, S23 and S26). Reviewer 1 asked why VirTues scores lower on a few difficult cell types. Direct examination of those cases shows the lower scores partly reflect ambiguity in the gating-derived reference annotations for those rare populations rather than a limitation of VirTues (Figs. R1 and S22).
Because segmentation and cell typing sit at the beginning of every spatial proteomics analysis, the improvement here carries through to every downstream result, and **a single model now does the work of a multi-tool pipeline assembled and tuned for each study**. The additional analysis of the rebuttal complements the **capabilities** already established in the original manuscript, **such as automated cross-cohort biomarker discovery**, which specialized tools cannot provide.

Separately, in response to Reviewer 1's request for accessible phenotype labels, **we will release a harmonized spatial proteomics corpus** of **more than 12,500 multiplexed images** from **over 5,200 patients across 31 cohorts** and **4 imaging technologies** spanning **250 protein markers**, with all original and derived cell type annotations in a standardized format. This is the **largest such resource to date**, and we expect it to accelerate method development across the field.

For readability, figures that appear in this rebuttal are labeled Fig. R# and may be referenced across reviewer responses. Figures labeled Fig. # and Fig. S# refer to figures in the revised main manuscript and revised Supplementary Material, respectively.

Response to Reviewer 1

The authors have addressed the majority of our concerns. The revised manuscript shows substantial improvements, particularly in the standardization of terminology and the enhanced demonstration of generalizability across multiplexed imaging platforms, including CODEX and Orion.

We thank the reviewer for their continued careful engagement with the manuscript and for the positive assessment of the revisions. We address each of the remaining points in detail below.

> 1. Regarding the newly integrated CODEX and Orion datasets, some of these datasets provide tabular single-cell expression matrices rather than curated phenotype labels. The current method, which references general gating and clustering procedures, lacks sufficient detail to ensure reproducibility. In addition, making the curated single-cell phenotype labels publicly available would greatly benefit the spatial proteomics community.

We appreciate the reviewer's comment. We would like to clarify that all cell phenotyping tasks, including those related to the newly incorporated CODEX and Orion datasets from Phillips et al. (2021) and Lin et al. (2023), are based on annotations provided by the original studies. Specifically, Phillips et al. (2021) report explicit fine-grained phenotype annotations, which we consolidated into 11 classes. Lin et al. (2023) provide binary cell-wise marker positivity values, which we mapped to their cell type annotations using the rule-based gating framework described in their Extended Data Fig. 1d (Lin et al., 2023). We have revised the manuscript to include a description of these data curation steps in the Methods section, along with the mapping used for Phillips et al. (2021) (Suppl. Tab. S7).

We agree that accessible phenotype labels would benefit the spatial proteomics community. Therefore, **we will make the collected data corpus, including all original and derived annotations, publicly available** (subject to a small number of licensing restrictions) in a fully standardized format optimized for machine learning applications. We expect this resource to improve reproducibility and to support the development of new computational methods across the field.

> 2. Cell phenotyping performance for clinically important biomarkers, such as PD-L1+ and exhausted T cells, remains suboptimal (F1 score < 0.5 in Orion). Given their critical clinical significance and the inherent challenges in establishing reliable ground truth for these cell populations, it would be valuable to provide a high-quality subset of representative cases for visualization. This would better demonstrate the practical applicability of VirTues.

We thank the reviewer for this suggestion. We prepared representative prediction cases for these two classes (Fig. R1, Fig. S22), and examining them shows that the low scores are driven substantially by ambiguity in the ground truth annotations themselves. Phenotyping exhausted CD8+ T cells and PD-L1+ tumor cells is difficult for every method we tested, and the analysis below shows why.

For exhausted CD8 T cells, most misclassifications occur within the broader CD8 T cell compartment (exhausted, memory, and unspecific cytotoxic T cells). Specifically, false negatives are predominantly predicted as unspecific cytotoxic T cells, whereas false positives are mostly annotated as memory T cells (Fig. R1a). The ground truth annotations from Lin et al. (2023) distinguish these subpopulations via gating on marker expression, specifically on CD45RO and PD-1 positivity.

However, the underlying single-cell marker intensities (Fig. R1b) reveal that this procedure is error-prone in various respects: (i) marker positivity thresholds are derived from per-tissue clustering, resulting in inconsistent global cutoffs (ii) the marker intensity distributions do not exhibit clear separations, leading to ambiguous thresholds, and (iii) CD45RO and PD-1 positivity are not mutually exclusive. Consequently, the sequential assignment strategy (with memory T cells annotated prior to exhausted CD8 T cells) likely biases the labels (which serve as ground truth), contributing to a relative underrepresentation of exhausted CD8 T cells ($n_{Exhausted}$ = 2'112 vs n_{Memory} = 25'070) and complicating their accurate recall through a linear probe. Consistent with these observations, qualitative prediction examples show that many apparent false negatives for exhausted CD8 T cells exhibit clear PD-1 positivity and increased spatial proximity to macrophages (Fig. R1d), supporting their plausibility as exhausted CD8 T cells.

Analogously, PD-L1 tumor cells are predominantly misclassified as regular tumor cells (Fig. R1a,e). In Lin et al. (2023), these two tumor states are separated based on PD-L1 positivity. However, inspection of the underlying PD-L1 intensity distributions exposes the same issues as for exhausted CD8 T cells: Positivity thresholds are derived per-tissue, while the marker distributions themselves show no clear natural separation, making the boundary between PD-L1-positive and regular tumor cells inherently ambiguous. The class imbalance ($n_{PD\text{-}L1+}$ = 13'848 vs $n_{PD\text{-}L1-}$ = 640'479) likely further impairs recall of PD-L1 tumor cells. This ambiguity mirrors clinical practice, where PD-L1 positivity itself lacks a consistent definition and shows well-known inter-observer variability between pathologists.

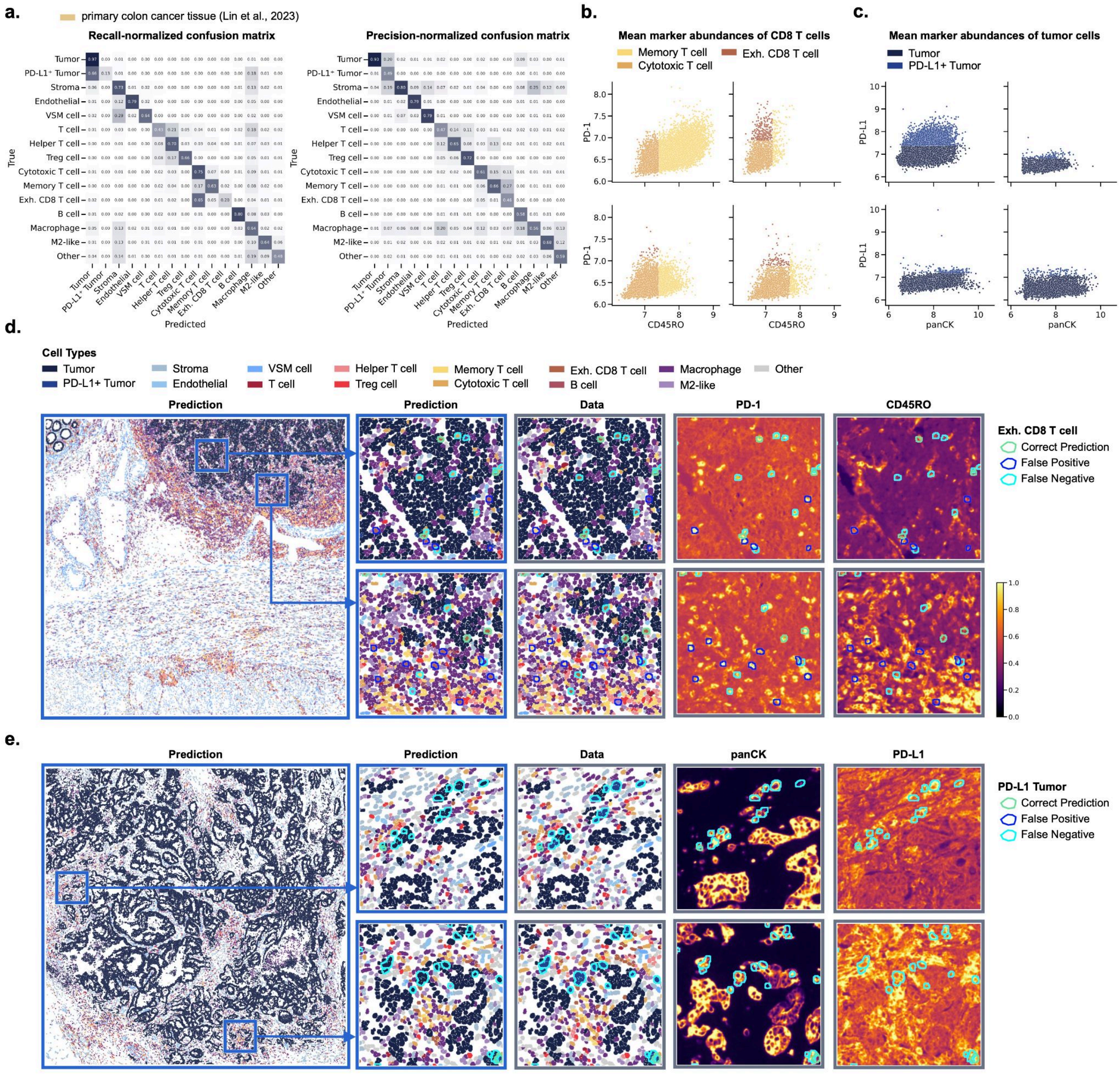

Fig. R1. Cross-technology cell phenotyping performance on Lin et al. (2023). **a,** Recall- and precision-normalized confusion matrices. **b,** Arcsinh-transformed mean-marker abundances for CD8 T cell subpopulations of four different tissues colored by ground-truth annotations. **c,** Arcsinh-transformed mean-marker abundances for tumor cell subpopulations of four different tissues colored by ground-truth annotations. **d-e**, Representative examples of predicted versus ground-truth cell type masks. **d,** Correct predictions, false positives and false negatives for exhausted CD8 T cells are visually highlighted. **e,** Correct predictions, false positives and false negatives for PD-L1 tumor cells are visually highlighted.

This is not an isolated observation unique to the dataset of Lin et al. (2023): In our Response to Reviewer 3, we show across a comprehensive nine-dataset benchmark that VirTues' segmentations and cell type predictions in several cases match the underlying marker signal more faithfully than the published reference annotations (Figs. R3, R4, S23 and S26). The lower F1-scores for PD-L1+ tumor cells and exhausted T cells should be read in that light: they partly measure the quality of the gating-derived ground truth rather than a limitation of VirTues.

> 3. 95% confidence intervals (95% CIs) are missing from several key figures, including Figure 1e; Figures 3b, 3c, 3d, and 3g; Figure 4h; Figure 5k; and multiple panels in the Supplemental Figures. For Figure 5e, DeLong's test should be used for ROC comparison. Additionally, please justify the use of a one-sided Mann-Whitney U test in the tissue-level classification Section on Page 24.

We are thankful for these valuable remarks on our statistical analyses. We have added 95% confidence intervals to the Figs. 1e, 3b-d, 4f, 4h-j, S19 and S21 as well as for the newly added cross-cohort cell typing results shown in Fig. 1g-h, S24 and S25. Confidence intervals were computed via bootstrapping, as the underlying evaluation pipelines are otherwise fully deterministic (fixed train-test split; logistic regression solved using a deterministic L-BFGS optimizer; or similarity-based retrieval). Note that for most cell-level tasks, the resulting confidence intervals are extremely narrow due to the large number of samples, such that the corresponding error bars may be barely visible in the figures. For the newly added cell instance segmentation results, bootstrap resampling at the cell level is technically incompatible with F1-score computation, since it relies on one-to-one matching between predicted and ground-truth cells. On the concordance indices reported in Fig. 5k, meaningful confidence intervals could not be reliably estimated due to the limited cohort size (n = 163) thus precluding statistically robust comparisons of C-indices across methods. Importantly, this does not affect the statistical significance of the patient stratification produced by VirTues itself, which we validated using a log-rank test (P = 0.0037).

Regarding Fig. 5e, we respectfully disagree that DeLong's test is appropriate for this comparison. DeLong's test is designed to compare two paired ROC curves. In Fig. 5e, our goal is not to compare two individual ROC curves but rather the means of two distributions of AUROC values obtained via repeated cross-validation. For this comparison an independent t-test is appropriate, which we have applied.

Lastly, we agree with the reviewer's concern regarding the one-sided Mann-Whitney U test used in tissue-level experiments. A two-sided test is indeed more appropriate, and we have updated our statistical analyses accordingly. Similarly, we revised the aforementioned independent t-test in Fig. 5e to be two-sided.

> 4. Some CIs are not described in the corresponding figure legends (e.g., Figure 4f). In addition, please report exact p-values throughout the manuscript, except in cases where P < 0.001 (e.g., P < 0.1 and P < 0.07 on Pages 8 and 9).

In the revised manuscript, all error bars now consistently represent 95% confidence intervals, and this information has been explicitly added to the corresponding figure legends (including Fig. 4f). In addition, we have revised the manuscript to report exact p-values following the reviewer's instruction.

> 5. Figure 1d visually suggests that VirTues operates on approximately 40 markers; however, the text states that VirTues was trained on more than 140 distinct markers. Please clarify this discrepancy.

We are happy to clarify this discrepancy: VirTues was indeed trained and evaluated on a comprehensive set of 146 distinct markers, corresponding to the union of all marker panels across all IMC datasets (Fig. 1b). In contrast, individual IMC datasets typically measure only ~40 markers or fewer. As a result, for IMC, VirTues was most commonly applied to inputs comprising approximately 40 markers at a time, as illustrated in Fig. 1d.
That said, the cross-technology experiments introduced in the latest revision demonstrate that VirTues generalizes effectively also to larger marker panels at inference time (e.g., 54 markers in Phillips et al., 2021). For this reason and to avoid potential confusion with the total number of markers, we have removed the number of markers from Fig. 1d in the revised manuscript.

> 6. In Figure S19, several bars corresponding to VirTues are missing. Please clarify whether these represent zero values or missing data.

We thank the reviewer for pointing out this ambiguity. In Fig. S19, bars for IDO-CAFs, PDPN-CAFs, dCAFs and hypoxic tpCAFs are missing as these rare cancer-associated fibroblasts do not occur in the test split of the dataset (see Fig. S20 for class counts). For tpCAFs, all methods yield an F1-score of zero. However, this class is also extremely underrepresented (n = 16 out of over one million test cells), which likely accounts for the uniformly poor performance, as the simple logistic regression model lacks sufficient sensitivity to reliably detect such rare populations.

We have updated the figure and revised the caption to explicitly indicate classes with missing test samples.

Response to Reviewer 2

> I want to thank the authors for thoroughly addressing both my and reviewer 1's comments. The authors have sufficiently addressed my concerns and answered my questions.

We thank the reviewer for their careful reading of the manuscript and for their constructive comments throughout the review process. We are grateful that the revisions and our responses addressed the reviewer's concerns. Their feedback helped us clarify the motivation, strengthen the quantitative evaluation, and improve the presentation of the manuscript.

Response to Reviewer 3

> A strong concern is with the use of the terms "foundation model" and "virtual tissue." To my mind, "virtual tissue" should only be used for a model that captures all or the most important properties of a tissue. This should include being able to generate new examples of the tissue de novo including all of the specific structures. Otherwise it is just a predictive model for a specific property or properties.

We thank the reviewer for this comment, an occasion to define both terms more precisely and to extend the work behind them.
We fully agree: a "virtual tissue" model must capture all or the most important properties of a tissue, not predict a single one. VirTues learns its representation through a self-supervised objective, so it can only encode properties present in its data. To capture properties of tissue, rather than of any single dataset, the data must span the diversity of tissues, diseases, and assays. Motivated by the reviewer's question, we have substantially expanded the corpus in this revision to more than 12,500 multiplexed images from over 5,200 patients, across 31 cohorts, 4 imaging technologies, and 250 protein markers. This is the largest and most diverse harmonized spatial proteomics resource to date, and we will release it upon publication. Our results show that the virtual tissue representations capture these properties indeed broadly: The same representation supports reconstruction of held-out protein markers (Fig. 2, Figs. S2-S3), cell segmentation and identity assignment (Figs. 3, R2 and S23-26), tissue region annotation, similar-tissue retrieval, and identification of patient-stratifying biomarkers (Fig. 4-5). This range of capabilities from a single representation, without retraining, is what we mean by a virtual tissue model.

Beyond capturing these properties, the reviewer proposes that a "virtual tissue" model generates new tissue *de novo*. We instead pursue a computational representation of spatially resolved tissue state, which we consider the more meaningful and more impactful objective. A representation can be queried, compared across patients and cohorts, and used directly for prediction and discovery on real

measured samples. *De novo* generation produces synthetic tissue that is hard to validate against ground truth and that does not by itself yield transferable biological or clinical insight. The value in spatial proteomics lies in analyzing tissue from actual patients, and a representation keeps the analysis grounded there. By extension, synthetic tissues are unlikely to reveal meaningful biological and clinical insights unobservable in representation learnt from the same ground truth.

In fact, VirTues follows in the footsteps of histopathology foundation models, which are built and evaluated as representations and reused across downstream tasks rather than as generators of new histology images (Chen et al., 2024). It matches the broader use of "virtual" in the current literature: in the emerging work on virtual cells (Virtual Cells Platform from Biohub; Arc's Virtual Cell Initiative; Leslie, 2025; Bunne et al., 2024; Cheng et al., 2026), "virtual" denotes a computational representation of biological state, structure, and function, not the de novo synthesis of new examples. Our use of "virtual tissue" follows this meaning. We have revised the manuscript to state this definition explicitly and to make clear that VirTues provides a foundation model representation of spatial proteomics data, inevitably incomplete but broadly useful across tasks, rather than a complete simulator of tissue development, morphology, or treatment response.

On "foundation model," we follow the definition of Bommasani et al. (2021): a model trained on broad data, generally with self-supervision at scale, that can be adapted to a wide range of downstream tasks. VirTues meets this definition. It is trained with a task-agnostic self-supervised objective on heterogeneous spatial proteomics data spanning different tissues, diseases, technologies, and marker-panel compositions, and the same pretrained model is used for marker reconstruction, segmentation, cell typing, tissue annotation, retrieval, and spatial biomarker discovery. This places it in the same category as vision foundation models such as DINOv2 (Oquab et al., 2023) and pathology foundation models trained on large H&E corpora (Chen et al., 2024). We have revised the manuscript to make this reasoning explicit.

> A central concern is then whether VirTues provides an advance over current approaches for each of the tasks it addresses. In other words, if contemplating how to analyze data from a new project, would one expect better results from VirTues or a specialized tool or tools?

We thank the reviewer for raising this question, which motivated the most substantial new analysis in this revision. A foundation model can advance spatial proteomics in two ways: by enabling analyses that existing specialized tools cannot perform, and by improving the ones they already do. The original submission established the first, including cross-cohort spatial biomarker discovery in a shared tissue representation. The reviewer's question concerns the second, and addressing it has made the manuscript considerably stronger.

Concretely, in this rebuttal, we further benchmark VirTues against specialized tools on cell segmentation and cell typing. We chose these two tasks deliberately: they are the first steps of essentially every current spatial proteomics analysis, and every downstream result, from cell states and tissue niches to biomarker signatures, inherits their errors. Conversely, every improvement at this stage propagates through the entire workflow. They also probe orthogonal competencies: segmentation tests dense, pixel-level structural reasoning, while cell typing tests semantic integration of multi-channel marker expression into cell type labels. Lastly, these are tasks for which strong specialized tools already exist, which makes the comparison a demanding benchmark.

We evaluated VirTues across nine spatial proteomics datasets in a zero-shot setting, i.e., without training on the target datasets, thereby mirroring the practical scenario in which a newly imaged dataset requires annotation. As baselines we compare against CellPose (Stringer et al., 2021), InstanSeg (Goldsborough et al., 2024), and StarDist (Stevens et al., 2022) for segmentation, and MAPS (Shaban et al., 2024) and Astir (Geuenich et al., 2021) for cell typing, as well as combined

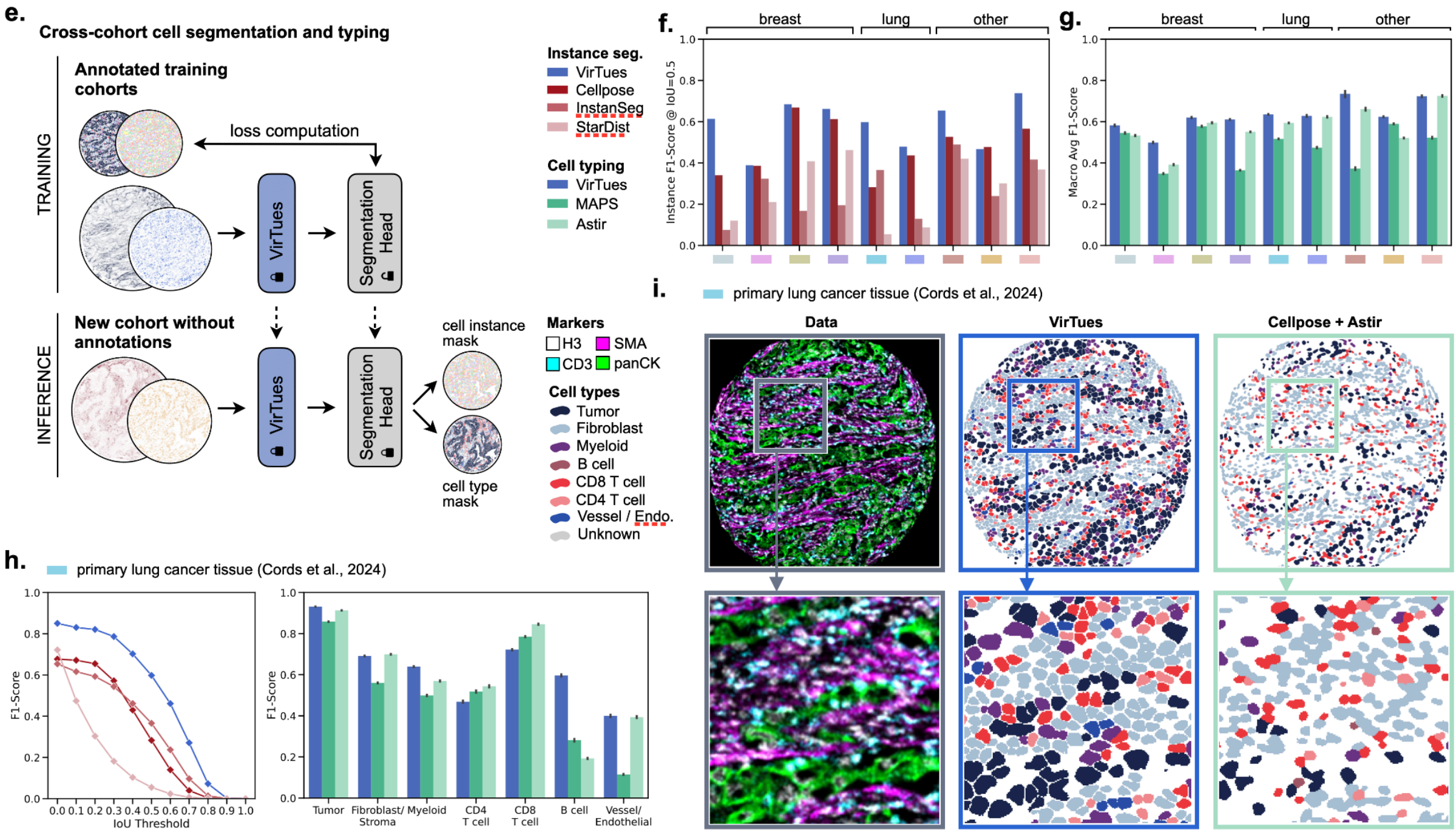

Fig. R2. **Cross-cohort cell segmentation and typing benchmark (updated Figure 3 from the manuscript). e,** Workflow: VirTues is trained on annotated cohorts and applied zero-shot to new cohorts without dataset-specific fine-tuning, producing cell-instance masks and cell-type masks in a single pass. **f,** Instance F1-score (IoU = 0.5) for cell segmentation across breast, lung, and other tumor cohorts, comparing VirTues to CellPose, InstanSeg, and StarDist. **g,** Macro-averaged F1-score for cell typing across the same cohorts, comparing VirTues to MAPS and Astir. **h,** F1-score as a function of IoU threshold on primary lung cancer tissue (Cords et al., 2024). **i,** Representative predictions on primary lung cancer tissue: marker channels (H3, SMA, CD3, panCK), VirTues output, and the CellPose + Astir pipeline, with cell-type masks for tumor, fibroblast, myeloid, B cell, CD8 T cell, CD4 T cell, and vessel/endothelial.

pipelines such as CellPose with Astir. In a single zero-shot pass, VirTues produces both cell-instance and cell-type masks (Fig. R2e), and outperforms every baseline: on segmentation (Fig. R2f), on cell typing (Fig. R2g), and against the combined CellPose and Astir pipeline (Fig. R2i). Detailed dataset-wise and class-wise results are reported in Figs. S24 and S25.

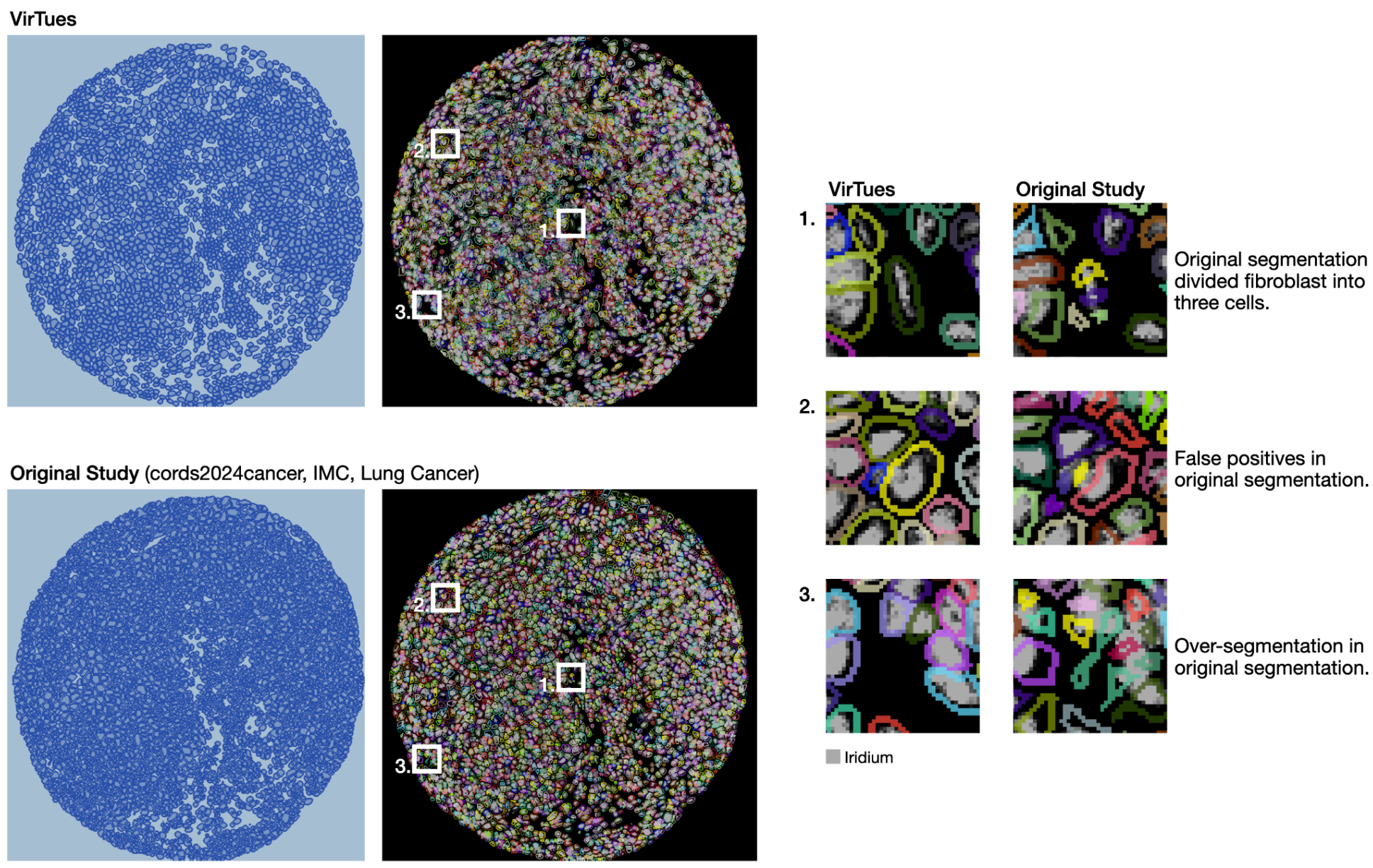

Fig. R3. Comparison of cell instance segmentations from VirTues and the original study by Cords et al. (2024) on primary lung cancer tissue. Top: VirTues prediction. Bottom: reference segmentation from the original study. Three highlighted regions illustrate recurrent limitations of the reference annotations: 1. A single fibroblast split into multiple cells, 2. false-positive cell instances, and 3. over-segmented tissue regions. In each case, the VirTues segmentation more coherently matches the underlying marker signal.

The comparison also revealed something we had not anticipated, which highlights the limitations of current spatial proteomics analyses: The segmentation masks provided by the original studies vary substantially in quality. In several cases, the segmentations produced by VirTues match the underlying data more coherently than the original annotations, including cases where the original

masks split one fibroblast into multiple cells, introduce false-positive cell instances, or over-segment cell areas (Figs. R3 and S23). The lower Instance F1-scores at IoU = 0.5 on some datasets (Fig. R2h) therefore partly reflect limitations of the available reference annotations rather than failures of VirTues.

The same applies to cell type annotations. In several examples, VirTues' predictions match the marker signals more coherently than the original labels (Figs. R4 and S26). On primary breast cancer tissue from Wang et al. (2023), reference annotations classify cells as CD8 T cells despite the absence of CD8 signal, with analogous discrepancies for B cells and fibroblasts. As with segmentation, the cell typing F1-scores on these datasets therefore partly reflect limitations in the reference annotations rather than VirTues falling short.

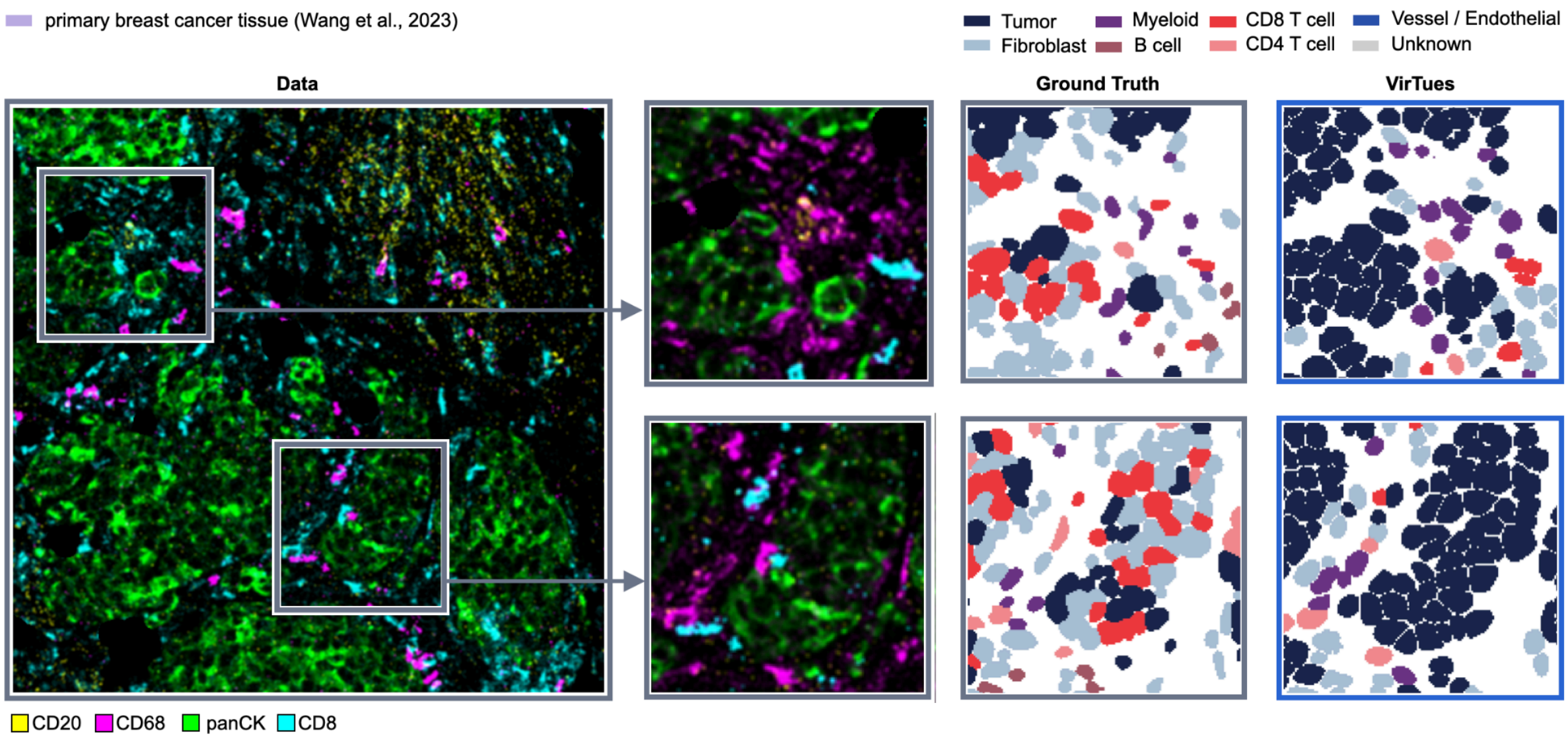

Fig. R4. Comparison of cell-type annotations on primary breast cancer tissue (Wang et al., 2023). Left: multiplexed marker channels (CD20, CD68, panCK, CD8) for two representative regions. Center: original ground-truth cell type labels. Right: VirTues zero-shot predictions. In both regions, the VirTues predictions track the underlying marker signal more coherently than the reference annotations, particularly for B cells (CD20+) and CD8 T cells (CD8+).

These benchmarks provide a direct answer to the reviewer's question: for a new project, researchers can apply VirTues to a new multiplexed dataset, even with a different marker panel, and obtain segmentation and cell typing in a single zero-shot pass that is more accurate than assembling and tuning specialized tools. Combined with the capabilities reported in the original manuscript, this establishes VirTues as a unified computational entry point for spatial proteomics analyses.

> The discussion provides some comments regarding limitations, but the impression that is left is that a potential user would need to carry out their own comparison to other tools. For example, the previous review pointed out the issue of reliance on visual evaluation. New results are presented in Figures S12 and S12 to address this, but those results show large variability in performance for different markers and tissues. They do not give confidence that future uses of VirTues would be reliable for a chosen application.

We thank the reviewer for this point, and we see how the presentation could have left this impression. We have revised the text and figures to make one distinction explicit. Figs. S2 and S3 (previously S12 and S13 in the earlier manuscript that the reviewer referred to) evaluate a single capability, namely held-out marker reconstruction, in which a marker is removed from the input and VirTues infers its spatial distribution from the remaining panel and tissue context. They are not a general measure of how reliably VirTues performs its downstream applications.

The variability across markers in these figures is expected, and it is informative rather than a sign of unreliability. Whether a held-out protein can be reconstructed depends on whether the rest of the panel carries information about it. Markers tied to co-expression patterns, morphology, or spatial organization are reconstructed well; markers that report independent biological variation, such as context-specific signaling or rare functional states, are by their nature harder to infer once removed. This marker-dependence reflects the spatial co-dependence of proteins in tissue, a biological property, not a deficiency of the model.

Reliability for a chosen application is established by that application's own evaluation (e.g., across Fig. 3-5 and corresponding Supp. Figs.), not by held-out marker reconstruction (i.e., Figs. S2 and S3). Concretely, segmentation, cell typing (Fig. 3), tissue annotation, retrieval (Fig. 4), and biomarker discovery (Fig. 5) are each benchmarked separately with task-specific metrics and baselines. The new cross-cohort segmentation and cell typing benchmark added in this revision is a direct example: VirTues is more accurate than specialized tools across nine datasets, zero-shot (updated Fig. 3e-j).

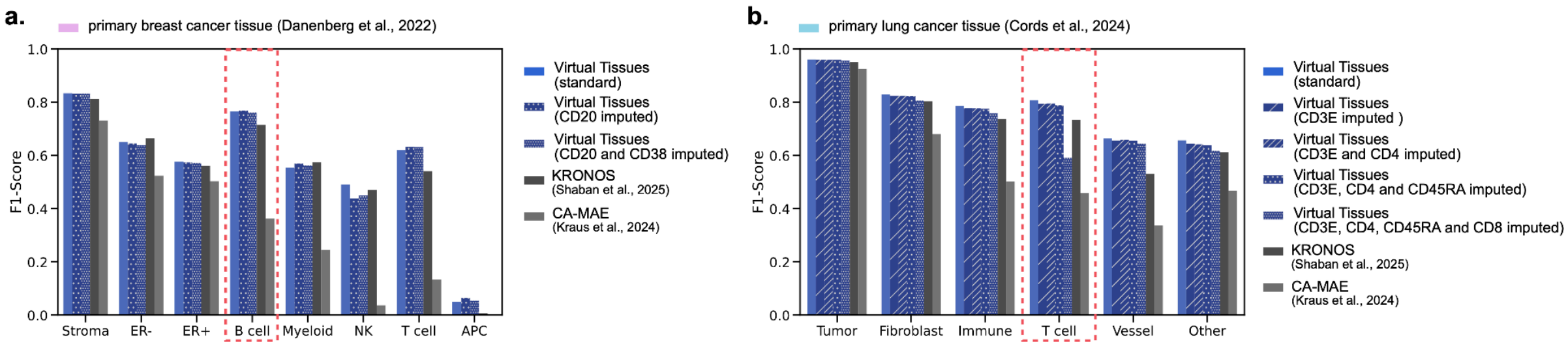

Figure R5. Evaluation of virtual staining for downstream cell phenotyping under marker-panel perturbations (from prior Response to Reviewer 2). VirTues reconstructs subsets of markers, and the resulting virtual stains are incorporated into a partially virtual multiplex image for cell phenotyping. Performance is reported as class-wise F1-score; for reference, the same evaluation using VirTues, KRONOS, and CA-MAE on the full multiplex image with all real channels is also shown. **a,** On Danenberg et al. (2022), cell typing performance with VirTues-predicted (i) CD20 and (ii) CD20 and CD38. Performance of VirTues w.r.t. F1-scores remains stable for B cells, which are primarily identified by these markers. **b,** On Cords et al. (2024), cell typing performance with VirTues-predicted (i) CD3E, (ii) CD3E and CD4, (iii) CD3E, CD4, and CD45RA, and (iv) CD3E, CD4, CD45RA, and CD8. Performance of VirTues w.r.t. F1-scores remains stable for T cells, which are primarily identified by these markers.

This reliability holds even when markers are missing from a dataset entirely, a common situation across studies with different panels and a point raised by Reviewer 2 in an earlier review. To test the robustness of VirTues and the virtual tissue representations, we evaluated cell phenotyping with key markers including CD3 and CD8 withheld from the input and replaced by VirTues' reconstructions (Fig. R5). Cell typing performance remained stable, including for the cell types those markers primarily define. This supports the virtual tissue argument: VirTues encodes the biological context in which markers occur, not only the markers themselves, and can recover cellular identity even when a defining marker is unavailable.

Finally, on the earlier point about visual evaluation: marker reconstruction was assessed quantitatively, in Fig. 2 and Figs. S2 and S3 (previously S12 and S13), with representative images included for illustration purposes. We have revised the presentation to foreground these quantitative results.

> There is also little methodological innovation since it uses well established methods.

We appreciate the opportunity to clarify the methodological contribution. While some of the core building blocks underlying VirTues, such as masked autoencoding and transformer architectures, are well established, the contribution does not lie in introducing these components themselves. Rather, the novelty lies in integrating and extending them to enable foundation modeling for spatial proteomics, a domain in which these approaches do not directly transfer and substantial methodological innovation was required to be effective. Standard vision transformers assume fixed input channels, such as RGB, or a fixed assay panel. Masked autoencoders developed for histopathology or low-plex Cell Painting do not handle the defining features of multiplexed spatial proteomics: high-plex marker panels that

differ between studies, missing markers, and the need to learn jointly over marker identity and spatial context.

Addressing this required new components: marker-aware tokenization, protein identity embeddings, sparse attention adapted to multiplex images, and a hierarchical design that yields patch-, cell-, niche-, and tissue-level representations, together with a marker-aware masked reconstruction objective and masking strategies for heterogeneous panels. These are what allow a single model to operate across marker panels and technologies and to support multi-scale tasks that would otherwise each require a separate specialized pipeline. The novelty of this method has been recognized independently, including a Best Paper Award at the ICLR 2025 Workshop on Learning Meaningful Representations of Life.

No existing method spans the same range of functionality, from held-out marker reconstruction to segmentation, cell typing, tissue annotation, retrieval, and cross-cohort biomarker discovery. The comparison in the manuscript is therefore between a unified foundation model and a set of narrower task-specific tools. If the reviewer is aware of prior work that establishes foundation modeling for spatial proteomics under variable marker panels, we would welcome the reference and will discuss it in the manuscript.

References

Bommasani et al. "On the opportunities and risks of foundation models." arXiv preprint arXiv:2108.07258 (2021).

Chen, Richard J., et al. "Towards a general-purpose foundation model for computational pathology." Nature Medicine 30.3 (2024): 850-862.

Leslie. "The silicon cell." Science 390.6772 (2025).

Bunne et al. "How to build the virtual cell with artificial intelligence: Priorities and opportunities." Cell 187.25 (2024): 7045-7063.

Cheng et al. "Harnessing AI to Build Virtual Cells." bioRxiv (2026): 2026-04.

Oquab, Maxime, et al. "Dinov2: Learning robust visual features without supervision." arXiv preprint arXiv:2304.07193 (2023).

Stringer et al. "Cellpose: a generalist algorithm for cellular segmentation." Nature Methods 18.1 (2021): 100-106.

Goldsborough et al. "InstanSeg: an embedding-based instance segmentation algorithm optimized for accurate, efficient and portable cell segmentation." arXiv preprint arXiv:2408.15954 (2024).

Stevens et al. "StarDist image segmentation improves circulating tumor cell detection." Cancers 14.12 (2022): 2916.

Shaban et al. "MAPS: pathologist-level cell type annotation from tissue images through machine learning." Nature Communications 15.1 (2024): 28.

Geuenich et al. “Automated assignment of cell identity from single-cell multiplexed imaging and proteomic data.” Cell Systems 12.12 (2021): 1173-1186.
